# Biomarkers of environmental enteric dysfunction are not consistently associated with linear growth velocity in rural Zimbabwean infants

**DOI:** 10.1093/ajcn/nqaa416

**Published:** 2021-03-19

**Authors:** Kuda Mutasa, Robert Ntozini, Mduduzi N N Mbuya, Sandra Rukobo, Margaret Govha, Florence D Majo, Naume Tavengwa, Laura E Smith, Laura Caulfield, Jonathan R Swann, Rebecca J Stoltzfus, Lawrence H Moulton, Jean H Humphrey, Ethan K Gough, Andrew J Prendergast

**Affiliations:** Zvitambo Institute for Maternal and Child Health Research, Harare, Zimbabwe; Zvitambo Institute for Maternal and Child Health Research, Harare, Zimbabwe; Zvitambo Institute for Maternal and Child Health Research, Harare, Zimbabwe; Global Alliance for Improved Nutrition, Washington, DC, USA; Zvitambo Institute for Maternal and Child Health Research, Harare, Zimbabwe; Zvitambo Institute for Maternal and Child Health Research, Harare, Zimbabwe; Zvitambo Institute for Maternal and Child Health Research, Harare, Zimbabwe; Zvitambo Institute for Maternal and Child Health Research, Harare, Zimbabwe; Zvitambo Institute for Maternal and Child Health Research, Harare, Zimbabwe; Department of Epidemiology and Environmental Health, School of Public Health and Health Professions, University at Buffalo, Buffalo, NY, USA; Department of International Health, Johns Hopkins Bloomberg School of Public Health, Baltimore, MD, USA; Division of Digestive Diseases, Department of Metabolism, Digestion, and Reproduction, Faculty of Medicine, Imperial College London, London, United Kingdom; School of Human Development and Health, Faculty of Medicine, University of Southampton, Southampton, United Kingdom; Program in International Nutrition, Division of Nutritional Sciences, Cornell University, Ithaca, NY, USA; Department of International Health, Johns Hopkins Bloomberg School of Public Health, Baltimore, MD, USA; Zvitambo Institute for Maternal and Child Health Research, Harare, Zimbabwe; Department of International Health, Johns Hopkins Bloomberg School of Public Health, Baltimore, MD, USA; Department of International Health, Johns Hopkins Bloomberg School of Public Health, Baltimore, MD, USA; Zvitambo Institute for Maternal and Child Health Research, Harare, Zimbabwe; Blizard Institute, Queen Mary University of London, London, United Kingdom

**Keywords:** environmental enteric dysfunction, biomarkers, child growth, stunting, Zimbabwe

## Abstract

**Background:**

Child stunting remains a poorly understood, prevalent public health problem. Environmental enteric dysfunction (EED) is hypothesized to be an important underlying cause.

**Objectives:**

Within a subgroup of 1169 children enrolled in the SHINE (Sanitation Hygiene Infant Nutrition Efficacy) trial in rural Zimbabwe, followed longitudinally from birth to 18 mo of age, we evaluated associations between the concentration of 11 EED biomarkers and linear growth velocity.

**Methods:**

At infant ages 1, 3, 6, 12, and 18 mo, nurses measured child length and collected stool and blood; the lactulose-mannitol urine test was also conducted at all visits except at 1 mo. Stool neopterin, α-1 antitrypsin, myeloperoxidase, and regenerating gene 1β protein; urinary lactulose and mannitol; and plasma kynurenine, tryptophan, C-reactive protein, insulin-like growth factor-1 (IGF-1), soluble CD14, intestinal fatty acid binding protein, and citrulline were measured. We analyzed the change in relative [∆ length-for-age *z* score (LAZ)/mo] and absolute (∆ length/mo) growth velocity during 4 age intervals (1–3 mo; 3–6 mo; 6–12 mo; and 12–18 mo) per SD increase in biomarker concentration at the start of each age interval.

**Results:**

In fully adjusted models, we observed only 3 small, statistically significant associations: kynurenine:tryptophan ratio at 12 mo was associated with decreased mean LAZ velocity during the 12–18 mo interval (−0.015 LAZ/mo; 95% CI: −0.029, −0.001 LAZ/mo); mannitol excretion at 6 mo was associated with increased LAZ velocity during the 6–12 mo interval (0.013 LAZ/mo; 95% CI: 0.001, 0.025 LAZ/mo), and plasma IGF-1 at 1 mo was associated with increased LAZ velocity during the 1–3 mo interval (0.118 LAZ/mo; 95% CI: 0.024, 0.211 LAZ/mo). Results for absolute growth velocity were similar, except IGF-1 was also associated with growth during the 12–18 mo interval. We found no other associations between any EED biomarker and linear growth velocity.

**Conclusions:**

None of 11 biomarkers of EED were consistently associated with linear growth among Zimbabwean children.

This trial was registered at clinicaltrials.gov as NCT01824940.

## Introduction

Globally, 21% of children <5 y of age (149 million) are stunted (1), defined as having a length or height >2 SDs below the age- and sex-matched reference population median (2). Deficits in linear growth largely accrue from conception to 24 mo of age (3), corresponding to the period when normal child growth and development are most rapid. Stunting is associated with reduced child survival, neurodevelopment, and educational attainment, and with reduced economic productivity during adulthood (4–6).

A complex network of underlying determinants, including socioeconomic factors (5, 7, 8), maternal nutrition (9–11), maternal education (12), low birth weight (13), infectious diseases (especially enteric) (8, 14, 15), and inadequate diet (8, 16), drive stunting. Interventions to reduce stunting have largely focused on improving complementary feeding. However, these interventions have only achieved a modest impact on growth (17, 18). To restore normal growth and eliminate stunting, causal mechanisms must be more clearly defined and novel targets for interventions identified.

Three decades ago, tropical enteropathy was proposed as an important factor in undernutrition (19, 20). This small intestinal pathology, now called environmental enteric dysfunction (EED), has gained considerable traction as a plausible cause of linear growth faltering (21, 22). EED is a subclinical disorder of the gut, characterized by reduced villus height, increased crypt depth, and lymphocytic infiltration (21), resulting in impaired absorption and increased small intestinal permeability. Loss of intestinal barrier function enables microbial translocation resulting in systemic inflammation. Raised proinflammatory cytokines suppress plasma concentrations of insulin-like growth factor 1 (IGF-1), thereby restraining linear growth (23, 24). EED is acquired early in life among children living in impoverished conditions (25–27). Chronic exposure to enteric pathogens is likely the major cause of EED, although nutrient deficiencies and fungal toxin exposure are also potential causative or predisposing factors (26, 28, 29).

Because endoscopy to collect intestinal biopsy samples is rarely feasible in young children, numerous biomarkers of EED measured in urine, blood, and stool have been proposed; each is intended to reflect a specific component of the hypothesized causal pathway between enteropathogen exposure and the growth plate (23, 26, 30). Some prior studies have defined EED with a single biomarker (31, 32) or small numbers of biomarkers (33–35), some with novel biomarkers (36), whereas others have included large panels of biomarkers (9, 37, 38). Findings from these studies have been highly heterogeneous: for every biomarker, there is evidence both supporting and not supporting its association with linear growth (30).

The SHINE (Sanitation Hygiene Infant Nutrition Efficacy) trial was a cluster-randomized trial that tested the impact of an improved household water, sanitation, and hygiene (WASH) intervention and an improved infant and young child feeding (IYCF) intervention on attained linear growth at 18 mo of age (39). The SHINE trial was based on the hypothesis that EED is a major underlying cause of stunting, and that the WASH intervention would improve growth, primarily through reducing EED (40). However, the low-cost household-level WASH intervention tested in SHINE had no impact on enteropathogen carriage (41), EED biomarkers (42), or linear growth (43, 44). In this article, we investigate the association between EED biomarkers and linear growth among HIV-unexposed infants (outside the randomized trial design) to explore whether SHINE provides observational evidence that EED underlies linear growth faltering.

## Methods

The SHINE trial (NCT01824940) design and methods (39) and primary outcomes (43, 44) have been published elsewhere; the protocol and statistical analysis plan are available at: https://osf.io/w93hy. Briefly, SHINE randomly assigned clusters, defined as the catchment area of 1–4 village health workers (VHWs) employed by the Ministry of Health and Child Care, to receive 1 of 4 interventions: IYCF, WASH, IYCF + WASH, or Standard of Care. The IYCF intervention included a small-quantity lipid-based nutrient supplement for the infant to consume daily between 6 and 18 mo of age and counselling on complementary feeding. The WASH intervention included services which are most commonly provided to people in rural areas of low- and middle-income countries (LMICs): a ventilated improved pit latrine, 2 handwashing stations, monthly delivery of liquid soap and chlorine, a clean play space to reduce geophagia by separating children from domestic animals and loose dirt, and behavior change modules promoting use of these tools. Between 22 November, 2012 and 27 March, 2015, pregnant women were enrolled after providing written informed consent. VHWs delivered intervention-specific lessons during 15 home visits between enrollment and 12 mo postpartum.

Research nurses made home visits twice during pregnancy and at infant ages 1, 3, 6, 12, and 18 mo. At baseline, maternal education and age, household wealth (45), existing water and sanitation services, and household food security (46) were assessed, and mothers were tested for HIV via a rapid testing algorithm. Infant birth date, weight, and delivery details were transcribed from health facility records. Gestational age at delivery was calculated from the date of the mother's last menstrual period ascertained at baseline. Infant weight, length, and midupper arm circumference were measured at every postnatal visit. Nurses were standardized against a gold-standard anthropometrist every 6 mo, with retraining provided to those who failed to meet predefined criteria. Given the household-based nature of the trial interventions, home visits were not conducted if the mother was not available in the household where she consented, except for the 18-mo visit (trial endpoint) when follow-up was conducted anywhere within Zimbabwe.

### EED substudy

Between 1 May, 2014 and 27 March, 2015, mothers enrolling into the SHINE trial were invited to join a substudy to investigate biomarkers of EED. Women were informed about the substudy during pregnancy and those with live births were enrolled after providing additional written informed consent. From children enrolled in the EED substudy, stool (passed on the morning of the research visit and collected by the mother into a plain container) and blood (collected by venipuncture into an EDTA-coated tube) were collected at all postnatal visits. In addition, at the 3, 6, 12, and 18 mo visits, the lactulose–mannitol test was undertaken. After a 30-min fast, infants ingested 2 mL/kg body weight (maximum 20 mL) of a sterile solution containing 250 mg lactulose/mL and 50 mg mannitol/mL; a urine bag was placed, and all urine passed over a 2-h period was collected and preserved with chlorhexidine. Although 5-h urine collections have been used in many previous studies, the lactulose recovery in a 2-h collection better reflects small intestinal permeability (47). Stool and urine samples were transported in a cool box and blood samples were transported at room temperature to the field laboratory where EDTA-coated tubes were centrifuged at room temperature for 5 min at 291 ×*g*to collect plasma, stool specimens were divided into aliquots in cryotubes, and urine specimens were divided into aliquots in cryotubes after measuring total volume. All specimens were stored in the field laboratory at −80°C until subsequent transfer to the Zvitambo Laboratory in Harare for long-term storage at −80°C until analysis. A sample size of 150 infants/arm (600 total) was originally calculated to be sufficient to detect effect sizes of 35%–40% of 1 SD of each biomarker with 80% power, and α = 0.05; however, we chose to recruit 1000 infants to allow for missing or inadequate specimen collection during home visits.

### Biomarkers of EED

We chose biomarkers indicative of 5 domains of the structural, functional, and metabolic changes characteristic of EED: altered gut architecture, intestinal inflammation, impaired epithelial regeneration, increased permeability, and microbial translocation; and 2 domains of systemic sequelae: inflammation and suppression of the growth hormone axis (**Table 1**) (23, 48–55). All ELISA assays were conducted in the Zvitambo laboratory in Harare, Zimbabwe; MS was undertaken at Oregon Analytic Oregon, USA for lactulose and mannitol measurement, and at Imperial College, UK for citrulline, kynurenine, and tryptophan, using previously published methods (42).

**TABLE 1:** Domains of EED and its sequelae, corresponding biomarkers, and assays used in the SHINE (Sanitation Hygiene Infant Nutrition Efficacy) trial^1^

			Assay	
	Biomarker	Rationale and previous reports	Platform and manufacturer	Limit of detection
EED domain				
Altered intestinal architecture	Plasma intestinal fatty acid binding protein	Found mostly at tips of small intestinal villi. Released into circulation after epithelial damage (48)	ELISA, Hycult Biotechnology, Uden, Netherlands	47 pg/mL
	Plasma citrulline	Biomarker of total intestinal mass (38, 49)	Chromatography tandem MS with electrospray ionization, Waters, Wilmslow, United Kingdom	100 ng/mL
Impaired intestinal regeneration	Fecal regenerating gene 1β	Stool regenerating gene protein, which is a measure of intestinal injury and repair (36, 38)	ELISA, TECHLAB Inc, Blacksburg, VA, USA	0.625 ng/mL
Intestinal permeability	Fecal α-1 antitrypsin	Indicates leakage of plasma protein into intestine (35)	ELISA, BioVendor, Brno, Czech RepublicProminence LC (Shimadzu) with mass spectrometer with electrospray ionization (Restek column SciexQTRAP5000)	1.5 ng/mL1 ng/mL for both sugars
	Urinary lactulose:mannitol ratio	Uptake of large sugar (lactulose) through impaired intestinal barrier and reduced mannitol absorption due to reduced surface area (20)		
Microbial translocation	Plasma soluble CD14	Marker of LPS stimulation of monocytes (50)	ELISA, R&D Systems, Minneapolis, MN, USA	125 pg/mL
Intestinal inflammation	Fecal myeloperoxidase	Biomarker of gut inflammation (51, 53)	ELISA, Immundiagnostik, Bensheim, Germany	1.6 ng/mL
	Fecal neopterin	Biomarker of gut inflammation (53)	ELISA, GenWay Biotech, Inc., San Diego, CA, USA	0.7 nmol/L
Systemic sequelae				
Systemic inflammation	Plasma C-reactive protein	Acute-phase protein	ELISA, R&D Systems, Minneapolis, MN, USA	0.01 ng/mL
	Plasma kynurenine-to-tryptophan ratio	Induction of IDO enzyme in response to inflammation (52)	Ultra-high performance LC tandem MS with electrospray ionization, Waters, Wilmslow, United Kingdom	40 ng/mL (kynurenine)200 ng/mL (Trp)
Growth-hormone axis	Plasma insulin-like growth factor 1	Required at growth plate to mediate effects of growth hormone (54, 55)	ELISA, R&D Systems, Minneapolis, MN, USA	0.026 ng/mL

1 EED, environmental enteric dysfunction; IDO, Indoleamine-pyrrole 2,3-dioxygenase.

### Statistical methods

Lactulose and mannitol excretion fractions were calculated as:

(1) \begin{eqnarray*} {\rm{\% \ marker\ excretion\ }} &=& \frac{{{\rm{excreted\ marker}}\left( {{\rm{mg}}} \right) \times 100}}{{{\rm{ingested\ marker\ }}\left( {{\rm{mg}}} \right){\rm{\ }}}}{\rm{\ }}\nonumber\\ &=& \frac{{a\left( {b/1000} \right){\rm{\ }}}}{{cd}} \times 100 \end{eqnarray*}

Where: *a* = postingestion urine marker concentration (mg/L); *b* = volume of urine obtained (mL); *c* = volume of oral lactulose–mannitol solution ingested (mL); and *d* = 250 where the marker was lactulose, or 50 where the marker was mannitol.

Lactulose-to-mannitol ratio (LMR) was estimated using the following formula:

(2) \begin{eqnarray*} {\rm{LMR }} = \frac{{{\rm{\% \ lactulose\ excretion\ }}}}{{{\rm{\% \ mannitol\ excretion}}}} \end{eqnarray*}

The distributions of all EED biomarker values were skewed to high and normalized by log transformation. We also calculated a previously proposed composite index combining fecal myeloperoxidase, α-1 antitrypsin (A1AT), and neopterin [environmental enteropathy score (EE score)] (35, 56).

Length measurements at each time point were converted to length-for-age *z* scores (LAZs) based on WHO growth standards (57). Infant linear growth velocity during the 4 age intervals between each pair of scheduled postnatal visits (1–3 mo, 3–6 mo, 6–12 mo, and 12–18 mo) was calculated in 2 ways to reflect the relative (LAZ_End_ − LAZ_start_)/(Age_End_ − Age_Start_) and absolute [Length (cm)_End_ − Length (cm)_Start_]/(Age_End_ − Age_Start_) growth velocity during the interval.

The unadjusted association between linear growth velocity and EED was investigated by fitting separate simple linear regression models for each EED biomarker (expressed as SDs from its mean) during each interval:

(3) \begin{eqnarray*} E\ \frac{{{\rm{LA}}{{\rm{Z}}_{{\rm{end}}}} - {\rm{LA}}{{\rm{Z}}_{{\rm{start}}}}}}{{{\rm{Ag}}{{\rm{e}}_{{\rm{end}}}} - {\rm{Ag}}{{\rm{e}}_{{\rm{start}}}}}} = {\beta _0}\ + {\beta _1} \times {\rm{EED\ marke}}{{\rm{r}}_{{\rm{start}}}} \end{eqnarray*}

The models were repeated substituting length (cm) for LAZ.

To investigate whether EED independently explained linear growth, we used multivariable linear regression. Separate models for each biomarker during each age interval were fitted to estimate change in linear growth over the interval per SD increase in biomarker concentration assessed at the start of the age interval using this form:

(4) \begin{eqnarray*} && E\left[ {{\rm{LAZ}}_{\rm{end}} - {\rm{LAZ}}_{\rm{start}}} \over {{\rm{Age}}_{\rm{end}} - {\rm{Age}}_{\rm{start}}} \right]\nonumber\\ &&\quad = {\beta _0}\; + {\beta _1} \times {\rm{Infant\;Sex}} + {\beta _2}\nonumber\\ &&\qquad \times\, {\rm{Infant\;Ag}}{{\rm{e}}_{{\rm{start}}}} + {\rm{\;}}{\beta _3} \times {\rm{EED\;marke}}{{\rm{r}}_{{\rm{start}}}}\nonumber\\ &&\qquad +\, \mathop \sum \limits_i {\beta _i} \times {\rm{adjustment\;covariat}}{{\rm{e}}_i} \end{eqnarray*}

Minimally adjusted models included infant sex and age at the start of the growth interval. Fully adjusted models also included covariates selected for each model from a set of prespecified candidate variables (**Supplemental Text 1**) by best subset selection. Briefly, all possible combinations of the prespecified candidate variables were searched, using an efficient branch and bound algorithm, to identify the subset of variables that best explained growth velocity during a follow-up interval (58). The Akaike Information Criterion (AIC) was used to define the most explanatory subset of covariates (58). Multivariable models were also repeated following the aforementioned form, but substituting length (cm) for LAZ.

The robustness of model results to influential outlier observations was assessed by refitting each model after 95% winsorization of biomarker SDs from the mean at the start of each interval (59). In a sensitivity analysis to control for the effect of previous growth faltering on the association between EED and subsequent growth velocity, we refitted each model after excluding infants with stunting (LAZ < −2.0) at the start of each interval. We explored effect modification by infant sex with stratified analyses when the interaction term between sex and the biomarker in a regression for LAZ had a *P* value < 0.1; the same procedure was followed for potential effect modification by the IYCF treatment group.

To visualize trends in linear growth velocity by infant age and sex, we fitted generalized additive models of LAZ velocity and length velocity against infant age at the end of each follow-up interval using cubic splines with 3–5 knots for smoothing, chosen by AIC, and stratified by child sex. Weight-for-height *z* score (WHZ) velocity was not a focus of the article but has been calculated and included as supplementary data.

In further analyses we implemented analytic approaches used by other studies, for comparability. These included defining the exposure at each time point in different ways to enable comparison across studies: *1*) as quartiles of biomarker concentration (54), *2*) as 2 quantiles of biomarker concentration (38), *3*) fourth quartile and interquartile range (second + third quartiles) compared with the first quartile of biomarker concentration (51), and *4*) the mean of biomarker concentration at all time points, after detrending for age and breastfeeding status (60). We also implemented the method used by the MAL-ED (The Etiology, Risk Factors, and Interactions of Enteric Infections and Malnutrition and the Consequences for Child Health and Development Project) study to evaluate the associations between EED biomarkers and linear growth by 18 mo of age, where the outcome was defined as LAZ divided into the ranges <−2, ≥−2 to < −1, or ≥ −1, and the measure of association was the cumulative odds of being in a lower LAZ category, when exposure was defined as the 75^th^ compared with the 25^th^ biomarker quartile (8).

Statistical significance was evaluated at α = 0.05. All analyses were conducted in R version 3.5.3 (Free Software Foundation). Best subset selection was performed using the function *lmSubsets()* in the package lmSubsets (61). Given the exploratory nature of these analyses, we prespecified in our statisical analysis plan that we would not account for multiple comparisons when reporting *P* values or CIs.

### Ethics and regulatory oversight

The SHINE trial and the EED substudy were approved by the Medical Research Council of Zimbabwe and the Institutional Review Board of the Johns Hopkins Bloomberg School of Public Health.

## Results

**FIGURE 1:**
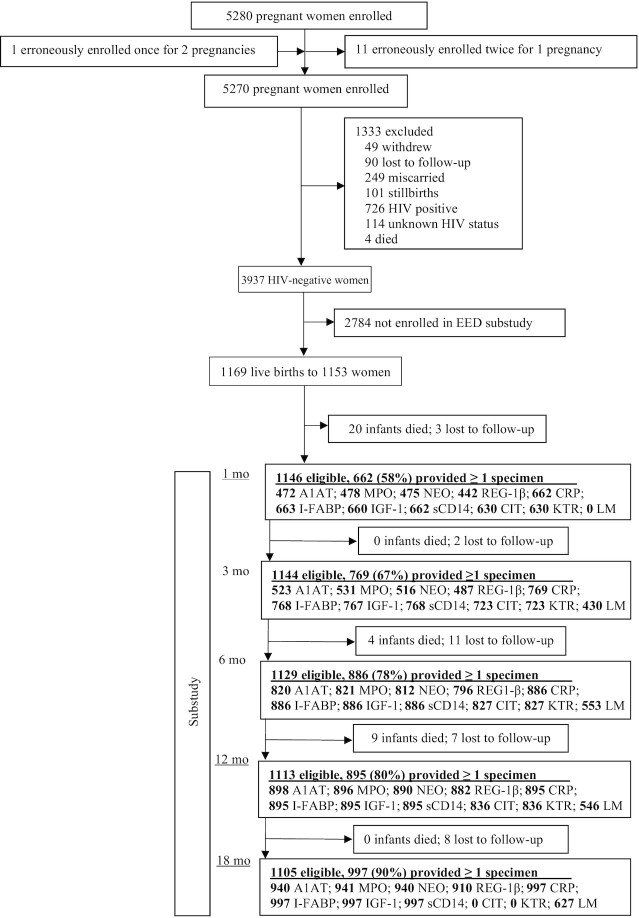
Flow of participants through the trial. A1AT, fecal α-1 antitrypsin; CIT, plasma citrulline; CRP, plasma C-reactive protein; EED, environmental enteric dysfunction; I-FABP, plasma intestinal fatty acid binding protein; IGF-1, insulin-like growth factor 1; KTR, kynurenine-to-tryptophan ratio; LM, lactulose–mannitol; LMR, lactulose-to-mannitol ratio (urinary); MPO, fecal myeloperoxidase; NEO, fecal neopterin; REG-1β, fecal regenerating gene 1β; sCD14, plasma soluble CD14.

Of 5270 women enrolled in the SHINE trial, there were 3989 live births to 3937 HIV-negative mothers. Among these dyads, 1153 mothers were enrolled into the trial during the EED substudy recruitment period and agreed for their 1169 infants to join the substudy. Of these, 33 infants (2.8%) died and 31 (2.7%) were lost to follow-up or withdrew before the 18 mo visit (**Figure 1**). Follow-up was lowest at the 1-mo study visit when many women were away from home living with extended family in the immediate postnatal period.

At baseline, about one-third of households in the EED substudy had improved sanitation and two-thirds obtained drinking water from an improved source. Women were generally of good nutritional status and well-educated: mean ± SD midupper arm circumference was 26.7 ± 3.3 cm, and years of schooling 9.6 ± 1.8 (**Table 2**). Among infants, 15% were born preterm and 8% were low birth weight (Table 2). Compared with mother–infant dyads not enrolled in the EED substudy, those enrolled had a higher household minimum dietary diversity score and a lower proportion of infants born preterm; other characteristics were similar (**Supplemental Table 1**). The baseline characteristics of infants enrolled in the EED substudy who provided specimens at each time point were similar to those who did not (Supplemental Table 1).

**TABLE 2:** Maternal, household, and infant baseline characteristics of HIV-negative mothers and live-born infants enrolled in the SHINE (Sanitation Hygiene Infant Nutrition Efficacy) environmental enteric dysfunction substudy^1^

Baseline characteristic	Value
Mothers, *n*	1153
Infants, *n*	1169
Household characteristics	
Occupants, *n*	5 [3–6]
Wealth quintile	
1 (lowest)	199 of 1124 (17.7%)
2	229 of 1124 (20.4%)
3	237 of 1124 (21.1%)
4	232 of 1124 (20.6%)
5 (highest)	227 of 1124 (20.2%)
Improved latrine at household	343 of 1100 (31.2%)
Main source of household drinking water improved	690 of 1103 (62.6%)
Household meets Minimum Dietary Diversity Score	472 of 1003 (47.1%)
Coping Strategies Index score	0 [0–5]
Maternal characteristics	
Age, y	26.5 ± 6.7
Height, cm	160.0 ± 7.7
Midupper arm circumference, cm	26.7 ± 3.3
Years of schooling completed	9.6 ± 1.8
Married	1070 of 1131 (94.6%)
Religion	
Apostolic	550 of 1138 (48.3%)
Other Christian	514 of 1138 (45.2%)
Other	74 of 1138 (6.5%)
Ever booked antenatal care	1060 of 1068 (99.3%)
Maternal anemia at baseline (Hb <12 g/dL)	158 of 1013 (15.6%)
Infant characteristics	
Female sex	567 of 1167 (48.6%)
Birth weight, kg	3.1 ± 0.5
Birth weight <2500 g	89 of 1113 (8.0%)
Preterm <37 weeks of gestation	112 of 759 (14.8%)
Vaginal delivery	1056 of 1136 (93.0%)
Institutional delivery	1006 of 1120 (89.8%)
Trial arm	
IYCF	620 of 1169 (53.0%)
Non-IYCF	549 of 1169 (47.0%)
WASH	502 of 1169 (42.9%)
Non-WASH	667 of 1169 (57.1%)

1 Values are *n*, *n* (%), mean ± SD, or median [IQR]. Baseline variables are presented for mothers who had live births. Maternal and household data were collected ∼2 wk after consent was recorded. Baseline for infants was at birth. Hb, hemoglobin; IYCF, infant and young child feeding; WASH, water, sanitation, and hygiene.

### Trends in linear growth velocity

As expected, absolute growth velocity [∆ length (cm/mo)] decelerated among boys and girls between birth and 18 mo (**Supplemental Figure 1**). Relative growth velocity (∆ LAZ/mo) also decreased over this period as stunting prevalence increased (Supplemental Figure 1). For example, absolute mean growth velocity declined among boys from 2.90 cm/mo (95% CI: 2.81, 3.01 cm/mo) between 1 and 3 mo to 0.94 cm/mo (95% CI: 0.86, 1.02 cm/mo) between 12 and 18 mo; and among girls from 2.88 cm/mo (95% CI: 2.77, 2.98 cm/mo) between 1 and 3 mo to 0.90 cm/mo (95% CI: 0.82, 0.98 cm/mo) between 12 and 18 mo.

### Multivariable regression

**FIGURE 2:**
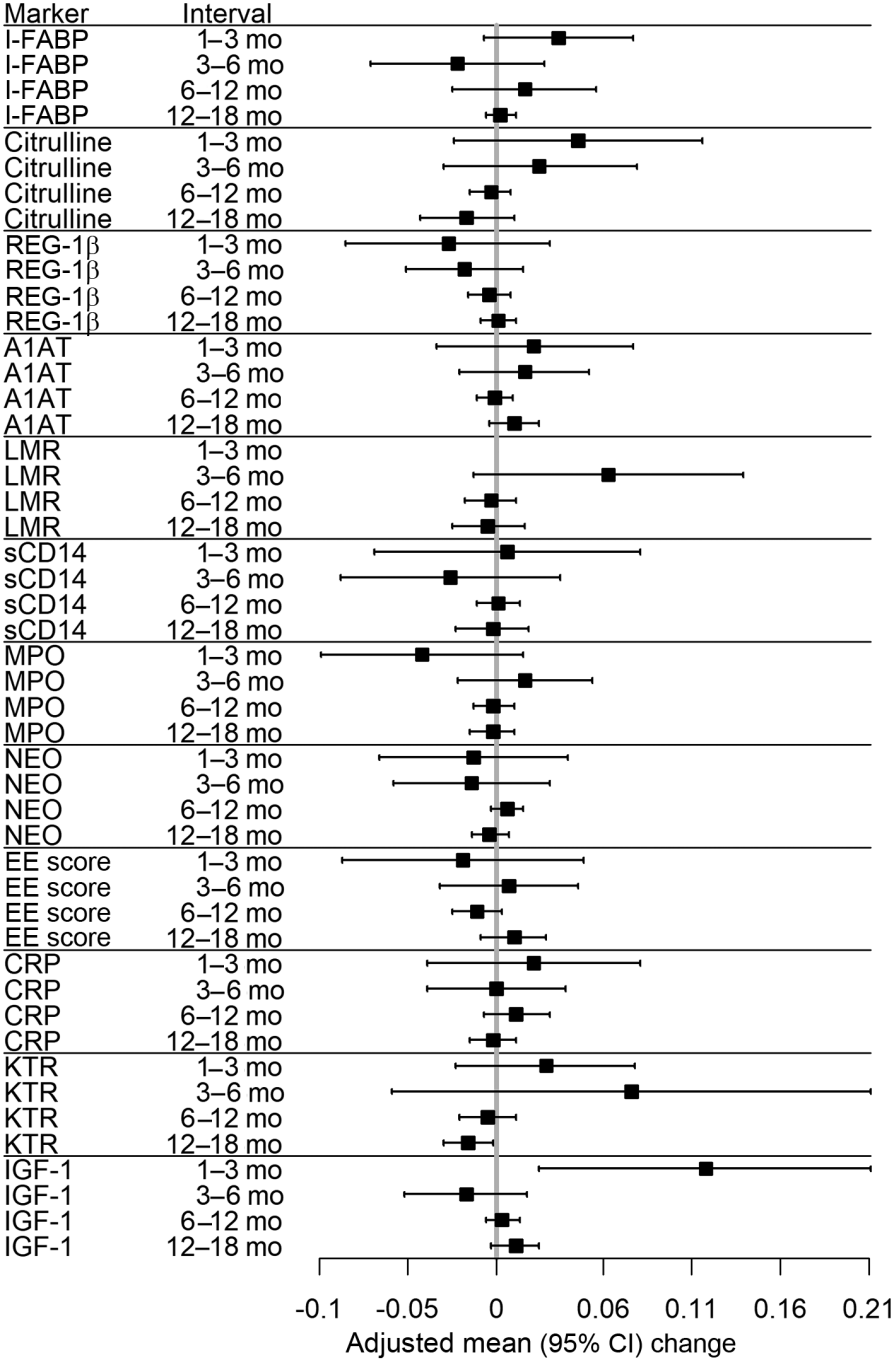
Adjusted mean change in LAZ (SD/mo) per 1-SD increase in biomarker concentration at the start of each follow-up interval. The mean (95% CI) LAZs at 1, 3, 6, 12, and 18 mo of age were −0.84 (−0.93, −0.75); −0.85 (−0.92, −0.77); −0.84 (−0.91, −0.76); −1.13 (−1.20, −1.07); and −1.42 (−1.48, −1.36), respectively. A1AT, fecal α-1 antitrypsin; CRP, plasma C-reactive protein; EE score, environmental enteropathy score; I-FABP, plasma intestinal fatty acid binding protein; IGF-1, insulin-like growth factor 1; KTR, kynurenine-to-tryptophan ratio; LAZ, length-for-age *z* score; LMR, lactulose-to-mannitol ratio (urinary); MPO, fecal myeloperoxidase; NEO, fecal neopterin; REG-1β, fecal regenerating gene 1β; sCD14, plasma soluble CD14.

The adjusted and unadjusted mean (95% CI) LAZ velocities (∆ LAZ/mo) associated with a 1-SD log increase in each biomarker at the start of each of the 4 age intervals are tabulated in **Supplemental Table 2** and adjusted values are shown in **Figure 2**. [See **Supplemental Table 3** for mean (95% CI), geometric mean (95% CI), and median [IQR] child age, LAZ, length, WHZ, and weight; and see **Supplemental Table 4** for mean (95% CI), geometric mean (95% CI), and median [IQR] concentrations of biomarkers at each time point.] **Supplemental Table 5** tabulates sensitivity analyses of the same associations, conducted to determine robustness to outlier biomarker values. In fully adjusted models, there were no associations between LAZ velocity and any indicator of changes characteristic of EED for intestinal structure [plasma intestinal fatty acid binding protein (I-FABP) and plasma citrulline]; intestinal regeneration [fecal regenerating gene 1β (REG-1β)]; microbial translocation (plasma sCD14); or intestinal inflammation (fecal myeloperoxidase and fecal neopterin) during any of the 4 age intervals investigated (Figure 2, Supplemental Table 2). Increased urinary mannitol fractional excretion was associated with a small increase in mean (95% CI) LAZ velocity during the 6–12 mo interval (0.013 LAZ/mo; 0.001, 0.025 LAZ/mo) (Supplemental Table 2), but this association was neither robust to outliers (Supplemental Table 5) nor associated with LAZ velocity during other age intervals. There were no associations between other indicators of intestinal permeability (A1AT, LMR, and lactulose excretion fraction) and LAZ velocity during any age interval. Increased plasma kynurenine-to-tryptophan ratio (KTR) was associated with a small decrease in mean (95% CI) LAZ velocity between 12 and 18 mo (−0.015 LAZ/mo; −0.029, −0.001 LAZ/mo) but this association was neither robust to outliers nor observed during other age intervals. Other indicators of systemic inflammation (plasma C-reactive protein, kynurenine, and tryptophan) were not associated with LAZ velocity during any age interval. Plasma IGF-1 measured at 1 mo of age was associated with a mean (95% CI) increase in LAZ velocity during the 1–3 mo interval only (0.118; 0.024, 0.211 LAZ/mo) and was robust to outliers. The EE score was not associated with LAZ velocity during any age interval.

In a sensitivity analysis which excluded infants who were already stunted at the start of each age interval, mean (95% CI) LAZ velocity was positively associated with plasma IGF-1 during 2 intervals (1–3 mo: 0.076; 0.016, 0.136 LAZ/mo; *P* = 0.013 and 12–18 mo: 0.020; 0.004, 0.035 LAZ/mo) and negatively associated with plasma KTR between 12 and 18 mo (−0.019; −0.037, −0.001 LAZ/mo) (**Supplemental Table 6**). Among these nonstunted infants, increased mean (95% CI) plasma I-FABP was associated with LAZ velocity during 2 age intervals, but in opposite directions: (+0.053; 0.013, 0.094 LAZ/mo during the 1–3 mo interval; and −0.009; 95% CI: −0.017, −0.001 LAZ/mo during the 6–12 mo interval). No other significant associations between biomarkers and LAZ velocity were observed among nonstunted infants. In investigating potential effect modification by infant sex, gut inflammation (fecal myeloperoxidase) or permeability (fecal A1AT) may have had a greater adverse effect on LAZ velocity among girls (**Supplemental Table 7**). The IYCF treatment group did not consistently modify the association between biomarkers and LAZ velocity (**Supplemental Table 8**).

When growth velocity was defined as change in absolute length per month, plasma IGF-1 was associated with significant mean (95% CI) increases in growth velocity during 2 age intervals (1–3 mo: 0.246; 0.052, 0.440 cm/mo and 12–18 mo: 0.039; 0.002, 0.077 cm/mo) and plasma KTR was associated with reduced growth velocity between 12 and 18 mo (−0.046; −0.087, −0.005 cm/mo) (**Table 3**) (58). No other associations between biomarkers and change in length per month were observed.

**TABLE 3:** Mean change in length (cm/mo) during 4 age intervals per 1-SD increase in biomarker concentration at the start of the interval^1^

	Minimally adjusted^2^			Fully adjusted^3^		
Interval	*n*	β (95% CI)	*P* value	*n*	β (95% CI)	*P* value
Intestinal fatty acid binding protein						
1–3 mo	557	0.109 (0.014, 0.204)	0.024	521	0.070 (−0.021, 0.162)	0.132
3–6 mo	693	−0.041 (−0.145, 0.063)	0.437	658	−0.051 (−0.155, 0.053)	0.338
6–12 mo	797	0.025 (−0.089, 0.139)	0.668	698	0.021 (−0.091, 0.134)	0.713
12–18 mo	882	−0.021 (−0.057, 0.016)	0.265	762	0.005 (−0.018, 0.029)	0.650
Citrulline						
1–3 mo	529	0.102 (−0.048, 0.252)	0.184	496	0.095 (−0.051, 0.241)	0.201
3–6 mo	658	0.048 (−0.055, 0.150)	0.363	657	0.049 (−0.075, 0.172)	0.440
6–12 mo	745	−0.001 (−0.030, 0.027)	0.931	683	−0.012 (−0.039, 0.015)	0.377
12–18 mo	824	−0.024 (−0.085, 0.037)	0.437	791	−0.050 (−0.126, 0.027)	0.204
Regenerating protein 1-β						
1–3 mo	369	−0.031 (−0.157, 0.096)	0.634	354	−0.065 (−0.181, 0.051)	0.272
3–6 mo	438	−0.078 (−0.143, −0.012)	0.020	433	−0.049 (−0.120, 0.021)	0.168
6–12 mo	722	−0.016 (−0.046, 0.013)	0.274	683	−0.013 (−0.042, 0.016)	0.388
12–18 mo	869	−0.027 (−0.067, 0.014)	0.201	755	0.005 (−0.022, 0.033)	0.696
A1AT						
1–3 mo	397	−0.008 (−0.119, 0.103)	0.889	381	0.023 (−0.096, 0.142)	0.701
3–6 mo	472	0.023 (−0.061, 0.106)	0.594	467	0.029 (−0.052, 0.111)	0.477
6–12 mo	744	−0.003 (−0.025, 0.020)	0.818	704	−0.004 (−0.027, 0.019)	0.732
12–18 mo	883	0.013 (−0.026, 0.053)	0.511	767	0.031 (−0.008, 0.070)	0.115
Lactulose:mannitol ratio						
3–6 mo	404	0.191 (0.036, 0.346)	0.016	386	0.140 (−0.032, 0.312)	0.111
6–12 mo	509	−0.006 (−0.032, 0.020)	0.628	464	−0.009 (−0.050, 0.033)	0.683
12–18 mo	538	0.011 (−0.073, 0.095)	0.800	515	−0.015 (−0.068, 0.039)	0.589
Lactulose excretion fraction						
3–6 mo	407	−0.071 (−0.584, 0.442)	0.786	389	−0.060 (−0.527, 0.407)	0.802
6–12 mo	510	−0.001 (−0.044, 0.041)	0.947	465	−0.008 (−0.047, 0.031)	0.681
12–18 mo	557	−0.020 (−0.042, 0.001)	0.065	533	−0.036 (−0.078, 0.007)	0.101
Mannitol excretion fraction						
3–6 mo	407	−0.024 (−0.126, 0.078)	0.641	389	−0.010 (−0.101, 0.082)	0.838
6–12 mo	510	0.027 (0.001, 0.054)	0.044	465	0.029 (−0.008, 0.065)	0.124
12–18 mo	557	0.014 (−0.022, 0.051)	0.439	533	0.004 (−0.053, 0.060)	0.900
Soluble CD14						
1–3 mo	556	−0.045 (−0.189, 0.099)	0.540	520	0.026 (−0.129, 0.181)	0.741
3–6 mo	694	−0.065 (−0.181, 0.051)	0.270	693	−0.062 (−0.194, 0.070)	0.357
6–12 mo	797	0.002 (−0.027, 0.030)	0.917	698	−0.001 (−0.028, 0.027)	0.966
12–18 mo	882	0.002 (−0.042, 0.046)	0.934	762	−0.009 (−0.067, 0.049)	0.759
Myeloperoxidase						
1–3 mo	402	−0.084 (−0.211, 0.042)	0.192	385	−0.113 (−0.285, 0.058)	0.195
3–6 mo	480	0.018 (−0.070, 0.106)	0.687	450	0.031 (−0.052, 0.114)	0.461
6–12 mo	745	0.002 (−0.026, 0.030)	0.896	705	−0.003 (−0.030, 0.024)	0.837
12–18 mo	882	0.007 (−0.027, 0.042)	0.676	766	−0.002 (−0.035, 0.030)	0.885
Neopterin						
1–3 mo	394	−0.077 (−0.222, 0.068)	0.298	377	−0.043 (−0.146, 0.060)	0.414
3–6 mo	466	−0.046 (−0.139, 0.048)	0.336	461	−0.028 (−0.123, 0.068)	0.571
6–12 mo	737	0.018 (−0.008, 0.044)	0.183	656	0.017 (−0.004, 0.037)	0.114
12–18 mo	876	−0.027 (−0.060, 0.005)	0.100	761	−0.010 (−0.039, 0.018)	0.476
EE score						
1–3 mo	389	−0.063 (−0.205, 0.079)	0.385	373	−0.053 (−0.196, 0.090)	0.469
3–6 mo	460	−0.026 (−0.113, 0.060)	0.555	433	0.021 (−0.067, 0.108)	0.643
6–12 mo	736	−0.024 (−0.057, 0.009)	0.152	655	−0.026 (−0.059, 0.007)	0.128
12–18 mo	873	0.035 (0.001, 0.070)	0.046	760	0.033 (−0.019, 0.084)	0.219
C-reactive protein						
1–3 mo	556	0.028 (−0.170, 0.226)	0.780	520	0.039 (−0.099, 0.177)	0.576
3–6 mo	694	0.022 (−0.074, 0.119)	0.652	659	−0.003 (−0.092, 0.086)	0.944
6–12 mo	797	0.031 (−0.005, 0.068)	0.089	698	0.032 (−0.009, 0.073)	0.131
12–18 mo	882	−0.004 (−0.038, 0.030)	0.825	762	−0.005 (−0.041, 0.031)	0.786
Kynurenine:tryptophan ratio						
1–3 mo	473	0.059 (−0.060, 0.179)	0.329	445	0.062 (−0.044, 0.169)	0.251
3–6 mo	612	0.125 (−0.110, 0.361)	0.297	581	0.159 (−0.148, 0.466)	0.310
6–12 mo	725	−0.011 (−0.052, 0.030)	0.611	665	−0.012 (−0.052, 0.028)	0.567
12–18 mo	801	−0.031 (−0.057, −0.006)	0.016	757	−0.046 (−0.087, 0.005)	0.029
Kynurenine						
1–3 mo	529	0.053 (−0.064, 0.170)	0.376	496	0.042 (−0.065, 0.148)	0.445
3–6 mo	658	0.101 (−0.067, 0.268)	0.240	657	0.111 (−0.105, 0.327)	0.314
6–12 mo	745	−0.002 (−0.033, 0.029)	0.899	656	−0.013 (−0.044, 0.018)	0.406
12–18 mo	824	−0.016 (−0.075, 0.043)	0.597	791	−0.013 (−0.069, 0.043)	0.648
Tryptophan						
1–3 mo	529	−0.002 (−0.148, 0.144)	0.983	496	−0.018 (−0.163, 0.127)	0.807
3–6 mo	658	0.015 (−0.099, 0.129)	0.799	657	−0.005 (−0.113, 0.103)	0.926
6–12 mo	745	−0.002 (−0.030, 0.027)	0.910	656	−0.006 (−0.033, 0.022)	0.689
12–18 mo	824	−0.001 (−0.066, 0.063)	0.971	791	0.017 (−0.044, 0.078)	0.588
Insulin-like growth factor-1						
1–3 mo	554	0.230 (0.031, 0.428)	0.023	518	0.246 (0.052, 0.440)	0.013
3–6 mo	693	−0.003 (−0.076, 0.070)	0.937	658	−0.031 (−0.114, 0.052)	0.462
6–12 mo	797	0.031 (0.010, 0.053)	0.004	698	0.014 (−0.010, 0.037)	0.249
12–18 mo	882	0.034 (0.005, 0.062)	0.023	762	0.039 (0.002, 0.077)	0.041

1 Results from multivariable linear regression. See **Supplemental Table 16** for lists of the retained variables in each model. A1AT, α-1 antitrypsin; EE score, environmental enteropathy score.

2 Adjusted for infant age and sex at the beginning of the interval.

3 Adjusted for the most explanatory subset identified from a list of prespecified candidate covariates (see Supplemental Text 1) by the Akaike Information Criterion (58).

In further analyses, different definitions of biomarker exposure at the start of each age interval which have been reported in the literature were applied. When biomarker concentration was categorized by quartiles (**Supplemental Table 9**), LAZ velocity was significantly greater during the 12–18 mo interval for I-FABP concentration in the fourth than in the first quartile (0.035; 0.003, 0.066 LAZ/mo); during the 1–3 mo (0.191; 0.011, 0.370 LAZ/mo) and 3–6 mo (−0.102; −0.196, −0.008 LAZ/mo) intervals for IGF-1 concentration in the fourth than in the first quartile at the start of the interval; and during the 6–12 mo interval for mannitol concentration in the fourth than in the first quartile (0.051; 0.005, 0.097 LAZ/mo). When the second and third quartiles of biomarker concentration were collapsed (the IQR) and compared with the first quartile (**Supplemental Table 10**), LMR was associated with a reduction in LAZ (−0.049; −0.094, −0.004 LAZ/mo) during the 6–12 mo interval. Finally, when biomarker exposure was defined in 2 quantiles (**Supplemental Table 11**), LAZ velocity was associated with fecal A1AT during the 6–12 mo interval (−0.027; −0.051, −0.002 LAZ/mo) and in the opposite direction during the 12–18 mo interval (+0.043; 0.005, 0.081 LAZ/mo); with fecal MPO during the 6–12 mo interval (−0.027; −0.054, −0.001 LAZ/mo); and with IGF-1 during the 1–3 mo interval (0.164; 0.022, 0.305 LAZ/mo).

In further analyses implementing the analytic method used by the MAL-ED study in evaluating EED and attained growth at 2 y, we used ordinal logistic regression to estimate the cumulative OR of attaining the higher or highest compared with the lowest LAZ group (lowest: LAZ < −2, higher: −2 ≤ LAZ < −1, highest ≥ −1) at 18 mo for the highest compared with the lowest biomarker quartile: KTR was associated with increased odds of attaining the lowest LAZ, whereas REG1-β, IGF-1, and tryptophan were associated with reduced odds of attaining the lowest LAZ (**Supplemental Table 12**). Finally, in adapting the method used by the MAL-ED study for evaluating EED and attained growth at 5 y, the variable representing exposure to each biomarker was calculated by detrending each ln-transformed measurement of the biomarker for age and whether or not the child consumed any breast milk by regression, and then calculating the mean of the residuals from these models. We found 2 significant associations. Higher mean IGF-1 was associated with higher LAZ at 18 mo, and higher mean fecal REG-1β was associated with higher LAZ at 18 mo (**Supplemental Table 13**). No consistent associations were observed between indicators of EED and change in weight (**Supplemental Table 14**) or WHZ (**Supplemental Table 15**).

## Discussion

The hypothesis of the SHINE trial was that EED is common in settings of poor sanitation and hygiene and is a major underlying cause of stunting. We have previously reported that rural Zimbabwean infants had substantial derangements in gut structure and evidence of chronic intestinal inflammation, consistent with EED (62). In this article, we assessed the magnitude and strength of association between EED biomarkers and linear growth velocity in >1000 HIV-unexposed children. Although these analyses are observational, the longitudinal cohort design strengthens causal inference. We defined our outcome as the relative (∆ LAZ/mo) and absolute (∆ length/mo) growth velocity during 4 age intervals per log increase in each of 11 biomarkers assessed at the start of the interval. Using our prespecified analytic approach, we observed 3 statistically significant, but small associations: KTR at 12 mo was associated with a decrease in LAZ velocity between 12 and 18 mo, mannitol excretion was associated with an increase in LAZ velocity between 6 and 12 mo, and plasma IGF-1 was associated with an increase in LAZ velocity between 1 and 3 mo of age. Results were similar for absolute growth, except that IGF-1 was also associated with a small increase in absolute length during the 12–18 mo interval. We found no other associations between any EED biomarker and relative or absolute linear growth velocity during any age interval. Collectively, these data do not support the hypothesis that EED—as measured using current biomarkers—is an underlying cause of linear growth faltering.

Findings from previous studies investigating child growth and EED have reported highly heterogeneous results. A systematic review of EED and growth studies published between 2010 and early 2017 showed that for every biomarker reported, there were numerous studies that both did and did not provide evidence supporting an association with child growth (30). For example, LMR, the oldest and most commonly studied EED biomarker, was inversely associated with linear growth velocity in studies in The Gambia (20, 33), Malawi (63), Bangladesh (64), and Brazil (38), but not associated with attained linear growth in Malawi (65) or Burkina Faso (66), and not associated with attained LAZ quartile at 2 y in the MAL-ED study (53). In evaluating the association of LMR with attained height-for-age *z* score at 5 y of age in the MAL-ED study, none of the LMRs assessed during the first 2 y of life were individually associated, but the detrended mean of the LMRs across the first 2 y was (60). Moreover, a recent study demonstrated that, contrary to traditionally held assumptions, mannitol and lactulose are both absorbed through normal-regulated and pathologic-unrestricted pathways; this work challenges our traditional understanding that urinary mannitol reflects only normal absorption whereas urinary lactulose reflects only pathologic translocation, thereby questioning the underlying assumption of the test (67). Many factors contribute to this heterogeneity in the literature. First, there are substantial differences in defining the exposure and outcome variables, which we explored in our data. In general, analytic approaches that compare extremes of growth outcomes (i.e., normal compared with stunted or highest compared with lowest LAZ quartile) rather than ∆ LAZ across the entire distribution of growth, or that compare the extremes of biomarker concentration (i.e., first compared with fourth quartile) rather than the entire distribution of the biomarker, yield more statistically significant associations between biomarkers and growth. This suggests that biomarkers do capture some component of underlying pathology; however, the lack of association between the full distributions of biomarkers and growth indicates the biomarkers explain very little of the variability in linear growth velocity. In an analysis of the children in the Peru site of the MAL-ED study, Colston et al. (34) showed that fecal A1AT, myeloperoxidase, and neopterin concentrations were each significantly associated with LAZ during the first 30 mo of life, but these biomarker concentrations explained only 0.7%, 2.4%, and 0.2%, respectively, of the total variability in growth. Second, studies vary in their choice of biomarkers, assay kits, and analytical platforms, and, for the lactulose–mannitol test, the doses of sugars given and time periods of urine collection. Third, differences in the ages of children studied probably contribute substantially to the heterogeneity of these studies. We have previously reported that all the biomarkers assessed in SHINE were highly dynamic between 1 mo and 18 mo of age (42). The MAL-ED study has similarly reported that the associations between fecal A1AT or myeloperoxidase and LAZ were very dynamic over the first 3 y of life in Peru, such that the magnitude and direction of association between these biomarkers and LAZ varied by age (34). Fourth, variability in the length of the growth interval after biomarker measurement contributes to the heterogeneity of study findings. In the Peru and Tanzania MAL-ED sites, the association between plasma tryptophan concentration and subsequent growth varied in a U-shaped function with increasing length of age interval assessed: each log increase in plasma tryptophan was associated with +0.05 LAZ when the interval length was 1 mo or 10 mo, but +0.11 LAZ for a 6-mo interval length. Fifth, the association between EED biomarkers and growth may be modified by child sex, as we observed in a sensitivity analysis in this study; this may reflect sex differences in inflammatory and immune responses to infection (68). Sixth, during the first 2 y after birth, absolute growth velocity (∆ length/mo) rapidly decelerates among all children and, among children in LMICs, relative growth velocity (∆ LAZ/mo) declines as stunting prevalence increases. Moreover, the shape of this decline varies for different global regions: in South Asia, LAZ velocity begins decelerating soon after birth, whereas in Africa, this deceleration typically begins after 6 mo. Thus, because both biomarker exposure and growth outcomes are dynamic with age, the association between these factors also changes with age. Finally, child growth is dependent on a myriad of factors other than gut health which may differ across studies, and impede the ability to identify true associations between biomarkers and growth where they exist.

Two concepts remain well-established. First, EED is virtually ubiquitous among people living in resource-poor unsanitary living conditions in LMICs. In studies based on intestinal biopsies, rather than biomarkers, mostly conducted in the 1960s in Asia (69–72), Africa (73–76), and Central America (77, 78), the intestinal characteristics of EED were observed in virtually all asymptomatic adults, as well as infants and young children (74, 79, 80). Second, although we lack empirical evidence that EED causes stunting, there is evidence that EED increases the *risk* of stunting. Growth depends not only on sufficient dietary intake of macro- and micronutrients, but also on their absorption and utilization. EED reduces absorptive surface area, and reduces or eliminates microvilli where numerous digestive enzymes are produced and nutrient absorption primarily occurs. EED also reduces gut barrier function which enables microbial translocation and chronic immune activation, thereby repartitioning nutrients away from growth to synthesize acute-phase proteins, and to fuel an increased metabolic rate (81). These mechanisms are well known in other inflammatory gut diseases (82, 83) and in animal models (84). Although it is possible that biological (e.g., reduced metabolic rate) or behavioral (e.g., reduced energy expenditure or increased energy intake) compensation mechanisms may dampen the adverse effects of EED, it is unlikely these mechanisms could eliminate the effects of EED on growth. Thus, the absence of consistent associations of clinically important magnitude between EED biomarkers and growth probably reflects the poor sensitivity and specificity of the biomarkers to capture EED, other unmeasured factors besides EED which also affect these biomarkers, and their short half-life. In addition, growth is saltatory: infants have “bursts” of linear growth (85) followed by long periods of stasis; therefore, intermittent measurement of biomarkers may not capture underlying pathology in the subset of children experiencing growth spurts at that time.

SHINE implemented a WASH intervention to test the hypothesis that reducing enteropathogen exposure would ameliorate EED and enhance linear growth. However, because the WASH intervention did not reduce enteropathogen infection, the hypothesized causal chain of events from enteropathogen exposure through EED to linear growth was not affected by our interventions. The WASH Benefits trials conducted in Bangladesh and Kenya tested similar low-cost WASH interventions and also found limited or no impact on enteropathogen infection, EED biomarkers, and linear growth (86, 87). Consensus articles arising from these trials have called for “transformative WASH”—interventions which drastically reduce child exposure to environmental pathogens (88, 89). The 3 trials could not test the hypothesis that EED causes stunting because the WASH interventions did not reduce EED. This article further demonstrates that none of the 11 biomarkers measured among children enrolled in the SHINE trial were consistently associated with linear growth during the first 18 mo after birth: of our 64 main comparisons, only 3 were statistically significant at the 0.05 level, almost exactly what would be expected by chance when testing 64 independent truly null hypotheses.

Further studies are needed to determine whether EED is deleterious in settings of high pathogen burden, to develop new biomarkers with greater sensitivity and specificity to identify EED, and to evaluate preventive and therapeutic interventions. Recent (90) and ongoing (91) studies which correlate EED biomarkers and nutritional status to histologic features of intestinal biopsies obtained from malnourished children will be very valuable in this regard.

## Supplementary Material

nqaa416_Supplemental_File

## Data Availability

Data described in the article, code book, and analytic code will be made publicly and freely available without restriction at ClinEpi Data Repository, University of Pennsylvania.

## Notes

The SHINE (Sanitation Hygiene Infant Nutrition Efficacy) trial is funded by Bill & Melinda Gates Foundation grants OPP1021542 (to JHH) to Johns Hopkins Bloomberg School of Public Health and OPP1143707 (to JHH) to Zvitambo Institute for Maternal and Child Health Research, the UK Department for International Development (to JHH), Wellcome Trust grants 093768/Z/10/Z (to AJP) and 108065/Z/15/Z (to AJP), Swiss Agency for Development and Cooperation grant 8106727, and UNICEF grant PCA–2017–0002.

Supplemental Text 1, Supplemental Tables 1–16, and Supplemental Figure 1 are available from the “Supplementary data” link in the online posting of the article and from the same link in the online table of contents at https://academic.oup.com/ajcn/.

Abbreviations used: AIC, Akaike Information Criterion; A1AT, α-1 antitrypsin; EED, environmental enteric dysfunction; EE score, environmental enteropathy score; I-FABP, intestinal fatty acid binding protein; IGF-1, insulin-like growth factor 1; IYCF, infant and young child feeding; KTR, kynurenine-to-tryptophan ratio; LAZ, length-for-age *z* score; LMIC, low- and middle-income country; LMR, lactulose-to-mannitol ratio; MAL-ED, The Etiology, Risk Factors, and Interactions of Enteric Infections and Malnutrition and the Consequences for Child Health and Development Project; REG-1β, regenerating gene 1β; SHINE, Sanitation Hygiene Infant Nutrition Efficacy; VHW, village health worker; WASH, water, sanitation, and hygiene; WHZ, weight-for-height *z* score.
