# Supplementary material for: Biomarkers of environmental enteric dysfunction are not consistently associated with linear growth velocity in rural Zimbabwean infants
Source: Am J Clin Nutr. 2021 Mar 19;113(5):1185–98. doi: 10.1093/ajcn/nqaa416 (PMC8106752; doi:10.1093/ajcn/nqaa416)
Supplement: nqaa416_Supplemental_File [file nqaa416_supplemental_file.docx]

**Online Supporting Material**

**Mutasa K and Ntozini R, et al.**

**Biomarkers of environmental enteric dysfunction are not consistently associated with linear growth velocity in rural Zimbabwean infants**

|  | Page |
| --- | --- |
| **Supplementary Text:**  Pre-specified variables considered for inclusion in fully adjusted models predicting growth velocity | 2 |
| **Supplementary Table 1.** Baseline characteristics of HIV-negative mothers and their liveborn infants who were and were not enrolled in the EED sub-study, and who were enrolled and provided at least one biological specimen` (feces, plasma, or urine) at the start of each growth interval | 3 |
| **Supplementary Table 2.** Mean change in LAZ (sd/month) per 1 SD increase in biomarker concentration at the start of each follow-up interval | 5 |
| **Supplementary Table 3**. Mean, geometric mean and median child age, LAZ, length, WHZ, and weight at each follow-up visit. | 9 |
| **Supplementary Table 4.** Mean, geometric mean, and median biomarker concentrations at each follow-up visit | 10 |
| **Supplementary Table 5**. Mean change in LAZ (sd/month) per 1 SD increase in biomarker concentration at the start of each follow-up interval after 95% winsorization of biomarker concentrations | 13 |
| **Supplementary Table 6.**  Mean Change in LAZ (sd/month) per 1 sd increase in biomarker concentration at the start of each follow-up interval, among infants who were not stunted at the start of the interval | 16 |
| **Supplementary Table 7.** Interaction effects with infant sex of mean change in LAZ (sd/month) per 1 sd increase in biomarker concentration at the start of each follow-up interval | 19 |
| **Supplementary Table 8.** Interaction effects with SHINE IYCF Intervention (which began at 6 months of infant age) of mean change in LAZ (sd/month) per 1 sd increase in biomarker concentration at the start of each follow-up interval | 23 |
| **Supplementary Table 9** Mean change in LAZ (SD/month) per biomarker concentration quartile at the start of each follow up interval | 26 |
| **Supplementary Table 10.** Mean change in LAZ (SD/month) for biomarker concentration in the 1st and 4th quartiles and intra-quartile range at the start of each follow-up interval | 35 |
| **Supplementary Table 11.**  Mean change in LAZ (SD/month) for children by biomarker quantile | 42 |
| **Supplementary Table 12**. Cumulative odds ratio of attaining the higher or highest compared to the lowest LAZ group (lowest: LAZ<−2, higher: −2 ≤ LAZ < −1, highest ≥ −1) at 18 mo for the highest vs the lowest biomarker quartile | 46 |
| **Supplementary Table 13.** Mean change in LAZ at age 18 months per 1 unit increase in mean age and breastfeeding de-trended biomarker concentration | 48 |
| **Supplementary Table 14**. Mean change in WHZ (sd/month) per 1 sd increase in biomarker concentration at the start of each follow-up interval | 49 |
| **Supplementary Table 15.** Mean change in weight (kg/month) per 1 sd increase in biomarker concentration at the start of each follow-up interval | **52** |
| **Supplementary Table 16.** Candidate variables selected by best subset selection for our main model with LAZ velocity (sd/month) as the outcome (Table 3 in paper). | **55** |
| **Supplementary Figure 1**. LAZ velocity and length velocity over by infant age among girls and boys in SHINE | **58** |

**Supplementary Text: Pre-specified variables considered for inclusion in fully adjusted models predicting growth velocity**

Maternal Age (age_mother_years, y)

Maternal Height (mom_height, cm)

Maternal MUAC (mom_muac, cm)

Maternal Married (married, dummy)

Maternal Education (mom_educ, y)

Maternal Religion (religion, dummy)

Maternal Depression (EPDS <10 vs ≥10)

Maternal Decision-making autonomy (dma, continuous)

Maternal Gender Norms Attitudes (gna, continuous)

Maternal Social Support (pss, continuous)

Household Occupants (hh_size integer)

Household Wealth score (ses_score, continuous)

Household Food insecure (csi, integer)

Household Has latrine (Improve_latrine, dummy)

Household Drinking water from improved source (improved_water, dummy)

Maternal Ever booked for antenatal care

Place of Delivery (place_ofdelivery, dummy)

Delivery mode (delivery_mode, dummy)

Multiple fetus pregnancy

Delivery occurred during hungry season

Infant Birth weight (birthweight, Kg)

Infant Preterm (premature, dummy) (get updated variable)

Length of breastfeeding

Infant Consumes Minimally Diverse Diet (ch_mdds, dummy)

Infant LAZ at the start of the interval (sd, continous)

IYCF (dummy)

WASH (dummy)

WASH+IYCF (dummy)

**Supplementary Table 1.** Baseline characteristics of HIV-negative mothers and their liveborn infants who were and were not enrolled in the EED sub-study, and who were enrolled and provided at least one biological specimen (feces, plasma or urine) at the start of each growth interval analysed

| **Characterisitc** | **Not Enrolled in EED** |  | **Enrolled** | | | | |
| --- | --- | --- | --- | --- | --- | --- | --- |
|  |  |  | **Total** | **Provided at least 1 specimen at 1-month visit** | **Provided at least 1 specimen at 3-month visit** | **Provided at least 1 specimen at 6-month visit** | **Provided at least 1 specimen at 12-month visit** |
| Mothers, N | 2,784 |  | 1,153 | 712 | 843 | 956 | 940 |
| Infants, N | 2,820 |  | 1,169 | 720 | 853 | 969 | 951 |
| Household Characteristics |  |  |  |  |  |  |  |
| Median Number of Occupants [IQR] | 5[3,6] |  | 5[3,6] | 5[3,6] | 5[4,6] | 5[3,6] | 5[3,6] |
| Wealth Quintile: |  |  |  |  |  |  |  |
| 1 (lowest) | 477/2550(18.7%) |  | 199/1124(17.7%) | 121/695(17.4%) | 138/822(16.8%) | 156/929(16.8%) | 151/918(16.4%) |
| 2 | 484/2550(19.0%) |  | 229/1124(20.4%) | 143/695(20.6%) | 170/822(20.7%) | 187/929(20.1%) | 189/918(20.6%) |
| 3 | 507/2550(19.9%) |  | 237/1124(21.1%) | 145/695(20.9%) | 176/822(21.4%) | 198/929(21.3%) | 200/918(21.8%) |
| 4 | 548/2550(21.5%) |  | 232/1124(20.6%) | 152/695(21.9%) | 175/822(21.3%) | 200/929(21.5%) | 202/918(22.0%) |
| 5 (highest) | 534/2550(20.9%) |  | 227/1124(20.2%) | 134/695(19.3%) | 163/822(19.8%) | 188/929(20.2%) | 176/918(19.2%) |
| Improved latrine at household | 821/2517(32.6%) |  | 343/1100(31.2%) | 209/679(30.8%) | 252/806(31.3%) | 287/909(31.6%) | 273/898(30.4%) |
| Main source of household drinking water improved | 1631/2538(63.6%) |  | 690/1103(62.6%) | 445/682(65.2%) | 505/808(62.5%) | 569/911(62.5%) | 561/900(62.3%) |
| Diet quality and food security |  |  |  |  |  |  |  |
| Household has minimum Diet Diversity Score | 827/2234(37.0%) |  | 472/1003(47.1%) | 294/628(46.8%) | 342/738(46.3%) | 394/831(47.4%) | 392/821(47.7%) |
| Median Coping Strategies Index score [IQR] | 1[0,8] |  | 0[0,5] | 0.0[0.0,4.0] | 0.0[0.0,5.0] | 0.0[0.0,5.0] | 0.0[0.0,5.0] |
| Maternal characteristics |  |  |  |  |  |  |  |
| Mean age (SD), years | 25.3(6.5) |  | 26.5(6.7) | 26.8(6.6) | 26.8(6.6) | 26.9(6.6) | 27.0(6.6) |
| Mean height (SD), cm | 159.7(8.9) |  | 160.0(7.7) | 160.1(7.7) | 160.1(6.8) | 160.0(8.1) | 160.0(8.1) |
| Mean mid-upper-arm circumference (SD), cm | 26.3(3.0) |  | 26.7(3.3) | 26.9(3.3) | 26.8(3.3) | 26.8(3.3) | 26.7(3.3) |
| Mean years of schooling completed (SD) | 9.6(1.8) |  | 9.6(1.8) | 9.6(1.7) | 9.6(1.7) | 9.6(1.8) | 9.6(1.8) |
| Married | 2491/2602(95.7%) |  | 1070/1131(94.6%) | 666/705(94.5%) | 792/829(95.5%) | 897/944(95.0%) | 881/919(95.9%) |
| Religion |  |  |  |  |  |  |  |
| Apostolic | 1223/2623(46.6%) |  | 550/1138(48.3%) | 336/710(47.3%) | 389/835(46.6%) | 457/950(48.1%) | 447/925(48.3%) |
| Other Christian | 217/2623(8.3%) |  | 514/1138(45.2%) | 324/710(45.6%) | 395/835(47.3%) | 437/950(46.0%) | 421/925(45.5%) |
| Other | 220/2623(8.4%) |  | 74/1138(6.5%) | 50/710(7.0%) | 51/835(6.1%) | 56/950(5.9%) | 57/925(6.2%) |
| Ever booked antenatal care | 2624/2645(99.2%) |  | 1060/1068(99.3%) | 652/655(99.5%) | 771/776(99.4%) | 874/880(99.3%) | 863/869(99.3%) |
| Mom anemic at baseline (Hb <12 g/dL)^1^ | 370/2228 (16.6%) |  | 158/1013(15.6%) | 98/636(15.4%) | 111/757(14.7%) | 128/845(15.1%) | 128/831(15.4%) |
| Infant characteristics |  |  |  |  |  |  |  |
| Female sex | 1395/2807(49.7%) |  | 567/1167(48.6%) | 356/720(49.4%) | 422/853(49.5%) | 483/969(49.8%) | 471/951(49.5%) |
| Mean birthweight (SD), kg | 3.1(0.5) |  | 3.1(0.5) | 3.1(0.5) | 3.1(0.5) | 3.1(0.5) | 3.1(0.5) |
| Birthweight <2500 g | 236/2460(9.6%) |  | 89/1113(8.0%) | 58/698(8.3%) | 66/827(8.0%) | 75/941(8.0%) | 72/921(7.8%) |
| Preterm | 250/1408(17.8%) |  | 112/759(14.8%) | 64/471(13.6%) | 73/566(12.9%) | 93/644(14.4%) | 95/638(14.9%) |
| Vaginal delivery | 2335/2528(92.4%) |  | 1056/1136(93.0%) | 668/708(94.4%) | 783/836(93.7%) | 879/949(92.6%) | 869/932(93.2%) |
| Institutional delivery | 2202/2484(88.7%) |  | 1006/1120(89.8%) | 641/707(90.7%) | 744/827(90.0%) | 847/934(90.7%) | 831/919(90.4%) |
| Trial Arm |  |  |  |  |  |  |  |
| IYCF | 1413/2820(50.1%) |  | 620/1169(53.0%) | 375/720(52.1%) | 464/853(54.4%) | 532/969(54.9%) | 517/951(54.4%) |
| non-IYCF | 1407/2820(49.9%) |  | 549/1169(47.0%) | 345/720(47.9%) | 389/853(45.6%) | 437/969(45.1%) | 434/951(45.6%) |
| WASH | 1564/2820(55.5%) |  | 502/1169(42.9%) | 300/720(41.7%) | 347/853(40.7%) | 410/969(42.3%) | 413/951(43.4%) |
| non-WASH | 1256/2820(44.5%) |  | 667/1169(57.1%) | 420/720(58.3%) | 506/853(59.3%) | 559/969(57.7%) | 538/951(56.6%) |

^1^Altitude and gestational age adjusted plasma hemoglobin

Baseline variables are presented for mothers who had livebirths. Maternal and household data were collected about 2 weeks after consent was recorded. Baseline for infants was birth. Data are n or n (%), unless otherwise specified.

IQR, interquartile range; min, minutes; SD, standard deviation; cm, centimeters; kg, kilograms; g, grams; IYCF, infant and young child feeding;

WASH, water sanitation and hygiene; LAZ, length-for-age z-score; WHZ, weight-for-height z-score; HCZ, head circumference z-score

**Supplementary Table 2.** Mean Change in LAZ (sd/month) per 1 sd Increase in Biomarker Concentration at the Start of each Follow-up Interval

| **Intestinal fatty acid binding protein** | | | | | | | |
| --- | --- | --- | --- | --- | --- | --- | --- |
|  | Unadjusted | | |  | Adjusted | | |
| Interval | N | β (95%CI) | p-value |  | N | β (95%CI) | p-value |
| 1-3mo | 557 | 0.023(-0.020,0.066) | 0.295 |  | 521 | 0.035(-0.007,0.077) | 0.107 |
| 3-6mo | 693 | -0.025(-0.074,0.024) | 0.310 |  | 692 | -0.022(-0.071,0.027) | 0.381 |
| 6-12mo | 797 | 0.012(-0.032,0.057) | 0.588 |  | 680 | 0.016(-0.025,0.056) | 0.447 |
| 12-18mo | 882 | -0.006(-0.019,0.006) | 0.320 |  | 762 | 0.002(-0.006,0.011) | 0.598 |
| **Citrulline** | | | | | | | |
|  | Unadjusted | | |  | Adjusted | | |
| Interval | N | β (95%CI) | p-value |  | N | β (95%CI) | p-value |
| 1-3mo | 529 | 0.041(-0.031,0.113) | 0.266 |  | 496 | 0.046(-0.024,0.116) | 0.198 |
| 3-6mo | 658 | 0.027(-0.017,0.072) | 0.224 |  | 657 | 0.024(-0.030,0.079) | 0.386 |
| 6-12mo | 745 | 0.003(-0.009,0.015) | 0.663 |  | 683 | -0.003(-0.015,0.008) | 0.541 |
| 12-18mo | 824 | -0.006(-0.028,0.015) | 0.560 |  | 793 | -0.017(-0.043,0.010) | 0.222 |
| **Regenerating Protein 1-β** | | | | | | | |
|  | Unadjusted | | |  | Adjusted | | |
| Interval | N | β (95%CI) | p-value |  | N | β (95%CI) | p-value |
| 1-3mo | 394 | -0.013(-0.103,0.077) | 0.777 |  | 377 | -0.015(-0.064,0.034) | 0.548 |
| 3-6mo | 466 | -0.015(-0.060,0.030) | 0.510 |  | 461 | -0.014(-0.058,0.030) | 0.531 |
| 6-12mo | 737 | 0.007(-0.005,0.018) | 0.279 |  | 656 | 0.006(-0.003,0.015) | 0.174 |
| 12-18mo | 876 | -0.010(-0.022,0.001) | 0.086 |  | 761 | -0.004(-0.014,0.007) | 0.494 |
| **Alpha-1 Antitrypsin** | | | | | | | |
|  | Unadjusted | | |  | Adjusted | | |
| Interval | N | β (95%CI) | p-value |  | N | β (95%CI) | p-value |
| 1-3mo | 397 | 0.012(-0.039,0.064) | 0.640 |  | 380 | 0.021(-0.035,0.076) | 0.471 |
| 3-6mo | 472 | 0.009(-0.028,0.047) | 0.631 |  | 467 | 0.016(-0.021,0.052) | 0.403 |
| 6-12mo | 744 | -0.001(-0.011,0.009) | 0.833 |  | 704 | -0.001(-0.011,0.009) | 0.802 |
| 12-18mo | 883 | 0.004(-0.010,0.018) | 0.574 |  | 767 | 0.010(-0.004,0.024) | 0.159 |
| **Lactulose:mannitol Ratio** | | | | | | | |
|  | Unadjusted | | |  | Adjusted | | |
| Interval | N | β (95%CI) | p-value |  | N | β (95%CI) | p-value |
| 3-6mo | 404 | 0.087(0.019,0.155) | 0.013 |  | 386 | 0.063(-0.013,0.139) | 0.103 |
| 6-12mo | 509 | -0.003(-0.013,0.006) | 0.468 |  | 464 | -0.003(-0.018,0.011) | 0.636 |
| 12-18mo | 538 | 0.004(-0.029,0.038) | 0.799 |  | 515 | -0.005(-0.025,0.016) | 0.655 |
| **Lactulose Excretion Fraction** | | | | | | | |
|  | Unadjusted | | |  | Adjusted | | |
| Interval | N | β (95%CI) | p-value |  | N | β (95%CI) | p-value |
| 3-6mo | 407 | -0.027(-0.239,0.185) | 0.801 |  | 389 | -0.025(-0.231,0.181) | 0.814 |
| 6-12mo | 510 | -0.003(-0.023,0.017) | 0.796 |  | 465 | -0.004(-0.023,0.014) | 0.649 |
| 12-18mo | 557 | -0.008(-0.017,0.002) | 0.106 |  | 533 | -0.013(-0.027,0.002) | 0.084 |
| **Mannitol Excretion Fraction** | | | | | | | |
|  | Unadjusted | | |  | Adjusted | | |
| Interval | N | β (95%CI) | p-value |  | N | β (95%CI) | p-value |
| 3-6mo | 407 | -0.003(-0.047,0.041) | 0.899 |  | 389 | -0.000(-0.042,0.041) | 0.993 |
| 6-12mo | 510 | 0.012(-0.000,0.024) | 0.057 |  | 510 | 0.013(0.001,0.025) | 0.028 |
| 12-18mo | 557 | 0.007(-0.006,0.020) | 0.291 |  | 533 | 0.003(-0.016,0.023) | 0.751 |
| **soluble CD14** | | | | | | | |
|  | Unadjusted | | |  | Adjusted | | |
| Interval | N | β (95%CI) | p-value |  | N | β (95%CI) | p-value |
| 1-3mo | 556 | -0.001(-0.069,0.067) | 0.982 |  | 520 | 0.006(-0.069,0.081) | 0.879 |
| 3-6mo | 694 | -0.024(-0.078,0.030) | 0.384 |  | 693 | -0.026(-0.088,0.036) | 0.411 |
| 6-12mo | 797 | 0.003(-0.009,0.015) | 0.605 |  | 680 | 0.001(-0.011,0.013) | 0.835 |
| 12-18mo | 882 | 0.001(-0.015,0.017) | 0.881 |  | 762 | -0.002(-0.023,0.018) | 0.822 |
| **Myeloperoxidase** | | | | | | | |
|  | Unadjusted | | |  | Adjusted | | |
| Interval | N | β (95%CI) | p-value |  | N | β (95%CI) | p-value |
| 1-3mo | 402 | -0.026(-0.085,0.034) | 0.396 |  | 385 | -0.047(-0.131,0.038) | 0.277 |
| 3-6mo | 480 | 0.013(-0.026,0.053) | 0.505 |  | 450 | 0.016(-0.022,0.054) | 0.405 |
| 6-12mo | 745 | 0.000(-0.012,0.012) | 0.976 |  | 690 | -0.002(-0.013,0.010) | 0.787 |
| 12-18mo | 882 | 0.001(-0.011,0.014) | 0.818 |  | 766 | -0.002(-0.015,0.010) | 0.733 |
| **Neopterin** | | | | | | | |
|  | Unadjusted | | |  | Adjusted | | |
| Interval | N | β (95%CI) | p-value |  | N | β (95%CI) | p-value |
| 1-3mo | 394 | -0.013(-0.103,0.077) | 0.777 |  | 377 | -0.015(-0.064,0.034) | 0.548 |
| 3-6mo | 466 | -0.015(-0.060,0.030) | 0.510 |  | 461 | -0.014(-0.058,0.030) | 0.531 |
| 6-12mo | 737 | 0.007(-0.005,0.018) | 0.279 |  | 656 | 0.006(-0.003,0.015) | 0.174 |
| 12-18mo | 876 | -0.010(-0.022,0.001) | 0.086 |  | 761 | -0.004(-0.014,0.007) | 0.494 |
| **EE Score** | | | | | | | |
|  | Unadjusted | | |  | Adjusted | | |
| Interval | N | β (95%CI) | p-value |  | N | β (95%CI) | p-value |
| 1-3mo | 389 | -0.007(-0.075,0.061) | 0.836 |  | 373 | -0.024(-0.093,0.045) | 0.492 |
| 3-6mo | 460 | -0.007(-0.047,0.033) | 0.719 |  | 455 | 0.007(-0.032,0.046) | 0.731 |
| 6-12mo | 736 | -0.011(-0.026,0.003) | 0.110 |  | 655 | -0.011(-0.025,0.003) | 0.127 |
| 12-18mo | 873 | 0.010(-0.002,0.023) | 0.104 |  | 760 | 0.010(-0.009,0.028) | 0.299 |
| **C-reactive Protein** | | | | | | | |
|  | Unadjusted | | |  | Adjusted | | |
| Interval | N | β (95%CI) | p-value |  | N | β (95%CI) | p-value |
| 1-3mo | 556 | 0.023(-0.052,0.098) | 0.550 |  | 520 | 0.021(-0.039,0.081) | 0.497 |
| 3-6mo | 694 | 0.008(-0.037,0.053) | 0.720 |  | 693 | 0.000(-0.039,0.039) | 0.985 |
| 6-12mo | 797 | 0.013(-0.003,0.029) | 0.107 |  | 680 | 0.011(-0.007,0.030) | 0.216 |
| 12-18mo | 882 | -0.002(-0.014,0.011) | 0.767 |  | 762 | -0.002(-0.015,0.011) | 0.797 |
| **Kynurenine:Tryptophan Ratio** | | | | | | | |
|  | Unadjusted | | |  | Adjusted | | |
| Interval | N | β (95%CI) | p-value |  | N | β (95%CI) | p-value |
| 1-3mo | 473 | 0.020(-0.036,0.077) | 0.476 |  | 445 | 0.028(-0.023,0.078) | 0.287 |
| 3-6mo | 612 | 0.056(-0.055,0.166) | 0.322 |  | 611 | 0.076(-0.059,0.211) | 0.272 |
| 6-12mo | 725 | -0.003(-0.020,0.014) | 0.741 |  | 665 | -0.005(-0.021,0.011) | 0.542 |
| 12-18mo | 801 | -0.010(-0.019,-0.000) | 0.039 |  | 757 | -0.015(-0.029,-0.001) | 0.042 |
| **Kynurenine** | | | | | | | |
|  | Unadjusted | | |  | Adjusted | | |
| Interval | N | β (95%CI) | p-value |  | N | β (95%CI) | p-value |
| 1-3mo | 529 | 0.005(-0.050,0.060) | 0.860 |  | 496 | 0.016(-0.035,0.067) | 0.540 |
| 3-6mo | 658 | 0.040(-0.038,0.118) | 0.310 |  | 657 | 0.052(-0.048,0.152) | 0.305 |
| 6-12mo | 745 | -0.002(-0.015,0.011) | 0.766 |  | 639 | -0.007(-0.020,0.007) | 0.334 |
| 12-18mo | 824 | -0.005(-0.026,0.016) | 0.637 |  | 793 | -0.004(-0.023,0.015) | 0.683 |
| **Tryptophan** | | | | | | | |
|  | Unadjusted | | |  | Adjusted | | |
| Interval | N | β (95%CI) | p-value |  | N | β (95%CI) | p-value |
| 1-3mo | 529 | -0.012(-0.081,0.058) | 0.741 |  | 496 | -0.010(-0.080,0.059) | 0.770 |
| 3-6mo | 658 | 0.003(-0.049,0.055) | 0.912 |  | 657 | -0.005(-0.055,0.044) | 0.831 |
| 6-12mo | 745 | -0.004(-0.016,0.009) | 0.572 |  | 639 | -0.004(-0.016,0.008) | 0.536 |
| 12-18mo | 824 | -0.002(-0.024,0.021) | 0.868 |  | 793 | 0.005(-0.016,0.026) | 0.666 |
| **Insulin-like growth factor-1** | | | | | | | |
|  | Unadjusted | | |  | Adjusted | | |
| Interval | N | β (95%CI) | p-value |  | N | β (95%CI) | p-value |
| 1-3mo | 554 | 0.102(0.008,0.196) | 0.034 |  | 518 | 0.118(0.024,0.211) | 0.014 |
| 3-6mo | 693 | -0.006(-0.038,0.026) | 0.705 |  | 692 | -0.017(-0.052,0.017) | 0.320 |
| 6-12mo | 797 | 0.010(0.001,0.019) | 0.024 |  | 680 | 0.003(-0.006,0.013) | 0.484 |
| 12-18mo | 882 | 0.007(-0.003,0.018) | 0.175 |  | 762 | 0.011(-0.003,0.024) | 0.129 |

**Supplementary Table 3**. Mean, geometric mean and median child age, LAZ, length, WHZ, and weight at each follow-up visit.

| **Age** | | | |
| --- | --- | --- | --- |
|  | Mean (95%CI) | Geometric Mean (95%CI) | Median [IQR] |
| 1mo | 1.52(1.48,1.55) | 1.42(1.39,1.46) | 1.26[1.12,1.88] |
| 3mo | 3.71(3.66,3.76) | 3.64(3.60,3.69) | 3.44[3.21,4.03] |
| 6mo | 6.80(6.73,6.87) | 6.72(6.66,6.78) | 6.38[6.18,6.98] |
| 12mo | 12.43(12.38,12.48) | 12.41(12.37,12.45) | 12.26[12.07,12.46] |
| 18mo | 18.33(18.26,18.39) | 18.30(18.24,18.36) | 18.05[17.85,18.35] |
| **LAZ** | | | |
|  | Mean (95%CI) | Geometric Mean (95%CI) | Median [IQR] |
| 1mo | -0.84(-0.93,-0.75) |  | -0.79[-1.62,-0.03] |
| 3mo | -0.85(-0.92,-0.77) |  | -0.84[-1.58,-0.07] |
| 6mo | -0.84(-0.91,-0.76) |  | -0.87[-1.61,-0.07] |
| 12mo | -1.13(-1.20,-1.07) |  | -1.12[-1.87,-0.46] |
| 18mo | -1.42(-1.48,-1.36) |  | -1.41[-2.80,-0.74] |
| **Length (cm)** | | | |
|  | Mean (95%CI) | Geometric Mean (95%CI) | Median [IQR] |
| 1mo | 54.3(54.1,54.5) | 54.2(54.0,54.4) | 54.3[52.4,56.0] |
| 3mo | 60.4(60.2,60.6) | 60.4(60.2,60.5) | 60.3[58.5,62.3] |
| 6mo | 65.9(65.7,66.1) | 65.9(65.7,66.1) | 65.8[64.0,68.0] |
| 12mo | 72.4(72.3,72.6) | 72.4(72.3,72.6) | 72.4[70.6,74.2] |
| 18mo | 77.7(77.5,77.8) | 77.6(77.5,77.8) | 77.7[75.8,79.7] |
| **WHZ** | | | |
|  | Mean (95%CI) | Geometric Mean (95%CI) | Median [IQR] |
| 1mo | 0.59(0.48,0.70) |  | 0.68[-0.30,1.56] |
| 3mo | 0.50(0.41,0.58) |  | 0.54[-0.29,1.36] |
| 6mo | 0.30(0.22,0.38) |  | 0.28[-0.45,1.06] |
| 12mo | 0.07(0.00,0.14) |  | 0.06[-0.62,0.80] |
| 18mo | 0.05(-0.01,0.11) |  | 0.04[-0.62,0.72] |
| **Weight (Kg)** | | | |
|  | Mean (95%CI) | Geometric Mean (95%CI) | Median [IQR] |
| 1mo | 4.68(4.62,4.74) | 4.60(4.54,4.66) | 4.66[4.12,5.14] |
| 3mo | 6.33(6.27,6.39) | 6.26(6.20,6.32) | 6.30[5.70,6.96] |
| 6mo | 7.63(7.56,7.70) | 7.56(7.50,7.62) | 7.53[6.90,8.30] |
| 12mo | 8.93(8.86,9.00) | 8.86(8.79,8.93) | 8.84[8.20,9.59] |
| 18mo | 9.95(9.88,10.02) | 9.88(9.81,9.95) | 9.86[9.20,10.63] |

**Supplementary Table 4.** Mean, geometric mean, and median biomarker concentrations at each follow-up visit

| **Intestinal fatty acid binding protein (pg/mL)** | | | |
| --- | --- | --- | --- |
| Visit | Mean (95%CI) | GM (95%CI) | Median [IQR] |
| 1mo | 1408(1343,1474) | 1225(1178,1275) | 1209[875,1715] |
| 3mo | 902(868,93) | 797(768,826) | 815[591,1103] |
| 6mo | 1002(900,1103) | 804(773,836) | 797[574,1142] |
| 12mo | 1283(1208,1357) | 1058(1017,1101) | 1052[741,1513] |
| 18mo | 1457(1361,1554) | 1164(1120,1210) | 1133[797,1673] |
| **Citrulline (ng/mL)** | | | |
| 1mo | 2993(2905,3082) | 2803(2724,2884) | 2813[2222,3518] |
| 3mo | 2830(2750,2910) | 2649(2578,2721) | 2648[2147,3279] |
| 6mo | 3014(2933,3095) | 2795(2719,2873) | 2823[2217,3583] |
| 12mo | 3472(3380,3564) | 3194(3097,3293) | 3305[2604,4155] |
| **Regenerating protein - 1β (ng/mL)** | | | |
| 1mo | 41.3(34.1,48.6) | 15.3(13.5,17.4) | 9.6[4.4,47.0] |
| 3mo | 88.1(77.1,99.1) | 33.2(29.1,38.0) | 43.3[7.1,95.1] |
| 6mo | 169.1(158.5,179.8) | 94.8(86.5,103.9) | 122.4[54.4,248.4] |
| 12mo | 171.5(161.3,181.8) | 85.9(77.9,94.8) | 144.0[46.1,253.85] |
| 18mo | 160.5(147.9,173.2) | 48.4(42.9,54.6) | 75.6[4.4,275.7] |
| **Alpha-1 antiytrypsin (ng/mL)** | | | |
| 1mo | 1.22(1.04,1.40) | 0.32(0.32,0.45) | 0.35[0.17,0.98] |
| 3mo | 0.94(0.80,1.09) | 0.39(0.35,0.44) | 0.36[0.18,0.78] |
| 6mo | 0.57(0.51,0.63) | 0.35(0.33,0.38) | 0.34[0.19,0.64] |
| 12mo | 0.41(0.38,0.43) | 0.28(0.26,0.30) | 0.30[0.16,0.58] |
| 18mo | 0.31(0.29,0.32) | 0.16(0.15,0.18) | 0.23[0.11,0.42] |
| **Lactulose:mannitol ratio** | | | |
| 3mo | 0.39(0.32,0.47) | 0.18(0.16,0.20) | 0.14[0.06,0.53] |
| 6mo | 0.35(0.27,0.42) | 0.14(0.13,0.16) | 0.11[0.06,0.33] |
| 12mo | 0.45(0.29,0.62) | 0.16(0.15,0.18) | 0.13[0.06,0.42] |
| 18mo | 0.18(0.15,0.21) | 0.08(0.08,0.09) | 0.07[0.04,0.16] |
| **Lactulose excretion fraction (ng/mL)** | | | |
| 3mo | 9.6e-05(6e-05,1.3e-04) | 3e-05(2.6e-05,3.4e-05) | 2.8e-05[1.3e-05,6.3e-05] |
| 6mo | 5.6e-05(4.1e-05,7e-05) | 2.1e-05(1.8e-05,2.3e-05) | 2e-05[8.3e-06,4.8e-05] |
| 12mo | 6.8e-05(4.7e-05,8.8e-05) | 1.9e-05(1.7e-05,2.2e-05) | 1.8e-05[7.7e-06,5.2e-05] |
| 18mo | 4.8e-04(9.4e-05,8.7e-04) | 1e-04(9e-05,1.2e-04) | 1.2e-04[3.9e-05,2.9e-04] |
| **Mannitol excretion fraction (ng/mL)** | | | |
| 3mo | 6.7e-04(5.6e-04,7.9e-04) | 1.7e-04(1.4e-04,2e-04) | 2.4e-04[3.6e-05,6.9e-04] |
| 6mo | 5.8e-04(4.6e-04,6.9e-04) | 1.4e-04(1.2e-04,1.6e-04) | 1.8e-04[3.6e-05,5.3e-04] |
| 12mo | 4.5e-04(3.8e-04,5.3e-04) | 1.2e-04(1.1e-04,1.4e-04) | 1.4e-04[3.3e-05,4.7e-04] |
| 18mo | 3.7e-03(3.2e-03,4.2e-03) | 1.1e-03(9.8e-04,1.3e-03) | 1.4e-03[3.9e-04,4.5e-03] |
| **soluble CD14 (pg/mL)** | | | |
| 1mo | 854078(821896,886260) | 713089(674446,753946) | 847046[593644,1083158] |
| 3mo | 1085402(1050362,1120441) | 938140(896645,981555) | 1077838[774243,1371413] |
| 6mo | 1178868(1144139,1213596) | 1023317(982404,1065933) | 1184762[828112,1515986] |
| 12mo | 1316094(1278726,1353463) | 1148342(1102374,1196228) | 1294720[949003,1651854] |
| 18mo | 1430944(1401365,1460522) | 1352131(1323336,1381554) | 1378823[1110290,1684102] |
| **Myeloperoxidase (ng/mL)** | | | |
| 1mo | 9755(8649,10860) | 1834(1086,3096) | 5861[2777,11647] |
| 3mo | 13597(12221,14972) | 6110(4667,8000) | 8221[3962,18074] |
| 6mo | 9959(9347,10571) | 3736(2760,5057) | 7233[4166,12413] |
| 12mo | 5698(5225,6170) | 860(561,1318) | 3959[2107,7044] |
| 18mo | 3184(2923,3444) | 12.47(6.20,25.06) | 2063[967,4109] |
| **Neopterin (nmol/L)** | | | |
| 1mo | 1135(996,1274) | 751(669,844) | 927[560,1413] |
| 3mo | 1706(1593,1820) | 1272(1166,1388) | 1442[867,2187] |
| 6mo | 1832(1677,1987) | 1357(1281,1438) | 1419[961,2153] |
| 12mo | 1438(1284,1591) | 804(739,875) | 977[461,1730] |
| 18mo | 794(667,920) | 242(213,275) | 318[123,763] |
| **EE Score** | | | |
| 1mo | 6.60(6.41,6.80) |  | 7.00[5.00,8.00] |
| 3mo | 7.24(7.07,7.41) |  | 7.00[6.00,9.00] |
| 6mo | 6.98(6.86,7.11) |  | 7.00[6.00,8.00] |
| 12mo | 6.08(5.96,6.21) |  | 6.00[5.00,7.00] |
| 18mo | 4.70(4.575,4.83) |  | 5.00[3.00,6.00] |
| **C-reactive protein (ng/mL)** | | | |
| 1mo | 4.63(3.14,6.13) | 0.38(0.32,0.45) | 0.27[0.09,1.57] |
| 3mo | 6.28(5.13,7.44) | 0.91(0.79,1.05) | 0.64[0.19,4.04] |
| 6mo | 3.85(3.27,4.43) | 0.83(0.73,0.94) | 0.85[0.20,3.60] |
| 12mo | 4.55(3.87,5.23) | 0.93(0.82,1.06) | 0.85[0.24,3.79] |
| 18mo | 4.64(3.92,5.37) | 0.53(0.45,0.62) | 0.81[0.11,3.96] |
| **Kynurenine:tryptophan ratio** | | | |
| 1mo | 55.9(54.3,57.6) | 52.9(51.5,54.4) | 52.5[42.4,63.9] |
| 3mo | 47.6(46.2,49.0) | 44.7(43.6,45.9) | 43.5[36.0,53.7] |
| 6mo | 45.4(44.0,46.8) | 42.3(41.3,43.4) | 40.7[33.8,52.1] |
| 12mo | 47.7(46.2,49.3) | 44.1(42.9,45.3) | 42.5[34.0,54.5] |
| **Kynurenine (ng/mL)** | | | |
| 1mo | 791(770,812) | 745(723,766) | 763[601,950] |
| 3mo | 635(620,651) | 603(588,618) | 599[493,737] |
| 6mo | 528(516,539) | 497(484,511) | 512[424,615] |
| 12mo | 488(477,499) | 455(442,468) | 473[388,583] |
| **Tryptophan (ng/mL)** | | | |
| 1mo | 14570(14305,14836) | 14113(13820,14413) | 14757[12261,16820] |
| 3mo | 14058(13811,14305) | 13591(13316,13872) | 13895[11831,16475] |
| 6mo | 12516(12278,12754) | 11901(11613,12197) | 12431[10112,14807] |
| 12mo | 11279(11013,11544) | 10395(10072,10728) | 11197[8786,13898] |
| **Insulin-like growth factor-1 (ng/mL)** | | | |
| 1mo | 32.8(31.2,34.3) | 26.5(25.0,28.0) | 30.9[18.5,41.7] |
| 3mo | 27.2(25.8,28.5) | 22.4(21.4,23.5) | 24.3[16.3,33.7] |
| 6mo | 21.7(20.8,22.6) | 17.8(16.9,18.6) | 19.9[13.6,27.8] |
| 12mo | 20.7(19.9,21.5) | 17.2(16.4,17.9) | 19.2[12.6,27.6] |
| 18mo | 22.9(22.0,23.9) | 18.4(17.6,19.2) | 20.5[11.8,29.9] |

**Supplementary Table 5.** Mean change in LAZ (SD/month) per 1 SD increase in biomarker concentration at the start of each follow-up interval after 95% winsorization of biomarker concentrations

| **Intestinal fatty acid binding protein** | | | | | | | |
| --- | --- | --- | --- | --- | --- | --- | --- |
|  | Unadjusted | | |  | Adjusted | | |
| Interval | N | β (95%CI) | p-value |  | N | β (95%CI) | p-value |
| 1-3mo | 557 | 0.023(-0.036,0.083) | 0.445 |  | 493 | 0.044(-0.021,0.109) | 0.182 |
| 3-6mo | 693 | -0.029(-0.089,0.031) | 0.341 |  | 562 | -0.030(-0.104,0.045) | 0.439 |
| 6-12mo | 797 | -0.008(-0.049,0.032) | 0.692 |  | 677 | 0.002(-0.036,0.040) | 0.916 |
| 12-18mo | 882 | -0.009(-0.038,0.021) | 0.563 |  | 762 | 0.008(-0.012,0.028) | 0.414 |
| **Citrulline** | | | | | | | |
|  | Unadjusted | | |  | Adjusted | | |
| Interval | N | β (95%CI) | p-value |  | N | β (95%CI) | p-value |
| 1-3mo | 529 | 0.047(-0.035,0.129) | 0.266 |  | 490 | 0.046(-0.037,0.129) | 0.280 |
| 3-6mo | 658 | 0.023(-0.024,0.071) | 0.339 |  | 539 | 0.014(-0.060,0.089) | 0.704 |
| 6-12mo | 745 | 0.004(-0.010,0.019) | 0.579 |  | 636 | -0.005(-0.019,0.009) | 0.464 |
| 12-18mo | 824 | -0.008(-0.035,0.018) | 0.533 |  | 715 | -0.020(-0.057,0.017) | 0.292 |
| **Regenerating Protein 1-β** | | | | | | | |
|  | Unadjusted | | |  | Adjusted | | |
| Interval | N | β (95%CI) | p-value |  | N | β (95%CI) | p-value |
| 1-3mo | 369 | -0.040(-0.118,0.038) | 0.311 |  | 351 | -0.048(-0.138,0.043) | 0.303 |
| 3-6mo | 438 | -0.024(-0.058,0.010) | 0.163 |  | 345 | -0.028(-0.068,0.013) | 0.183 |
| 6-12mo | 722 | -0.007(-0.021,0.006) | 0.288 |  | 602 | -0.005(-0.020,0.009) | 0.458 |
| 12-18mo | 869 | -0.011(-0.027,0.005) | 0.167 |  | 755 | 0.001(-0.010,0.012) | 0.884 |
| **Alpha-1 Antitrypsin** | | | | | | | |
|  | Unadjusted | | |  | Adjusted | | |
| Interval | N | β (95%CI) | p-value |  | N | β (95%CI) | p-value |
| 1-3mo | 397 | 0.012(-0.045,0.070) | 0.679 |  | 378 | 0.019(-0.046,0.083) | 0.567 |
| 3-6mo | 472 | 0.012(-0.033,0.056) | 0.610 |  | 371 | 0.019(-0.025,0.063) | 0.402 |
| 6-12mo | 744 | -0.005(-0.023,0.012) | 0.539 |  | 629 | -0.006(-0.024,0.012) | 0.504 |
| 12-18mo | 883 | 0.006(-0.010,0.023) | 0.464 |  | 767 | 0.013(-0.003,0.028) | 0.109 |
| **Lactulose:mannitol Ratio** | | | | | | | |
|  | Unadjusted | | |  | Adjusted | | |
| Interval | N | β (95%CI) | p-value |  | N | β (95%CI) | p-value |
| 3-6mo | 404 | 0.087(0.019,0.155) | 0.013 |  | 328 | 0.005(-0.096,0.105) | 0.925 |
| 6-12mo | 509 | -0.003(-0.013,0.006) | 0.468 |  | 440 | 0.004(-0.036,0.043) | 0.863 |
| 12-18mo | 538 | 0.004(-0.029,0.038) | 0.799 |  | 515 | -0.005(-0.110,0.100) | 0.927 |
| **Lactulose Excretion Fraction** | | | | | | | |
|  | Unadjusted | | |  | Adjusted | | |
| Interval | N | β (95%CI) | p-value |  | N | β (95%CI) | p-value |
| 3-6mo | 404 | 0.055(-0.045,0.156) | 0.282 |  | 389 | -0.025(-0.231,0.181) | 0.814 |
| 6-12mo | 509 | -0.003(-0.038,0.031) | 0.845 |  | 465 | -0.004(-0.023,0.014) | 0.649 |
| 12-18mo | 538 | -0.001(-0.109,0.108) | 0.986 |  | 533 | -0.013(-0.027,0.002) | 0.084 |
| **Mannitol Excretion Fraction** | | | | | | | |
|  | Unadjusted | | |  | Adjusted | | |
| Interval | N | β (95%CI) | p-value |  | N | β (95%CI) | p-value |
| 3-6mo | 407 | -0.006(-0.071,0.059) | 0.855 |  | 331 | -0.005(-0.082,0.071) | 0.889 |
| 6-12mo | 510 | 0.018(-0.003,0.039) | 0.092 |  | 440 | 0.018(-0.004,0.041) | 0.114 |
| 12-18mo | 557 | 0.011(-0.014,0.035) | 0.405 |  | 533 | -0.002(-0.041,0.037) | 0.905 |
| **soluble CD14** | | | | | | | |
|  | Unadjusted | | |  | Adjusted | | |
| Interval | N | β (95%CI) | p-value |  | N | β (95%CI) | p-value |
| 1-3mo | 556 | 0.009(-0.065,0.083) | 0.812 |  | 457 | 0.016(-0.080,0.111) | 0.748 |
| 3-6mo | 694 | -0.026(-0.086,0.034) | 0.403 |  | 563 | -0.033(-0.111,0.045) | 0.413 |
| 6-12mo | 797 | 0.004(-0.009,0.016) | 0.572 |  | 677 | 0.001(-0.011,0.014) | 0.813 |
| 12-18mo | 882 | 0.002(-0.015,0.019) | 0.830 |  | 762 | -0.002(-0.023,0.019) | 0.857 |
| **Myeloperoxidase** | | | | | | | |
|  | Unadjusted | | |  | Adjusted | | |
| Interval | N | β (95%CI) | p-value |  | N | β (95%CI) | p-value |
| 1-3mo | 402 | -0.028(-0.116,0.060) | 0.533 |  | 382 | -0.043(-0.133,0.047) | 0.353 |
| 3-6mo | 480 | 0.013(-0.035,0.060) | 0.604 |  | 376 | 0.011(-0.042,0.065) | 0.676 |
| 6-12mo | 745 | 0.001(-0.012,0.014) | 0.891 |  | 630 | -0.002(-0.015,0.011) | 0.720 |
| 12-18mo | 882 | 0.008(-0.022,0.037) | 0.608 |  | 766 | -0.005(-0.025,0.016) | 0.671 |
| **Neopterin** | | | | | | | |
|  | Unadjusted | | |  | Adjusted | | |
| Interval | N | β (95%CI) | p-value |  | N | β (95%CI) | p-value |
| 1-3mo | 394 | -0.046(-0.197,0.105) | 0.547 |  | 374 | -0.117(-0.306,0.072) | 0.224 |
| 3-6mo | 466 | -0.002(-0.051,0.047) | 0.924 |  | 362 | 0.028(-0.028,0.084) | 0.330 |
| 6-12mo | 737 | 0.016(-0.011,0.043) | 0.233 |  | 624 | 0.010(-0.015,0.036) | 0.436 |
| 12-18mo | 876 | -0.025(-0.060,0.010) | 0.160 |  | 761 | -0.005(-0.034,0.024) | 0.744 |
| **EE Score** | | | | | | | |
|  | Unadjusted | | |  | Adjusted | | |
| Interval | N | β (95%CI) | p-value |  | N | β (95%CI) | p-value |
| 1-3mo | 389 | -0.006(-0.078,0.066) | 0.868 |  | 370 | -0.018(-0.093,0.057) | 0.636 |
| 3-6mo | 460 | -0.005(-0.047,0.037) | 0.818 |  | 390 | 0.017(-0.028,0.063) | 0.454 |
| 6-12mo | 736 | -0.011(-0.026,0.003) | 0.113 |  | 612 | -0.013(-0.027,0.002) | 0.092 |
| 12-18mo | 873 | 0.010(-0.003,0.023) | 0.115 |  | 760 | 0.010(-0.009,0.029) | 0.304 |
| **C-reactive Protein** | | | | | | | |
|  | Unadjusted | | |  | Adjusted | | |
| Interval | N | β (95%CI) | p-value |  | N | β (95%CI) | p-value |
| 1-3mo | 556 | -0.029(-0.195,0.136) | 0.728 |  | 457 | -0.032(-0.231,0.167) | 0.753 |
| 3-6mo | 694 | 0.002(-0.061,0.066) | 0.948 |  | 563 | 0.001(-0.073,0.074) | 0.987 |
| 6-12mo | 797 | 0.016(-0.005,0.038) | 0.128 |  | 677 | 0.012(-0.009,0.033) | 0.268 |
| 12-18mo | 882 | -0.001(-0.019,0.017) | 0.917 |  | 762 | 0.001(-0.016,0.018) | 0.897 |
| **Kynurenine:Tryptophan Ratio** | | | | | | | |
|  | Unadjusted | | |  | Adjusted | | |
| Interval | N | β (95%CI) | p-value |  | N | β (95%CI) | p-value |
| 1-3mo | 473 | 0.020(-0.041,0.081) | 0.520 |  | 439 | 0.033(-0.027,0.094) | 0.281 |
| 3-6mo | 612 | 0.065(-0.062,0.192) | 0.318 |  | 493 | 0.112(-0.086,0.310) | 0.267 |
| 6-12mo | 725 | -0.002(-0.019,0.015) | 0.846 |  | 618 | -0.006(-0.024,0.011) | 0.486 |
| 12-18mo | 801 | -0.009(-0.022,0.003) | 0.144 |  | 679 | -0.010(-0.026,0.006) | 0.204 |
| **Kynurenine** | | | | | | | |
|  | Unadjusted | | |  | Adjusted | | |
| Interval | N | β (95%CI) | p-value |  | N | β (95%CI) | p-value |
| 1-3mo | 529 | 0.009(-0.047,0.065) | 0.751 |  | 490 | 0.022(-0.032,0.075) | 0.427 |
| 3-6mo | 658 | 0.050(-0.043,0.142) | 0.292 |  | 533 | 0.091(-0.065,0.247) | 0.251 |
| 6-12mo | 745 | -0.001(-0.016,0.013) | 0.846 |  | 636 | -0.007(-0.022,0.008) | 0.362 |
| 12-18mo | 824 | -0.005(-0.030,0.020) | 0.693 |  | 700 | -0.004(-0.028,0.019) | 0.704 |
| **Tryptophan** | | | | | | | |
|  | Unadjusted | | |  | Adjusted | | |
| Interval | N | β (95%CI) | p-value |  | N | β (95%CI) | p-value |
| 1-3mo | 529 | -0.005(-0.075,0.064) | 0.881 |  | 490 | -0.004(-0.075,0.068) | 0.918 |
| 3-6mo | 658 | 0.002(-0.053,0.056) | 0.950 |  | 539 | -0.010(-0.075,0.055) | 0.759 |
| 6-12mo | 745 | -0.004(-0.016,0.009) | 0.547 |  | 636 | -0.005(-0.017,0.007) | 0.431 |
| 12-18mo | 824 | -0.002(-0.025,0.022) | 0.885 |  | 700 | -0.002(-0.021,0.017) | 0.830 |
| **Insulin-like growth factor-1** | | | | | | | |
|  | Unadjusted | | |  | Adjusted | | |
| Interval | N | β (95%CI) | p-value |  | N | β (95%CI) | p-value |
| 1-3mo | 554 | 0.098(-0.008,0.204) | 0.069 |  | 455 | 0.138(0.019,0.258) | 0.023 |
| 3-6mo | 693 | -0.013(-0.062,0.036) | 0.613 |  | 562 | -0.032(-0.084,0.020) | 0.222 |
| 6-12mo | 797 | 0.012(-0.001,0.025) | 0.076 |  | 677 | 0.004(-0.010,0.019) | 0.558 |
| 12-18mo | 882 | 0.006(-0.004,0.017) | 0.251 |  | 762 | 0.009(-0.004,0.022) | 0.192 |

**Supplementary Table 6.** Mean Change in LAZ (sd/month) per 1 sd Increase in Biomarker Concentration at the Start of each Follow-up Interval, among Infants who were NOT Stunted at the Start of each Follow-up Interval

| **Intestinal fatty acid binding protein** | | | | | | | |
| --- | --- | --- | --- | --- | --- | --- | --- |
|  | Unadjusted | | |  | Adjusted | | |
| Interval | N | β (95%CI) | p-value |  | N | β (95%CI) | p-value |
| 1-3mo | 479 | 0.048(0.005,0.091) | 0.029 |  | 443 | 0.053(0.013,0.094) | 0.010 |
| 3-6mo | 588 | -0.009(-0.063,0.045) | 0.742 |  | 514 | -0.017(-0.074,0.040) | 0.557 |
| 6-12mo | 669 | -0.007(-0.019,0.005) | 0.250 |  | 611 | -0.009(-0.017,-0.001) | 0.037 |
| 12-18mo | 699 | -0.010(-0.031,0.010) | 0.312 |  | 671 | -0.005(-0.020,0.011) | 0.572 |
| **Citrulline** | | | | | | | |
| Interval | Unadjusted | | |  | Adjusted | | |
|  | N | β (95%CI) | p-value |  | N | β (95%CI) | p-value |
| 1-3mo | 453 | 0.053(-0.017,0.122) | 0.139 |  | 418 | 0.048(-0.017,0.114) | 0.149 |
| 3-6mo | 558 | 0.049(0.003,0.095) | 0.039 |  | 485 | 0.040(-0.009,0.089) | 0.113 |
| 6-12mo | 626 | -0.000(-0.012,0.012) | 0.973 |  | 573 | -0.003(-0.014,0.008) | 0.634 |
| 12-18mo | 650 | -0.012(-0.036,0.013) | 0.343 |  | 623 | -0.017(-0.045,0.011) | 0.234 |
| **Regenerating Protein 1-β** | | | | | | | |
|  | Unadjusted | | |  | Adjusted | | |
| Interval | N | β (95%CI) | p-value |  | N | β (95%CI) | p-value |
| 1-3mo | 318 | 0.001(-0.066,0.068) | 0.977 |  | 296 | -0.002(-0.071,0.067) | 0.953 |
| 3-6mo | 374 | -0.014(-0.045,0.016) | 0.351 |  | 369 | -0.008(-0.042,0.026) | 0.648 |
| 6-12mo | 610 | -0.004(-0.016,0.007) | 0.448 |  | 540 | -0.004(-0.015,0.008) | 0.519 |
| 12-18mo | 687 | -0.015(-0.033,0.003) | 0.100 |  | 660 | -0.008(-0.022,0.006) | 0.284 |
| **Alpha-1 Antitrypsin** | | | | | | | |
|  | Unadjusted | | |  | Adjusted | | |
| Interval | N | β (95%CI) | p-value |  | N | β (95%CI) | p-value |
| 1-3mo | 347 | 0.015(-0.033,0.063) | 0.552 |  | 324 | 0.017(-0.034,0.069) | 0.510 |
| 3-6mo | 401 | 0.007(-0.029,0.043) | 0.705 |  | 396 | 0.014(-0.023,0.051) | 0.457 |
| 6-12mo | 629 | 0.001(-0.008,0.010) | 0.758 |  | 556 | 0.000(-0.012,0.013) | 0.959 |
| 12-18mo | 699 | 0.006(-0.010,0.023) | 0.449 |  | 666 | 0.009(-0.009,0.027) | 0.314 |
| **Lactulose:mannitol Ratio** | | | | | | | |
|  | Unadjusted | | |  | Adjusted | | |
| Interval | N | β (95%CI) | p-value |  | N | β (95%CI) | p-value |
| 3-6mo | 339 | 0.005(-0.038,0.048) | 0.820 |  | 325 | 0.003(-0.040,0.046) | 0.892 |
| 6-12mo | 423 | -0.001(-0.012,0.010) | 0.911 |  | 353 | 0.003(-0.015,0.021) | 0.761 |
| 12-18mo | 419 | -0.001(-0.016,0.013) | 0.869 |  | 398 | -0.004(-0.019,0.011) | 0.605 |
| **Lactulose Excretion Fraction** | | | | | | | |
|  | Unadjusted | | |  | Adjusted | | |
| Interval | N | β (95%CI) | p-value |  | N | β (95%CI) | p-value |
| 3-6mo | 342 | -0.063(-0.416,0.289) | 0.724 |  | 328 | -0.057(-0.402,0.288) | 0.747 |
| 6-12mo | 424 | 0.000(-0.022,0.022) | 0.988 |  | 354 | 0.002(-0.017,0.022) | 0.819 |
| 12-18mo | 436 | -0.006(-0.024,0.012) | 0.528 |  | 414 | -0.016(-0.044,0.012) | 0.269 |
| **Mannitol Excretion Fraction** | | | | | | | |
|  | Unadjusted | | |  | Adjusted | | |
| Interval | N | β (95%CI) | p-value |  | N | β (95%CI) | p-value |
| 3-6mo | 342 | -0.010(-0.061,0.041) | 0.706 |  | 328 | -0.008(-0.057,0.040) | 0.731 |
| 6-12mo | 424 | 0.016(0.003,0.029) | 0.019 |  | 380 | 0.017(-0.009,0.044) | 0.206 |
| 12-18mo | 436 | 0.006(-0.008,0.020) | 0.399 |  | 414 | 0.006(-0.014,0.025) | 0.572 |
| **soluble CD14** | | | | | | | |
|  | Unadjusted | | |  | Adjusted | | |
| Interval | N | β (95%CI) | p-value |  | N | β (95%CI) | p-value |
| 1-3mo | 478 | -0.011(-0.084,0.062) | 0.766 |  | 442 | 0.004(-0.080,0.089) | 0.924 |
| 3-6mo | 589 | -0.000(-0.042,0.042) | 0.998 |  | 515 | 0.009(-0.042,0.060) | 0.728 |
| 6-12mo | 669 | 0.006(-0.006,0.018) | 0.349 |  | 611 | 0.004(-0.007,0.016) | 0.460 |
| 12-18mo | 699 | -0.003(-0.020,0.014) | 0.727 |  | 680 | -0.008(-0.033,0.016) | 0.507 |
| **Myeloperoxidase** | | | | | | | |
|  | Unadjusted | | |  | Adjusted | | |
| Interval | N | β (95%CI) | p-value |  | N | β (95%CI) | p-value |
| 1-3mo | 350 | -0.048(-0.118,0.021) | 0.172 |  | 325 | -0.077(-0.157,0.003) | 0.060 |
| 3-6mo | 408 | 0.025(-0.013,0.064) | 0.197 |  | 384 | 0.027(-0.011,0.066) | 0.163 |
| 6-12mo | 630 | 0.001(-0.011,0.013) | 0.842 |  | 557 | -0.004(-0.016,0.008) | 0.513 |
| 12-18mo | 697 | 0.004(-0.010,0.017) | 0.596 |  | 668 | 0.008(-0.008,0.025) | 0.328 |
| **Neopterin** | | | | | | | |
|  | Unadjusted | | |  | Adjusted | | |
| Interval | N | β (95%CI) | p-value |  | N | β (95%CI) | p-value |
| 1-3mo | 343 | -0.007(-0.123,0.109) | 0.907 |  | 318 | -0.010(-0.074,0.054) | 0.756 |
| 3-6mo | 398 | -0.016(-0.063,0.031) | 0.512 |  | 393 | -0.014(-0.061,0.033) | 0.565 |
| 6-12mo | 623 | 0.007(-0.005,0.019) | 0.234 |  | 552 | 0.006(-0.003,0.016) | 0.172 |
| 12-18mo | 693 | -0.010(-0.024,0.004) | 0.162 |  | 652 | -0.004(-0.016,0.009) | 0.568 |
| **EE Score** | | | | | | | |
|  | Unadjusted | | |  | Adjusted | | |
| Interval | N | β (95%CI) | p-value |  | N | β (95%CI) | p-value |
| 1-3mo | 340 | -0.022(-0.088,0.045) | 0.520 |  | 317 | -0.036(-0.103,0.032) | 0.301 |
| 3-6mo | 392 | -0.001(-0.044,0.042) | 0.960 |  | 387 | 0.009(-0.033,0.052) | 0.666 |
| 6-12mo | 622 | -0.007(-0.020,0.006) | 0.320 |  | 551 | -0.008(-0.022,0.005) | 0.237 |
| 12-18mo | 691 | 0.016(0.003,0.029) | 0.019 |  | 650 | 0.020(-0.001,0.042) | 0.059 |
| **C-reactive Protein** | | | | | | | |
|  | Unadjusted | | |  | Adjusted | | |
| Interval | N | β (95%CI) | p-value |  | N | β (95%CI) | p-value |
| 1-3mo | 478 | 0.002(-0.181,0.186) | 0.980 |  | 442 | 0.009(-0.112,0.130) | 0.884 |
| 3-6mo | 589 | -0.011(-0.047,0.024) | 0.528 |  | 515 | -0.019(-0.059,0.020) | 0.334 |
| 6-12mo | 669 | 0.008(-0.001,0.017) | 0.079 |  | 611 | 0.007(-0.002,0.017) | 0.135 |
| 12-18mo | 699 | -0.009(-0.022,0.004) | 0.184 |  | 671 | -0.008(-0.021,0.005) | 0.241 |
| **Kynurenine:Tryptophan Ratio** | | | | | | | |
|  | Unadjusted | | |  | Adjusted | | |
| Interval | N | β (95%CI) | p-value |  | N | β (95%CI) | p-value |
| 1-3mo | 407 | 0.009(-0.054,0.071) | 0.786 |  | 382 | 0.000(-0.054,0.055) | 0.986 |
| 3-6mo | 523 | -0.002(-0.030,0.027) | 0.900 |  | 457 | 0.001(-0.027,0.028) | 0.967 |
| 6-12mo | 609 | 0.004(-0.015,0.023) | 0.686 |  | 557 | 0.002(-0.017,0.021) | 0.860 |
| 12-18mo | 635 | -0.011(-0.022,-0.001) | 0.031 |  | 609 | -0.019(-0.037,-0.001) | 0.041 |
| **Kynurenine** | | | | | | | |
|  | Unadjusted | | |  | Adjusted | | |
| Interval | N | β (95%CI) | p-value |  | N | β (95%CI) | p-value |
| 1-3mo | 453 | 0.009(-0.050,0.068) | 0.772 |  | 418 | 0.006(-0.048,0.061) | 0.819 |
| 3-6mo | 558 | 0.003(-0.031,0.038) | 0.854 |  | 485 | 0.015(-0.022,0.052) | 0.422 |
| 6-12mo | 626 | -0.000(-0.013,0.012) | 0.957 |  | 573 | -0.003(-0.016,0.010) | 0.665 |
| 12-18mo | 650 | -0.013(-0.036,0.011) | 0.290 |  | 623 | -0.009(-0.031,0.013) | 0.420 |
| **Tryptophan** | | | | | | | |
|  | Unadjusted | | |  | Adjusted | | |
| Interval | N | β (95%CI) | p-value |  | N | β (95%CI) | p-value |
| 1-3mo | 453 | 0.007(-0.061,0.075) | 0.843 |  | 418 | 0.008(-0.056,0.071) | 0.810 |
| 3-6mo | 558 | 0.023(-0.020,0.065) | 0.296 |  | 490 | 0.029(-0.015,0.072) | 0.201 |
| 6-12mo | 626 | -0.003(-0.014,0.009) | 0.640 |  | 573 | -0.003(-0.014,0.009) | 0.659 |
| 12-18mo | 650 | -0.004(-0.031,0.022) | 0.746 |  | 623 | 0.006(-0.019,0.030) | 0.647 |
| **Insulin-like growth factor-1** | | | | | | | |
|  | Unadjusted | | |  | Adjusted | | |
| Interval | N | β (95%CI) | p-value |  | N | β (95%CI) | p-value |
| 1-3mo | 476 | 0.058(-0.001,0.117) | 0.056 |  | 440 | 0.076(0.016,0.136) | 0.013 |
| 3-6mo | 588 | 0.016(-0.013,0.044) | 0.279 |  | 514 | 0.005(-0.023,0.034) | 0.717 |
| 6-12mo | 669 | 0.014(0.005,0.024) | 0.004 |  | 584 | 0.007(-0.003,0.018) | 0.180 |
| 12-18mo | 699 | 0.014(0.002,0.026) | 0.027 |  | 671 | 0.020(0.004,0.035) | 0.012 |

**Supplementary Table 7.** Interaction effects with infant sex of mean change in LAZ (sd/month) per 1 sd increase in biomarker concentration at the start of each follow-up interval.^1^

| **Intestinal fatty acid binding protein** | | | | | | |
| --- | --- | --- | --- | --- | --- | --- |
|  | Biomarker Effect | | |  | Sex-by-Biomarker Difference-in-Differences | |
| Interval | N | Adjusted β (95%CI) | p-value |  | Adjusted β (95%CI) | p-value |
| 1-3mo | 521 | 0.019(-0.045,0.084) | 0.555 |  | 0.030(-0.062,0.121) | 0.525 |
| 3-6mo | 692 | -0.044(-0.129,0.041) | 0.313 |  | 0.042(-0.056,0.140) | 0.401 |
| 6-12mo | 680 | 0.003(-0.042,0.048) | 0.898 |  | 0.014(-0.060,0.088) | 0.711 |
| 12-18mo | 762 | 0.004(-0.008,0.016) | 0.521 |  | -0.005(-0.035,0.026) | 0.762 |
| **Citrulline** | | | | | | |
|  | Biomarker Effect | | |  | Sex-by-Biomarker Difference-in-Differences | |
| Interval | N | Adjusted β (95%CI) | p-value |  | Adjusted β (95%CI) | p-value |
| 1-3mo | 496 | 0.025(-0.084,0.133) | 0.657 |  | 0.040(-0.110,0.189) | 0.603 |
| 3-6mo | 657 | 0.018(-0.046,0.081) | 0.583 |  | 0.013(-0.090,0.117) | 0.799 |
| 6-12mo | 639 | -0.004(-0.023,0.015) | 0.676 |  | -0.003(-0.027,0.022) | 0.830 |
| 12-18mo | 794 | -0.009(-0.032,0.014) | 0.451 |  | -0.016(-0.048,0.017) | 0.343 |
| **Regenerating Protein 1-β** | | | | | | |
|  | Biomarker Effect | | |  | Sex-by-Biomarker Difference-in-Differences | |
| Interval | N | Adjusted β (95%CI) | p-value |  | Adjusted β (95%CI) | p-value |
| 1-3mo | 365 | -0.044(-0.114,0.026) | 0.222 |  | 0.055(-0.087,0.197) | 0.448 |
| 3-6mo | 433 | -0.026(-0.077,0.025) | 0.318 |  | 0.016(-0.047,0.078) | 0.624 |
| 6-12mo | 642 | -0.007(-0.028,0.014) | 0.528 |  | 0.002(-0.024,0.028) | 0.862 |
| 12-18mo | 755 | 0.008(-0.008,0.024) | 0.321 |  | -0.017(-0.041,0.007) | 0.172 |
| **Alpha-1 Antitrypsin** | | | | | | |
|  | Biomarker Effect | | |  | Sex-by-Biomarker Difference-in-Differences | |
| Interval | N | Adjusted β (95%CI) | p-value |  | Adjusted β (95%CI) | p-value |
| 1-3mo | 392 | 0.035(-0.059,0.129) | 0.469 |  | -0.024(-0.129,0.081) | 0.654 |
| 3-6mo | 467 | 0.006(-0.043,0.055) | 0.810 |  | 0.023(-0.049,0.095) | 0.528 |
| 6-12mo | 704 | 0.004(-0.008,0.016) | 0.555 |  | -0.019(-0.040,0.003) | 0.085^2^ |
| 12-18mo | 767 | 0.008(-0.010,0.026) | 0.397 |  | 0.004(-0.017,0.026) | 0.687 |
| **Lactulose:mannitol Ratio** | | | | | | |
|  | Biomarker Effect | | |  | Sex-by-Biomarker Difference-in-Differences | |
| Interval | N | Adjusted β (95%CI) | p-value |  | Adjusted β (95%CI) | p-value |
| 3-6mo | 386 | 0.066(-0.069,0.202) | 0.338 |  | -0.005(-0.229,0.220) | 0.968 |
| 6-12mo | 464 | -0.001(-0.053,0.051) | 0.973 |  | -0.003(-0.057,0.052) | 0.916 |
| 12-18mo | 515 | -0.020(-0.055,0.015) | 0.262 |  | 0.017(-0.022,0.055) | 0.389 |
| **Lactulose Excretion Fraction** | | | | | | |
|  | Biomarker Effect | | |  | Sex-by-Biomarker Difference-in-Differences | |
| Interval | N | Adjusted β (95%CI) | p-value |  | Adjusted β (95%CI) | p-value |
| 3-6mo | 389 | 0.026(-0.040,0.091) | 0.442 |  | -0.136(-0.671,0.398) | 0.617 |
| 6-12mo | 465 | -0.019(-0.065,0.027) | 0.422 |  | 0.017(-0.034,0.068) | 0.504 |
| 12-18mo | 531 | -0.019(-0.068,0.031) | 0.457 |  | 0.006(-0.042,0.055) | 0.805 |
| **Mannitol Excretion Fraction** | | | | | | |
|  | Biomarker Effect | | |  | Sex-by-Biomarker Difference-in-Differences | |
| Interval | N | Adjusted β (95%CI) | p-value |  | Adjusted β (95%CI) | p-value |
| 3-6mo | 386 | 0.037(-0.010,0.084) | 0.124 |  | -0.117(-0.247,0.013) | 0.078^3^ |
| 6-12mo | 509 | 0.005(-0.009,0.019) | 0.507 |  | 0.018(-0.009,0.045) | 0.194 |
| 12-18mo | 516 | -0.003(-0.032,0.027) | 0.864 |  | 0.007(-0.028,0.041) | 0.698 |
| **soluble CD14** | | | | | | |
|  | Biomarker Effect | | |  | Sex-by-Biomarker Difference-in-Differences | |
| Interval | N | Adjusted β (95%CI) | p-value |  | Adjusted β (95%CI) | p-value |
| 1-3mo | 520 | -0.058(-0.162,0.047) | 0.278 |  | 0.116(-0.022,0.254) | 0.098^4^ |
| 3-6mo | 693 | -0.042(-0.159,0.074) | 0.478 |  | 0.032(-0.093,0.158) | 0.615 |
| 6-12mo | 680 | 0.007(-0.010,0.023) | 0.445 |  | -0.012(-0.036,0.012) | 0.317 |
| 12-18mo | 762 | 0.018(-0.006,0.043) | 0.144 |  | -0.038(-0.081,0.006) | 0.089^4^ |
| **Myeloperoxidase** | | | | | | |
|  | Biomarker Effect | | |  | Sex-by-Biomarker Difference-in-Differences | |
| Interval | N | Adjusted β (95%CI) | p-value |  | Adjusted β (95%CI) | p-value |
| 1-3mo | 398 | -0.023(-0.148,0.103) | 0.725 |  | -0.031(-0.172,0.110) | 0.671 |
| 3-6mo | 450 | -0.003(-0.069,0.063) | 0.928 |  | 0.035(-0.043,0.112) | 0.380 |
| 6-12mo | 690 | 0.007(-0.009,0.024) | 0.382 |  | -0.021(-0.043,0.001) | 0.067^5^ |
| 12-18mo | 766 | -0.001(-0.013,0.012) | 0.935 |  | -0.009(-0.037,0.019) | 0.531 |
| **Neopterin** | | | | | | |
|  | Biomarker Effect | | |  | Sex-by-Biomarker Difference-in-Differences | |
| Interval | N | Adjusted β (95%CI) | p-value |  | Adjusted β (95%CI) | p-value |
| 1-3mo | 389 | 0.040(-0.274,0.353) | 0.805 |  | -0.065(-0.396,0.266) | 0.701 |
| 3-6mo | 461 | -0.014(-0.094,0.066) | 0.731 |  | -0.000(-0.103,0.103) | 1.000 |
| 6-12mo | 656 | 0.002(-0.016,0.020) | 0.815 |  | 0.007(-0.016,0.030) | 0.540 |
| 12-18mo | 761 | 0.004(-0.023,0.031) | 0.776 |  | -0.009(-0.039,0.020) | 0.538 |
| **EE Score** | | | | | | |
|  | Biomarker Effect | | |  | Sex-by-Biomarker Difference-in-Differences | |
| Interval | N | Adjusted β (95%CI) | p-value |  | Adjusted β (95%CI) | p-value |
| 1-3mo | 384 | 0.037(-0.072,0.145) | 0.505 |  | -0.116(-0.242,0.010) | 0.070^6^ |
| 3-6mo | 455 | -0.009(-0.064,0.047) | 0.754 |  | 0.031(-0.044,0.106) | 0.420 |
| 6-12mo | 655 | -0.005(-0.026,0.017) | 0.684 |  | -0.014(-0.045,0.016) | 0.362 |
| 12-18mo | 760 | 0.015(-0.006,0.035) | 0.160 |  | -0.012(-0.037,0.014) | 0.358 |
| **C-reactive Protein** | | | | | | |
|  | Biomarker Effect | | |  | Sex-by-Biomarker Difference-in-Differences | |
| Interval | N | Adjusted β (95%CI) | p-value |  | Adjusted β (95%CI) | p-value |
| 1-3mo | 520 | -0.049(-0.227,0.128) | 0.585 |  | 0.090(-0.091,0.270) | 0.330 |
| 3-6mo | 693 | -0.010(-0.077,0.058) | 0.784 |  | 0.023(-0.054,0.101) | 0.555 |
| 6-12mo | 680 | 0.019(-0.022,0.059) | 0.368 |  | -0.013(-0.056,0.030) | 0.560 |
| 12-18mo | 762 | 0.012(-0.007,0.030) | 0.226 |  | -0.025(-0.048,-0.002) | 0.030^7^ |
| **Kynurenine:Tryptophan Ratio** | | | | | | |
|  | Biomarker Effect | | |  | Sex-by-Biomarker Difference-in-Differences | |
| Interval | N | Adjusted β (95%CI) | p-value |  | Adjusted β (95%CI) | p-value |
| 1-3mo | 445 | -0.042(-0.147,0.063) | 0.433 |  | 0.103(-0.014,0.221) | 0.085^8^ |
| 3-6mo | 611 | 0.170(-0.167,0.507) | 0.322 |  | -0.150(-0.473,0.173) | 0.363 |
| 6-12mo | 621 | -0.009(-0.025,0.008) | 0.312 |  | -0.001(-0.026,0.024) | 0.927 |
| 12-18mo | 772 | -0.008(-0.028,0.013) | 0.456 |  | -0.012(-0.037,0.012) | 0.315 |
| **Kynurenine** | | | | | | |
|  | Biomarker Effect | | |  | Sex-by-Biomarker Difference-in-Differences | |
| Interval | N | Adjusted β (95%CI) | p-value |  | Adjusted β (95%CI) | p-value |
| 1-3mo | 496 | 0.000(-0.065,0.065) | 0.999 |  | 0.030(-0.074,0.133) | 0.575 |
| 3-6mo | 657 | 0.080(-0.107,0.267) | 0.402 |  | -0.053(-0.230,0.125) | 0.562 |
| 6-12mo | 639 | -0.002(-0.022,0.019) | 0.879 |  | -0.010(-0.036,0.017) | 0.468 |
| 12-18mo | 794 | 0.007(-0.014,0.028) | 0.504 |  | -0.022(-0.057,0.013) | 0.213 |
| **Tryptophan** | | | | | | |
|  | Biomarker Effect | | |  | Sex-by-Biomarker Difference-in-Differences | |
| Interval | N | Adjusted β (95%CI) | p-value |  | Adjusted β (95%CI) | p-value |
| 1-3mo | 496 | 0.002(-0.101,0.106) | 0.967 |  | -0.023(-0.163,0.116) | 0.744 |
| 3-6mo | 657 | -0.059(-0.147,0.029) | 0.186 |  | 0.103(-0.000,0.206) | 0.051^9^ |
| 6-12mo | 639 | -0.002(-0.020,0.016) | 0.860 |  | -0.004(-0.029,0.020) | 0.719 |
| 12-18mo | 794 | 0.011(-0.011,0.034) | 0.327 |  | -0.013(-0.054,0.029) | 0.549 |
| **Insulin-like growth factor-1** | | | | | | |
|  | Biomarker Effect | | |  | Sex-by-Biomarker Difference-in-Differences | |
| Interval | N | Adjusted β (95%CI) | p-value |  | Adjusted β (95%CI) | p-value |
| 1-3mo | 518 | 0.152(0.014,0.290) | 0.030 |  | -0.081(-0.243,0.081) | 0.327 |
| 3-6mo | 692 | -0.008(-0.045,0.029) | 0.666 |  | -0.033(-0.103,0.037) | 0.357 |
| 6-12mo | 680 | 0.015(-0.005,0.036) | 0.144 |  | -0.017(-0.040,0.006) | 0.138 |
| 12-18mo | 762 | 0.017(-0.008,0.041) | 0.184 |  | -0.010(-0.042,0.023) | 0.567 |

^1^ These were exploratory analyses to identify potential effect modification by infant sex. Where p value of interaction term <0.1, the analyses are presented stratified by sex in a footnote.

^2^ Mean change in LAZ (sd/month) per 1 sd Increase in Alpha-1 Antitrypsin between 6-12 mo, stratified by sex

| Interval | Sex | N | Adjusted β (95%CI) | p-value |
| --- | --- | --- | --- | --- |
| 6-12mo | Male | 367 | 0.004(-0.006,0.015) | 0.428 |
| 6-12mo | Female | 337 | -0.018(-0.035,-0.001) | 0.041 |

^3^ Mean change in LAZ (sd/month) per 1 sd Increase in mannitol excretion between 3-6 mo, stratified by sex

| Interval | Sex | N | Adjusted β (95%CI) | p-value |
| --- | --- | --- | --- | --- |
| 3-6mo | Male | 192 | 0.031(-0.009,0.072) | 0.130 |
| 3-6mo | Female | 194 | -0.077(-0.180,0.025) | 0.138 |

^4^ Mean change LAZ (sd/month) per 1 sd increase in soluble CD14 between 1-3 mo and 12-18 mo, stratified by sex:

| Interval | Sex | N | Adjusted β (95%CI) | p-value |
| --- | --- | --- | --- | --- |
| 1-3mo | Male | 258 | -0.067(-0.170,0.037) | 0.206 |
| 1-3mo | Female | 262 | 0.059(-0.048,0.167) | 0.278 |
| 12-18mo | Male | 387 | 0.017(-0.006,0.041) | 0.153 |
| 12-18mo | Female | 375 | -0.022(-0.056,0.012) | 0.209 |

^5^ Mean change LAZ (sd/month) per 1 sd increase in Myeloperoxidase between 6-12 mo, stratified by sex:

| Interval | Sex | N | Adjusted β (95%CI) | p-value |
| --- | --- | --- | --- | --- |
| 6-12mo | Male | 361 | 0.008(-0.008,0.024) | 0.306 |
| 6-12mo | Female | 329 | -0.015(-0.030,0.000) | 0.054 |

^6^ Mean change in LAZ (sd/month) per 1 sd increase in EE Score between 1-3 mo, stratified by sex:

| Interval | Sex | N | Adjusted β (95%CI) | p-value |
| --- | --- | --- | --- | --- |
| 1-3mo | Male | 199 | 0.036(-0.073,0.145) | 0.518 |
| 1-3mo | Female | 185 | -0.080(-0.150,-0.010) | 0.026 |

^7^ Mean change in LAZ (sd/month) per 1 sd increase in CRP between 12-18 mo, stratified by sex:

| Interval | Sex | N | Adjusted β (95%CI) | p-value |
| --- | --- | --- | --- | --- |
| 12-18mo | Male | 387 | 0.012(-0.004,0.028) | 0.128 |
| 12-18mo | Female | 375 | -0.010(-0.023,0.002) | 0.105 |

^8^ Mean change LAZ (sd/month) per 1 sd increase in KTR between 1-3 mo, stratified by sex:

| Interval | Sex | N | Adjusted β (95%CI) | p-value |
| --- | --- | --- | --- | --- |
| 1-3mo | Male | 225 | -0.033(-0.141,0.075) | 0.551 |
| 1-3mo | Female | 220 | 0.062(0.011,0.114) | 0.017 |

^9^ Mean change in LAZ (sd/month) per 1 sd increase in Tryptophan between 3-6 mo, stratified by sex:

| Interval | Sex | N | Adjusted β (95%CI) | p-value |
| --- | --- | --- | --- | --- |
| 3-6mo | Male | 320 | -0.058(-0.142,0.026) | 0.176 |
| 3-6mo | Female | 337 | 0.037(-0.013,0.087) | 0.143 |

**Supplementary Table 8.** Interaction effects with SHINE IYCF Intervention (which began at 6 months of infant age) of mean change in LAZ (sd/month) per 1 sd increase in biomarker concentration at the start of each follow-up interval

| **Intestinal fatty acid binding protein** | | | | | | |
| --- | --- | --- | --- | --- | --- | --- |
|  | Biomarker Effect | | |  | IYCF-by-Biomarker Difference-in-Differences | |
| Interval | N | Adjusted β (95%CI) | p-value |  | Adjusted β (95%CI) | p-value |
| 6-12mo | 680 | 0.038(-0.005,0.080) | 0.081 |  | -0.023(-0.124,0.077) | 0.325 |
| 12-18mo | 762 | 0.006(-0.005,0.018) | 0.290 |  | -0.011(-0.034,0.011) | 0.274 |
| **Citrulline** | | | | | | |
|  | Biomarker Effect | | |  | IYCF-by-Biomarker Difference-in-Differences | |
| Interval | N | Adjusted β (95%CI) | p-value |  | Adjusted β (95%CI) | p-value |
| 6-12mo | 683 | -0.014(-0.036,0.009) | 0.235 |  | 0.016(-0.010,0.042) | 0.246 |
| 12-18mo | 794 | -0.028(-0.081,0.026) | 0.306 |  | 0.021(-0.032,0.074) | 0.414 |
| **Regenerating Protein 1-β** | | | | | | |
|  | Biomarker Effect | | |  | IYCF-by-Biomarker Difference-in-Differences | |
| Interval | N | Adjusted β (95%CI) | p-value |  | Adjusted β (95%CI) | p-value |
| 6-12mo | 642 | -0.006(-0.029,0.017) | 0.596 |  | 0.001(-0.027,0.029) | 0.935 |
| 12-18mo | 755 | -0.003(-0.018,0.013) | 0.738 |  | 0.007(-0.016,0.030) | 0.554 |
| **Alpha-1 Antitrypsin** | | | | | | |
|  | Biomarker Effect | | |  | IYCF-by-Biomarker Difference-in-Differences | |
| Interval | N | Adjusted β (95%CI) | p-value |  | Adjusted β (95%CI) | p-value |
| 6-12mo | 704 | -0.004(-0.018,0.011) | 0.638 |  | 0.005(-0.017,0.027) | 0.580 |
| 12-18mo | 767 | 0.013(-0.007,0.033) | 0.213 |  | -0.006(-0.028,0.017) | 0.633 |
| **Lactulose:mannitol Ratio** | | | | | | |
|  | Biomarker Effect | | |  | IYCF-by-Biomarker Difference-in-Differences | |
| Interval | N | Adjusted β (95%CI) | p-value |  | Adjusted β (95%CI) | p-value |
| 6-12mo | 456 | -0.004(-0.058,0.050) | 0.887 |  | -0.001(-0.059,0.057) | 0.980 |
| 12-18mo | 515 | -0.007(-0.046,0.031) | 0.704 |  | 0.003(-0.119,0.126) | 0.858 |
| **Lactulose Excretion Fraction** | | | | | | |
|  | Biomarker Effect | | |  | IYCF-by-Biomarker Difference-in-Differences | |
| Interval | N | Adjusted β (95%CI) | p-value |  | Adjusted β (95%CI) | p-value |
| 6-12mo | 465 | -0.006(-0.029,0.018) | 0.629 |  | 0.006(-0.034,0.047) | 0.742 |
| 12-18mo | 531 | -0.017(-0.046,0.012) | 0.244 |  | 0.013(-0.019,0.045) | 0.254 |
| **Mannitol Excretion Fraction** | | | | | | |
|  | Biomarker Effect | | |  | IYCF-by-Biomarker Difference-in-Differences | |
| Interval | N | Adjusted β (95%CI) | p-value |  | Adjusted β (95%CI) | p-value |
| 6-12mo | 509 | 0.013(-0.006,0.033) | 0.176 |  | 0.001(-0.025,0.027) | 0.948 |
| 12-18mo | 516 | -0.001(-0.037,0.036) | 0.964 |  | 0.005(-0.039,0.049) | 0.783 |
| **soluble CD14** | | | | | | |
|  | Biomarker Effect | | |  | IYCF-by-Biomarker Difference-in-Differences | |
| Interval | N | Adjusted β (95%CI) | p-value |  | Adjusted β (95%CI) | p-value |
| 6-12mo | 733 | -0.003(-0.021,0.015) | 0.772 |  | 0.009(-0.014,0.032) | 0.475 |
| 12-18mo | 762 | -0.003(-0.038,0.032) | 0.881 |  | 0.001(-0.034,0.035) | 0.971 |
| **Myeloperoxidase** | | | | | | |
|  | Biomarker Effect | | |  | IYCF-by-Biomarker Difference-in-Differences | |
| Interval | N | Adjusted β (95%CI) | p-value |  | Adjusted β (95%CI) | p-value |
| 6-12mo | 705 | -0.003(-0.019,0.013) | 0.736 |  | 0.005(-0.020,0.029) | 0.713 |
| 12-18mo | 766 | -0.014(-0.037,0.009) | 0.235 |  | 0.015(-0.009,0.040) | 0.185 |
| **Neopterin** | | | | | | |
|  | Biomarker Effect | | |  | IYCF-by-Biomarker Difference-in-Differences | |
| Interval | N | Adjusted β (95%CI) | p-value |  | Adjusted β (95%CI) | p-value |
| 6-12mo | 656 | 0.011(-0.010,0.033) | 0.306 |  | -0.006(-0.030,0.018) | 0.566 |
| 12-18mo | 761 | -0.014(-0.062,0.035) | 0.588 |  | 0.012(-0.040,0.064) | 0.575 |
| **EE Score** | | | | | | |
|  | Biomarker Effect | | |  | IYCF-by-Biomarker Difference-in-Differences | |
| Interval | N | Adjusted β (95%CI) | p-value |  | Adjusted β (95%CI) | p-value |
| 6-12mo | 655 | -0.011(-0.033,0.010) | 0.309 |  | 0.000(-0.028,0.029) | 0.971 |
| 12-18mo | 760 | 0.013(-0.016,0.043) | 0.370 |  | -0.007(-0.037,0.024) | 0.622 |
| **C-reactive Protein** | | | | | | |
|  | Biomarker Effect | | |  | IYCF-by-Biomarker Difference-in-Differences | |
| Interval | N | Adjusted β (95%CI) | p-value |  | Adjusted β (95%CI) | p-value |
| 6-12mo | 733 | 0.008(-0.014,0.031) | 0.453 |  | 0.011(-0.023,0.045) | 0.493 |
| 12-18mo | 762 | -0.012(-0.033,0.009) | 0.259 |  | 0.019(-0.004,0.043) | 0.080^1^ |
| **Kynurenine:Tryptophan Ratio** | | | | | | |
|  | Biomarker Effect | | |  | IYCF-by-Biomarker Difference-in-Differences | |
| Interval | N | Adjusted β (95%CI) | p-value |  | Adjusted β (95%CI) | p-value |
| 6-12mo | 621 | -0.007(-0.024,0.010) | 0.438 |  | -0.005(-0.029,0.020) | 0.656 |
| 12-18mo | 772 | -0.002(-0.018,0.013) | 0.761 |  | -0.023(-0.052,0.006) | 0.106 |
| **Kynurenine** | | | | | | |
|  | Biomarker Effect | | |  | IYCF-by-Biomarker Difference-in-Differences | |
| Interval | N | Adjusted β (95%CI) | p-value |  | Adjusted β (95%CI) | p-value |
| 6-12mo | 639 | -0.007(-0.026,0.013) | 0.500 |  | 0.000(-0.027,0.027) | 0.995 |
| 12-18mo | 794 | -0.012(-0.052,0.029) | 0.568 |  | 0.015(-0.031,0.061) | 0.497 |
| **Tryptophan** | | | | | | |
|  | Biomarker Effect | | |  | IYCF-by-Biomarker Difference-in-Differences | |
| Interval | N | Adjusted β (95%CI) | p-value |  | Adjusted β (95%CI) | p-value |
| 6-12mo | 639 | -0.015(-0.033,0.003) | 0.103 |  | 0.021(-0.003,0.046) | 0.063^2^ |
| 12-18mo | 794 | -0.015(-0.055,0.026) | 0.478 |  | 0.038(-0.020,0.096) | 0.181 |
| **Insulin-like growth factor-1** | | | | | | |
|  | Biomarker Effect | | |  | IYCF-by-Biomarker Difference-in-Differences | |
| Interval | N | Adjusted β (95%CI) | p-value |  | Adjusted β (95%CI) | p-value |
| 6-12mo | 680 | -0.002(-0.020,0.017) | 0.860 |  | 0.007(-0.016,0.030) | 0.496 |
| 12-18mo | 762 | 0.013(-0.010,0.036) | 0.257 |  | -0.005(-0.033,0.022) | 0.664 |

^1^These were exploratory analyses to identify potential effect modification by treatment group (IYCF or non-IYCF). Where p value of interaction term <0.1, the analyses are presented stratified by sex in a footnote.

^2^ Mean change in LAZ (sd/month) per 1 sd Increase in CRP between 12-18 mo, stratified by treatment group (IYCF or non-IYCF):

| Interval | Group | N | Adjusted β (95%CI) | p-value |
| --- | --- | --- | --- | --- |
| 12-18mo | non-IYCF | 352 | -0.011(-0.030,0.008) | 0.249 |
| 12-18mo | IYCF | 410 | 0.008(-0.002,0.019) | 0.130 |

^3^ Mean change in LAZ (sd/month) per 1 sd Increase in tryptophan between 12-18 mo, stratified by treatment group (IYCF or non-IYCF):

| Interval | Group | N | Adjusted β (95%CI) | p-value |
| --- | --- | --- | --- | --- |
| 6-12mo | non-IYCF | 280 | -0.015(-0.032,0.002) | 0.093 |
| 6-12mo | IYCF | 359 | 0.005(-0.010,0.020) | 0.530 |

**Supplementary Table 9.** Mean change in LAZ (SD/month) per biomarker concentration quartile at the start of each follow up interval.

|  |  | **Intestinal fatty acid binding protein** | | | | | | | | |
| --- | --- | --- | --- | --- | --- | --- | --- | --- | --- | --- |
|  |  | Unadjusted | | | | |  | Adjusted | | |
| Interval | Quartile | N | β (95%CI) | | | p-value |  | N | β (95%CI) | p-value |
| 1-3mo | q1 | 66 | ref | | | ref |  | 521 | ref | ref |
| 1-3mo | q2 | 114 | -0.064(-0.263,0.135) | | | 0.53 |  | 521 | -0.029(-0.214,0.157) | 0.7631 |
| 1-3mo | q3 | 174 | -0.082(-0.306,0.143) | | | 0.4746 |  | 521 | -0.046(-0.281,0.189) | 0.6999 |
| 1-3mo | q4 | 203 | -0.018(-0.203,0.168) | | | 0.8529 |  | 521 | 0.048(-0.137,0.232) | 0.6142 |
| 3-6mo | q1 | 256 | ref | | | ref |  | 692 | ref | ref |
| 3-6mo | q2 | 217 | 0.041(-0.106,0.188) | | | 0.5874 |  | 692 | 0.060(-0.107,0.227) | 0.4817 |
| 3-6mo | q3 | 155 | 0.061(-0.021,0.143) | | | 0.1442 |  | 692 | 0.039(-0.040,0.119) | 0.3291 |
| 3-6mo | q4 | 65 | -0.119(-0.319,0.082) | | | 0.2456 |  | 692 | -0.081(-0.287,0.126) | 0.4446 |
| 6-12mo | q1 | 315 | ref | | | ref |  | 680 | ref | ref |
| 6-12mo | q2 | 211 | 0.001(-0.033,0.035) | | | 0.9667 |  | 680 | 0.001(-0.034,0.036) | 0.9535 |
| 6-12mo | q3 | 159 | -0.017(-0.051,0.017) | | | 0.325 |  | 680 | -0.013(-0.047,0.021) | 0.4596 |
| 6-12mo | q4 | 112 | 0.012(-0.026,0.050) | | | 0.5381 |  | 680 | 0.024(-0.014,0.061) | 0.2165 |
| 12-18mo | q1 | 176 | ref | | | ref |  | 762 | ref | ref |
| 12-18mo | q2 | 216 | 0.005(-0.074,0.084) | | | 0.8984 |  | 762 | 0.036(-0.028,0.099) | 0.2705 |
| 12-18mo | q3 | 232 | 0.012(-0.048,0.072) | | | 0.6959 |  | 762 | 0.034(-0.005,0.072) | 0.085 |
| 12-18mo | q4 | 258 | -0.002(-0.059,0.055) | | | 0.9532 |  | 762 | 0.035(0.003,0.066) | 0.0309 |
|  |  | **Regenerating protein 1-β** | | | | | | | | |
|  |  | Unadjusted | | | | |  | Adjusted | | |
| Interval | Quartile | N | β (95%CI) | | | p-value |  | N | β (95%CI) | p-value |
| 1-3mo | q1 | 186 | ref | | | ref |  | 365 | ref | ref |
| 1-3mo | q2 | 129 | -0.110(-0.281,0.061) | | | 0.207 |  | 365 | -0.127(-0.290,0.035) | 0.124 |
| 1-3mo | q3 | 38 | -0.050(-0.254,0.155) | | | 0.6335 |  | 365 | -0.055(-0.270,0.159) | 0.6119 |
| 1-3mo | q4 | 16 | -0.125(-0.377,0.128) | | | 0.3321 |  | 365 | -0.104(-0.363,0.156) | 0.4337 |
| 3-6mo | q1 | 126 | ref | | | ref |  | 433 | ref | ref |
| 3-6mo | q2 | 170 | -0.003(-0.106,0.099) | | | 0.9485 |  | 433 | 0.016(-0.080,0.113) | 0.7396 |
| 3-6mo | q3 | 89 | -0.008(-0.128,0.111) | | | 0.8927 |  | 433 | -0.032(-0.147,0.083) | 0.5816 |
| 3-6mo | q4 | 53 | -0.029(-0.147,0.090) | | | 0.6376 |  | 433 | -0.024(-0.139,0.091) | 0.6804 |
| 6-12mo | q1 | 73 | ref | | | ref |  | 642 | ref | ref |
| 6-12mo | q2 | 178 | 0.016(-0.033,0.064) | | | 0.5272 |  | 642 | 0.012(-0.037,0.061) | 0.6232 |
| 6-12mo | q3 | 248 | -0.003(-0.049,0.043) | | | 0.8951 |  | 642 | -0.001(-0.046,0.045) | 0.9797 |
| 6-12mo | q4 | 223 | -0.010(-0.054,0.034) | | | 0.6575 |  | 642 | -0.010(-0.054,0.033) | 0.6447 |
| 12-18mo | q1 | 133 | ref | | | ref |  | 755 | ref | ref |
| 12-18mo | q2 | 170 | 0.043(-0.018,0.105) | | | 0.1696 |  | 755 | 0.028(-0.022,0.077) | 0.2772 |
| 12-18mo | q3 | 289 | 0.031(-0.018,0.081) | | | 0.219 |  | 755 | 0.036(-0.032,0.104) | 0.2983 |
| 12-18mo | q4 | 277 | -0.002(-0.031,0.026) | | | 0.8689 |  | 755 | 0.014(-0.034,0.062) | 0.5675 |
|  |  | **Citrulline** | | | | | | | | |
|  |  | Unadjusted | | | | |  | Adjusted | | |
| Interval | Quartile | N | β (95%CI) | | | p-value |  | N | β (95%CI) | p-value |
| 1-3mo | q1 | 144 | ref | | | ref |  | 496 | ref | ref |
| 1-3mo | q2 | 142 | 0.174(-0.017,0.366) | | | 0.0745 |  | 496 | 0.124(-0.071,0.320) | 0.2126 |
| 1-3mo | q3 | 138 | 0.085(-0.103,0.273) | | | 0.3765 |  | 496 | 0.055(-0.128,0.239) | 0.5534 |
| 1-3mo | q4 | 105 | 0.168(-0.042,0.378) | | | 0.1167 |  | 496 | 0.185(-0.022,0.393) | 0.0801 |
| 3-6mo | q1 | 200 | ref | | | ref |  | 657 | ref | ref |
| 3-6mo | q2 | 200 | 0.036(-0.133,0.204) | | | 0.6767 |  | 657 | 0.020(-0.147,0.188) | 0.8117 |
| 3-6mo | q3 | 158 | 0.038(-0.054,0.130) | | | 0.4199 |  | 657 | 0.026(-0.065,0.117) | 0.5765 |
| 3-6mo | q4 | 100 | 0.068(-0.047,0.183) | | | 0.2475 |  | 657 | 0.040(-0.091,0.171) | 0.554 |
| 6-12mo | q1 | 209 | ref | | | ref |  | 683 | ref | ref |
| 6-12mo | q2 | 188 | 0.017(-0.021,0.055) | | | 0.3897 |  | 683 | 0.004(-0.032,0.039) | 0.8361 |
| 6-12mo | q3 | 184 | 0.013(-0.020,0.046) | | | 0.4302 |  | 683 | -0.008(-0.039,0.023) | 0.629 |
| 6-12mo | q4 | 164 | 0.011(-0.028,0.050) | | | 0.5896 |  | 683 | -0.006(-0.042,0.030) | 0.7362 |
| 12-18mo | q1 | 133 | ref | | | ref |  | 715 | ref | ref |
| 12-18mo | q2 | 163 | -0.043(-0.133,0.047) | | | 0.3474 |  | 715 | -0.041(-0.125,0.042) | 0.3334 |
| 12-18mo | q3 | 212 | -0.030(-0.131,0.070) | | | 0.5538 |  | 715 | -0.062(-0.151,0.027) | 0.1749 |
| 12-18mo | q4 | 316 | -0.047(-0.137,0.043) | | | 0.3099 |  | 715 | -0.071(-0.181,0.040) | 0.2085 |
|  |  | **Alpha-1 Antitrypsin** | | | | | | | | |
|  |  | Unadjusted | | | | |  | Adjusted | | |
| Interval | Quartile | N | β (95%CI) | | | p-value |  | N | β (95%CI) | p-value |
| 1-3mo | q1 | 78 | ref | | | ref |  | 392 | ref | ref |
| 1-3mo | q2 | 97 | -0.122(-0.343,0.098) | | | 0.2756 |  | 392 | -0.111(-0.316,0.094) | 0.2883 |
| 1-3mo | q3 | 70 | 0.013(-0.293,0.318) | | | 0.9353 |  | 392 | 0.018(-0.271,0.308) | 0.9005 |
| 1-3mo | q4 | 152 | -0.065(-0.250,0.119) | | | 0.4888 |  | 392 | -0.075(-0.261,0.112) | 0.4341 |
| 3-6mo | q1 | 87 | ref | | | ref |  | 467 | ref | ref |
| 3-6mo | q2 | 120 | 0.094(-0.026,0.214) | | | 0.124 |  | 467 | 0.093(-0.013,0.199) | 0.0867 |
| 3-6mo | q3 | 109 | 0.046(-0.075,0.166) | | | 0.4566 |  | 467 | 0.044(-0.064,0.152) | 0.4269 |
| 3-6mo | q4 | 156 | 0.056(-0.071,0.184) | | | 0.3843 |  | 467 | 0.082(-0.032,0.197) | 0.1589 |
| 6-12mo | q1 | 142 | ref | | | ref |  | 661 | ref | ref |
| 6-12mo | q2 | 181 | 0.000(-0.038,0.039) | | | 0.9849 |  | 661 | 0.012(-0.028,0.052) | 0.5609 |
| 6-12mo | q3 | 218 | -0.017(-0.053,0.019) | | | 0.3564 |  | 661 | -0.008(-0.046,0.031) | 0.6915 |
| 6-12mo | q4 | 203 | -0.033(-0.070,0.004) | | | 0.0845 |  | 661 | -0.020(-0.059,0.020) | 0.3277 |
| 12-18mo | q1 | 218 | ref | | | ref |  | 767 | ref | ref |
| 12-18mo | q2 | 224 | -0.019(-0.067,0.030) | | | 0.4516 |  | 767 | 0.002(-0.031,0.035) | 0.8932 |
| 12-18mo | q3 | 233 | 0.042(-0.030,0.114) | | | 0.249 |  | 767 | 0.064(0.002,0.126) | 0.0421 |
| 12-18mo | q4 | 208 | 0.002(-0.047,0.050) | | | 0.9395 |  | 767 | 0.021(-0.011,0.054) | 0.1956 |
|  |  | **Lactulose:mannitol ratio** | | | | | | | | |
|  |  | Unadjusted | | | | |  | Adjusted | | |
| Interval | Quartile | N | β (95%CI) | | | p-value |  | N | β (95%CI) | p-value |
| 3-6mo | q1 | 78 | ref | | | ref |  | 386 | ref | ref |
| 3-6mo | q2 | 99 | -0.028(-0.150,0.094) | | | 0.6531 |  | 386 | -0.039(-0.154,0.076) | 0.5061 |
| 3-6mo | q3 | 90 | -0.106(-0.224,0.013) | | | 0.0799 |  | 386 | -0.125(-0.245,-0.005) | 0.0414 |
| 3-6mo | q4 | 137 | -0.006(-0.117,0.106) | | | 0.9168 |  | 386 | -0.052(-0.153,0.049) | 0.3134 |
| 6-12mo | q1 | 112 | ref | | | ref |  | 464 | ref | ref |
| 6-12mo | q2 | 134 | -0.045(-0.093,0.003) | | | 0.0645 |  | 464 | -0.037(-0.086,0.011) | 0.1345 |
| 6-12mo | q3 | 131 | -0.046(-0.094,0.003) | | | 0.0675 |  | 464 | -0.049(-0.098,-0.000) | 0.0489 |
| 6-12mo | q4 | 132 | -0.048(-0.097,0.001) | | | 0.0565 |  | 464 | -0.042(-0.093,0.009) | 0.1038 |
| 12-18mo | q1 | 105 | ref | | | ref |  | 515 | ref | ref |
| 12-18mo | q2 | 127 | -0.014(-0.067,0.039) | | | 0.6107 |  | 515 | 0.022(-0.048,0.092) | 0.5369 |
| 12-18mo | q3 | 140 | 0.013(-0.045,0.071) | | | 0.6663 |  | 515 | 0.038(-0.026,0.103) | 0.2423 |
| 12-18mo | q4 | 166 | 0.016(-0.071,0.103) | | | 0.7151 |  | 515 | 0.044(-0.068,0.157) | 0.441 |
|  |  | **Lactulose Excretion Fraction** | | | | | | | | |
|  |  | Unadjusted | | | | |  | Adjusted | | |
| Interval | Quartile | N | β (95%CI) | | | p-value |  | N | β (95%CI) | p-value |
| 3-6mo | q1 | 93 | ref | | | ref |  | 389 | ref | ref |
| 3-6mo | q2 | 127 | -0.032(-0.139,0.075) | | | 0.554 |  | 389 | -0.006(-0.105,0.094) | 0.9118 |
| 3-6mo | q3 | 131 | -0.015(-0.133,0.102) | | | 0.7997 |  | 389 | 0.022(-0.087,0.132) | 0.6864 |
| 3-6mo | q4 | 56 | 0.003(-0.184,0.190) | | | 0.974 |  | 389 | -0.012(-0.186,0.161) | 0.888 |
| 6-12mo | q1 | 177 | ref | | | ref |  | 465 | ref | ref |
| 6-12mo | q2 | 151 | 0.027(-0.016,0.070) | | | 0.2117 |  | 465 | 0.030(-0.015,0.075) | 0.1912 |
| 6-12mo | q3 | 118 | 0.045(-0.003,0.093) | | | 0.064 |  | 465 | 0.057(0.008,0.106) | 0.0228 |
| 6-12mo | q4 | 64 | 0.017(-0.034,0.069) | | | 0.5075 |  | 465 | 0.025(-0.023,0.074) | 0.3071 |
| 12-18mo | q1 | 201 | ref | | | ref |  | 533 | ref | ref |
| 12-18mo | q2 | 153 | 0.051(0.009,0.093) | | | 0.0178 |  | 533 | 0.032(-0.014,0.079) | 0.1763 |
| 12-18mo | q3 | 131 | 0.055(-0.036,0.147) | | | 0.2378 |  | 533 | 0.021(-0.037,0.079) | 0.4814 |
| 12-18mo | q4 | 70 | 0.028(-0.005,0.062) | | | 0.0963 |  | 533 | 0.015(-0.031,0.062) | 0.5133 |
|  |  | **Mannitol excretion fraction** | | | | | | | | |
|  |  | Unadjusted | | | | |  | Adjusted | | |
| Interval | Quartile | N | β (95%CI) | | | p-value |  | N | β (95%CI) | p-value |
| 3-6mo | q1 | 129 | ref | | | ref |  | 389 | ref | ref |
| 3-6mo | q2 | 100 | -0.087(-0.195,0.021) | | | 0.1148 |  | 389 | -0.041(-0.145,0.064) | 0.4466 |
| 3-6mo | q3 | 104 | -0.102(-0.219,0.015) | | | 0.087 |  | 389 | -0.032(-0.133,0.069) | 0.5306 |
| 3-6mo | q4 | 71 | -0.046(-0.189,0.096) | | | 0.5245 |  | 389 | -0.035(-0.163,0.092) | 0.5873 |
| 6-12mo | q1 | 163 | ref | | | ref |  | 465 | ref | ref |
| 6-12mo | q2 | 158 | 0.026(-0.018,0.069) | | | 0.244 |  | 465 | 0.018(-0.027,0.064) | 0.4225 |
| 6-12mo | q3 | 125 | 0.035(-0.012,0.082) | | | 0.1401 |  | 465 | 0.029(-0.020,0.077) | 0.252 |
| 6-12mo | q4 | 63 | 0.031(-0.021,0.082) | | | 0.2478 |  | 465 | 0.051(0.005,0.097) | 0.0306 |
| 12-18mo | q1 | 177 | ref | | | ref |  | 533 | ref | ref |
| 12-18mo | q2 | 173 | 0.049(-0.023,0.121) | | | 0.1799 |  | 533 | 0.044(-0.028,0.116) | 0.2281 |
| 12-18mo | q3 | 138 | 0.010(-0.018,0.038) | | | 0.4828 |  | 533 | -0.023(-0.080,0.034) | 0.4282 |
| 12-18mo | q4 | 51 | 0.079(-0.016,0.173) | | | 0.1033 |  | 533 | 0.032(-0.070,0.133) | 0.538 |
|  |  | **soluble CD14** | | | | | | | | |
|  |  | Unadjusted | | | | |  | Adjusted | | |
| Interval | Quartile | N | β (95%CI) | p-value | | |  | N | β (95%CI) | p-value |
| 1-3mo | q1 | 277 | ref | ref | | |  | 520 | ref | ref |
| 1-3mo | q2 | 170 | 0.060(-0.084,0.204) | 0.4124 | | |  | 520 | 0.059(-0.084,0.201) | 0.4191 |
| 1-3mo | q3 | 76 | 0.064(-0.114,0.243) | 0.4804 | | |  | 520 | 0.070(-0.112,0.251) | 0.4504 |
| 1-3mo | q4 | 33 | -0.166(-0.497,0.166) | 0.3281 | | |  | 520 | -0.222(-0.592,0.148) | 0.2403 |
| 3-6mo | q1 | 216 | ref | ref | | |  | 693 | ref | ref |
| 3-6mo | q2 | 192 | -0.014(-0.169,0.141) | 0.8567 | | |  | 693 | -0.020(-0.182,0.142) | 0.813 |
| 3-6mo | q3 | 176 | -0.070(-0.233,0.093) | 0.3996 | | |  | 693 | -0.101(-0.285,0.083) | 0.2837 |
| 3-6mo | q4 | 110 | -0.113(-0.275,0.049) | 0.1724 | | |  | 693 | -0.104(-0.285,0.077) | 0.2596 |
| 6-12mo | q1 | 208 | ref | ref | | |  | 680 | ref | ref |
| 6-12mo | q2 | 183 | 0.001(-0.034,0.036) | 0.9501 | | |  | 680 | 0.010(-0.026,0.046) | 0.5988 |
| 6-12mo | q3 | 210 | -0.011(-0.049,0.027) | 0.5599 | | |  | 680 | -0.005(-0.044,0.033) | 0.7842 |
| 6-12mo | q4 | 196 | 0.004(-0.032,0.039) | 0.8444 | | |  | 680 | 0.005(-0.030,0.040) | 0.7756 |
| 12-18mo | q1 | 173 | ref | ref | | |  | 762 | ref | ref |
| 12-18mo | q2 | 186 | -0.017(-0.088,0.053) | 0.628 | | |  | 762 | -0.017(-0.083,0.048) | 0.6033 |
| 12-18mo | q3 | 223 | -0.006(-0.088,0.077) | 0.8907 | | |  | 762 | -0.028(-0.099,0.042) | 0.4288 |
| 12-18mo | q4 | 300 | -0.008(-0.079,0.064) | 0.8287 | | |  | 762 | -0.017(-0.096,0.063) | 0.6821 |
|  |  | **Myeloperoxidase** | | | | | | | | |
|  |  | Unadjusted | | | | |  | Adjusted | | |
| Interval | Quartile | N | β (95%CI) | p-value | | |  | N | β (95%CI) | p-value |
| 1-3mo | q1 | 74 | ref | ref | | |  | 398 | ref | ref |
| 1-3mo | q2 | 91 | 0.064(-0.181,0.309) | 0.6063 | | |  | 398 | -0.007(-0.246,0.232) | 0.9529 |
| 1-3mo | q3 | 107 | 0.012(-0.164,0.187) | 0.8954 | | |  | 398 | -0.002(-0.172,0.168) | 0.9792 |
| 1-3mo | q4 | 130 | 0.015(-0.144,0.174) | 0.8564 | | |  | 398 | -0.043(-0.198,0.111) | 0.5821 |
| 3-6mo | q1 | 53 | ref | ref | | |  | 475 | ref | ref |
| 3-6mo | q2 | 82 | -0.032(-0.230,0.167) | 0.7546 | | |  | 475 | 0.001(-0.184,0.186) | 0.9941 |
| 3-6mo | q3 | 117 | -0.110(-0.293,0.073) | 0.2376 | | |  | 475 | -0.066(-0.247,0.114) | 0.4708 |
| 3-6mo | q4 | 228 | -0.068(-0.252,0.117) | 0.4713 | | |  | 475 | -0.031(-0.211,0.148) | 0.731 |
| 6-12mo | q1 | 62 | ref | ref | | |  | 745 | ref | ref |
| 6-12mo | q2 | 158 | -0.042(-0.102,0.018) | 0.1712 | | |  | 745 | -0.038(-0.095,0.019) | 0.1877 |
| 6-12mo | q3 | 237 | -0.063(-0.122,-0.005) | 0.0342 | | |  | 745 | -0.056(-0.111,-0.001) | 0.0476 |
| 6-12mo | q4 | 288 | -0.050(-0.107,0.007) | 0.0865 | | |  | 745 | -0.050(-0.104,0.005) | 0.0739 |
| 12-18mo | q1 | 218 | ref | ref | | |  | 844 | ref | ref |
| 12-18mo | q2 | 284 | -0.002(-0.027,0.023) | 0.8752 | | |  | 844 | -0.007(-0.040,0.026) | 0.6718 |
| 12-18mo | q3 | 246 | 0.024(-0.030,0.079) | 0.3827 | | |  | 844 | 0.031(-0.029,0.090) | 0.3146 |
| 12-18mo | q4 | 134 | 0.020(-0.054,0.093) | 0.6034 | | |  | 844 | 0.023(-0.050,0.095) | 0.5399 |
|  |  | **Neopterin** | | | | | | | | |
|  |  | Unadjusted | | | | |  | Adjusted | | |
| Interval | Quartile | N | β (95%CI) | p-value | | |  | N | β (95%CI) | p-value |
| 1-3mo | q1 | 63 | ref | ref | | |  | 389 | ref | ref |
| 1-3mo | q2 | 145 | 0.080(-0.145,0.305) | 0.486 | | |  | 389 | 0.041(-0.168,0.250) | 0.6988 |
| 1-3mo | q3 | 120 | -0.071(-0.283,0.141) | 0.5103 | | |  | 389 | -0.087(-0.300,0.126) | 0.4221 |
| 1-3mo | q4 | 66 | 0.057(-0.159,0.272) | 0.6051 | | |  | 389 | -0.028(-0.255,0.198) | 0.8062 |
| 3-6mo | q1 | 30 | ref | ref | | |  | 461 | ref | ref |
| 3-6mo | q2 | 107 | 0.007(-0.132,0.146) | 0.9236 | | |  | 461 | -0.013(-0.135,0.110) | 0.8404 |
| 3-6mo | q3 | 140 | -0.043(-0.178,0.092) | 0.5302 | | |  | 461 | -0.054(-0.172,0.064) | 0.3714 |
| 3-6mo | q4 | 189 | 0.003(-0.123,0.128) | 0.9672 | | |  | 461 | -0.005(-0.114,0.104) | 0.9268 |
| 6-12mo | q1 | 40 | ref | ref | | |  | 682 | ref | ref |
| 6-12mo | q2 | 147 | 0.021(-0.037,0.078) | 0.4786 | | |  | 682 | -0.007(-0.061,0.046) | 0.7901 |
| 6-12mo | q3 | 265 | 0.018(-0.035,0.071) | 0.5127 | | |  | 682 | -0.023(-0.074,0.028) | 0.3705 |
| 6-12mo | q4 | 285 | 0.013(-0.041,0.067) | 0.639 | | |  | 682 | -0.024(-0.076,0.028) | 0.3708 |
| 12-18mo | q1 | 211 | ref | ref | | |  | 761 | ref | ref |
| 12-18mo | q2 | 224 | 0.002(-0.070,0.074) | 0.9613 | | |  | 761 | 0.007(-0.038,0.052) | 0.769 |
| 12-18mo | q3 | 217 | -0.010(-0.073,0.053) | 0.7622 | | |  | 761 | 0.023(-0.030,0.076) | 0.3895 |
| 12-18mo | q4 | 224 | -0.028(-0.086,0.030) | 0.3457 | | |  | 761 | -0.004(-0.053,0.044) | 0.8646 |
|  |  | **EE Score** | | | | | | | | |
|  |  | Unadjusted | | | | |  | Adjusted | | |
| Interval | Quartile | N | β (95%CI) | p-value | | |  | N | β (95%CI) | p-value |
| 1-3mo | q1 | 28 | ref | ref | | |  | 384 | ref | ref |
| 1-3mo | q2 | 106 | 0.164(-0.202,0.530) | 0.3801 | | |  | 384 | 0.017(-0.332,0.367) | 0.9225 |
| 1-3mo | q3 | 135 | 0.069(-0.305,0.443) | 0.718 | | |  | 384 | -0.000(-0.360,0.360) | 0.9996 |
| 1-3mo | q4 | 120 | 0.098(-0.256,0.453) | 0.5866 | | |  | 384 | -0.010(-0.357,0.337) | 0.9557 |
| 3-6mo | q1 | 17 | ref | ref | | |  | 455 | ref | ref |
| 3-6mo | q2 | 86 | 0.197(-0.120,0.513) | 0.2235 | | |  | 455 | 0.229(-0.039,0.496) | 0.0935 |
| 3-6mo | q3 | 145 | 0.142(-0.174,0.459) | 0.3781 | | |  | 455 | 0.175(-0.095,0.445) | 0.2036 |
| 3-6mo | q4 | 212 | 0.127(-0.187,0.441) | 0.4284 | | |  | 455 | 0.188(-0.084,0.460) | 0.1763 |
| 6-12mo | q1 | 38 | ref | ref | | |  | 681 | ref | ref |
| 6-12mo | q2 | 138 | -0.056(-0.142,0.029) | 0.1968 | | |  | 681 | -0.061(-0.139,0.018) | 0.1308 |
| 6-12mo | q3 | 273 | -0.077(-0.161,0.007) | 0.0741 | | |  | 681 | -0.077(-0.157,0.002) | 0.0576 |
| 6-12mo | q4 | 287 | -0.065(-0.148,0.019) | 0.1292 | | |  | 681 | -0.074(-0.151,0.003) | 0.0589 |
| 12-18mo | q1 | 118 | ref | ref | | |  | 760 | ref | ref |
| 12-18mo | q2 | 263 | -0.002(-0.037,0.033) | 0.906 | | |  | 760 | 0.009(-0.043,0.062) | 0.7238 |
| 12-18mo | q3 | 298 | 0.046(-0.012,0.104) | 0.117 | | |  | 760 | 0.032(-0.038,0.102) | 0.3672 |
| 12-18mo | q4 | 194 | 0.004(-0.028,0.036) | 0.8077 | | |  | 760 | 0.015(-0.038,0.069) | 0.5747 |
|  |  | **C-reactive Protein** | | | | | | | | |
|  |  | Unadjusted | | | | |  | Adjusted | | |
| Interval | Quartile | N | β (95%CI) | p-value | | |  | N | β (95%CI) | p-value |
| 1-3mo | q1 | 217 | ref | ref | | |  | 520 | ref | ref |
| 1-3mo | q2 | 132 | -0.016(-0.207,0.174) | 0.8667 | | |  | 520 | -0.037(-0.215,0.141) | 0.6851 |
| 1-3mo | q3 | 114 | 0.024(-0.117,0.165) | 0.7381 | | |  | 520 | 0.040(-0.111,0.192) | 0.6036 |
| 1-3mo | q4 | 93 | -0.066(-0.224,0.091) | 0.4102 | | |  | 520 | -0.049(-0.213,0.116) | 0.5609 |
| 3-6mo | q1 | 144 | ref | ref | | |  | 659 | ref | ref |
| 3-6mo | q2 | 210 | 0.074(-0.087,0.236) | 0.3681 | | |  | 659 | 0.054(-0.100,0.208) | 0.4899 |
| 3-6mo | q3 | 153 | 0.046(-0.045,0.137) | 0.3236 | | |  | 659 | 0.034(-0.055,0.123) | 0.4523 |
| 3-6mo | q4 | 187 | 0.039(-0.042,0.121) | 0.3466 | | |  | 659 | 0.033(-0.054,0.119) | 0.46 |
| 6-12mo | q1 | 161 | ref | ref | | |  | 680 | ref | ref |
| 6-12mo | q2 | 210 | -0.005(-0.040,0.031) | 0.804 | | |  | 680 | -0.009(-0.047,0.029) | 0.6519 |
| 6-12mo | q3 | 225 | 0.009(-0.028,0.046) | 0.636 | | |  | 680 | 0.008(-0.030,0.046) | 0.6965 |
| 6-12mo | q4 | 201 | 0.026(-0.009,0.062) | 0.1407 | | |  | 680 | 0.013(-0.024,0.051) | 0.4888 |
| 12-18mo | q1 | 163 | ref | ref | | |  | 762 | ref | ref |
| 12-18mo | q2 | 239 | 0.072(0.007,0.137) | 0.0311 | | |  | 762 | 0.059(-0.001,0.120) | 0.0553 |
| 12-18mo | q3 | 243 | 0.019(-0.006,0.044) | 0.134 | | |  | 762 | 0.011(-0.029,0.051) | 0.6003 |
| 12-18mo | q4 | 237 | 0.019(-0.012,0.050) | 0.229 | | |  | 762 | 0.015(-0.025,0.054) | 0.47 |
|  |  | **Kynurenine:tryptophan ratio** | | | | | | | | |
|  |  | Unadjusted | | | | |  | Adjusted | | |
| Interval | Quartile | N | β (95%CI) | p-value | | |  | N | β (95%CI) | p-value |
| 1-3mo | q1 | 44 | ref | ref | | |  | 445 | ref | ref |
| 1-3mo | q2 | 93 | -0.091(-0.294,0.113) | 0.384 | | |  | 445 | -0.009(-0.200,0.182) | 0.927 |
| 1-3mo | q3 | 150 | -0.044(-0.211,0.122) | 0.6011 | | |  | 445 | -0.010(-0.186,0.166) | 0.9098 |
| 1-3mo | q4 | 186 | -0.022(-0.200,0.157) | 0.8101 | | |  | 445 | 0.044(-0.146,0.234) | 0.6506 |
| 3-6mo | q1 | 143 | ref | ref | | |  | 611 | ref | ref |
| 3-6mo | q2 | 179 | -0.156(-0.277,-0.035) | 0.0115 | | |  | 611 | -0.119(-0.253,0.015) | 0.0822 |
| 3-6mo | q3 | 156 | -0.007(-0.106,0.092) | 0.8919 | | |  | 611 | 0.036(-0.086,0.157) | 0.5664 |
| 3-6mo | q4 | 134 | 0.042(-0.181,0.264) | 0.714 | | |  | 611 | 0.099(-0.200,0.399) | 0.5163 |
| 6-12mo | q1 | 235 | ref | ref | | |  | 665 | ref | ref |
| 6-12mo | q2 | 192 | 0.021(-0.012,0.054) | 0.2202 | | |  | 665 | 0.030(-0.003,0.064) | 0.0762 |
| 6-12mo | q3 | 159 | -0.007(-0.048,0.033) | 0.7209 | | |  | 665 | -0.004(-0.043,0.034) | 0.8217 |
| 6-12mo | q4 | 139 | 0.010(-0.028,0.047) | 0.6192 | | |  | 665 | 0.002(-0.035,0.039) | 0.915 |
| 12-18mo | q1 | 238 | ref | ref | | |  | 772 | ref | ref |
| 12-18mo | q2 | 201 | 0.019(-0.033,0.070) | 0.4779 | | |  | 772 | 0.008(-0.041,0.056) | 0.7561 |
| 12-18mo | q3 | 182 | 0.049(-0.024,0.121) | 0.1889 | | |  | 772 | 0.030(-0.026,0.085) | 0.2917 |
| 12-18mo | q4 | 180 | -0.008(-0.033,0.016) | 0.5139 | | |  | 772 | -0.034(-0.074,0.007) | 0.1003 |
|  |  | **Kynurenine** | | | | | | | | |
|  |  | Unadjusted | | | | |  | Adjusted | | |
| Interval | Quartile | N | β (95%CI) | p-value | | |  | N | β (95%CI) | p-value |
| 1-3mo | q1 | 40 | ref | ref | | |  | 496 | ref | ref |
| 1-3mo | q2 | 56 | -0.269(-0.542,0.004) | 0.0531 | | |  | 496 | -0.175(-0.460,0.110) | 0.2295 |
| 1-3mo | q3 | 123 | 0.050(-0.183,0.284) | 0.673 | | |  | 496 | 0.096(-0.135,0.327) | 0.4134 |
| 1-3mo | q4 | 310 | -0.085(-0.256,0.086) | 0.3295 | | |  | 496 | -0.036(-0.213,0.141) | 0.6915 |
| 3-6mo | q1 | 91 | ref | ref | | |  | 657 | ref | ref |
| 3-6mo | q2 | 171 | -0.031(-0.172,0.109) | 0.6601 | | |  | 657 | -0.025(-0.165,0.114) | 0.7233 |
| 3-6mo | q3 | 199 | -0.003(-0.158,0.151) | 0.9647 | | |  | 657 | 0.006(-0.151,0.164) | 0.9358 |
| 3-6mo | q4 | 197 | 0.069(-0.126,0.263) | 0.4896 | | |  | 657 | 0.109(-0.132,0.349) | 0.3766 |
| 6-12mo | q1 | 230 | ref | ref | | |  | 683 | ref | ref |
| 6-12mo | q2 | 249 | -0.016(-0.050,0.017) | 0.3338 | | |  | 683 | -0.025(-0.057,0.006) | 0.1158 |
| 6-12mo | q3 | 185 | -0.021(-0.056,0.015) | 0.2498 | | |  | 683 | -0.030(-0.066,0.006) | 0.1023 |
| 6-12mo | q4 | 81 | -0.005(-0.052,0.042) | 0.8373 | | |  | 683 | -0.022(-0.069,0.024) | 0.3472 |
| 12-18mo | q1 | 349 | ref | ref | | |  | 794 | ref | ref |
| 12-18mo | q2 | 224 | 0.012(-0.046,0.069) | 0.695 | | |  | 794 | 0.018(-0.038,0.073) | 0.5283 |
| 12-18mo | q3 | 180 | -0.015(-0.055,0.024) | 0.4501 | | |  | 794 | 0.000(-0.036,0.036) | 0.9828 |
| 12-18mo | q4 | 71 | 0.005(-0.071,0.082) | 0.8931 | | |  | 794 | 0.012(-0.052,0.076) | 0.7126 |
|  |  | **Tryptophan** | | | | | | | | |
|  |  | Unadjusted | | | | |  | Adjusted | | |
| Interval | Quartile | N | β (95%CI) | p-value | | |  | N | β (95%CI) | p-value |
| 1-3mo | q1 | 56 | ref | ref | | |  | 492 | ref | ref |
| 1-3mo | q2 | 106 | 0.131(-0.156,0.417) | 0.3713 | | |  | 492 | 0.177(-0.125,0.479) | 0.2503 |
| 1-3mo | q3 | 151 | -0.032(-0.284,0.220) | 0.802 | | |  | 492 | 0.034(-0.232,0.300) | 0.8021 |
| 1-3mo | q4 | 216 | 0.031(-0.211,0.274) | 0.7992 | | |  | 492 | 0.064(-0.191,0.320) | 0.6209 |
| 3-6mo | q1 | 85 | ref | ref | | |  | 657 | ref | ref |
| 3-6mo | q2 | 170 | 0.128(-0.073,0.329) | 0.212 | | |  | 657 | 0.134(-0.078,0.345) | 0.2157 |
| 3-6mo | q3 | 194 | 0.024(-0.102,0.149) | 0.7113 | | |  | 657 | 0.010(-0.115,0.135) | 0.8763 |
| 3-6mo | q4 | 209 | 0.043(-0.091,0.177) | 0.5282 | | |  | 657 | 0.032(-0.101,0.165) | 0.6361 |
| 6-12mo | q1 | 212 | ref | ref | | |  | 639 | ref | ref |
| 6-12mo | q2 | 212 | -0.010(-0.049,0.030) | 0.6339 | | |  | 639 | -0.012(-0.051,0.028) | 0.5725 |
| 6-12mo | q3 | 176 | -0.002(-0.037,0.033) | 0.9036 | | |  | 639 | -0.013(-0.047,0.021) | 0.4634 |
| 6-12mo | q4 | 145 | -0.021(-0.055,0.013) | 0.2317 | | |  | 639 | -0.026(-0.060,0.009) | 0.1464 |
| 12-18mo | q1 | 351 | ref | ref | | |  | 794 | ref | ref |
| 12-18mo | q2 | 201 | -0.030(-0.072,0.011) | 0.1512 | | |  | 794 | -0.027(-0.070,0.015) | 0.2099 |
| 12-18mo | q3 | 167 | -0.000(-0.067,0.067) | 0.9988 | | |  | 794 | 0.005(-0.061,0.071) | 0.8749 |
| 12-18mo | q4 | 105 | -0.000(-0.060,0.059) | 0.9918 | | |  | 794 | 0.010(-0.044,0.065) | 0.7174 |
|  |  | **Insulin-like growth factor 1** | | | | | | | | |
|  |  | Unadjusted | | | | |  | Adjusted | | |
| Interval | Quartile | N | β (95%CI) | | p-value | |  | N | β (95%CI) | p-value |
| 1-3mo | q1 | 94 | ref | | ref | |  | 518 | ref | ref |
| 1-3mo | q2 | 70 | 0.026(-0.218,0.269) | | 0.8359 | |  | 518 | 0.048(-0.187,0.282) | 0.6898 |
| 1-3mo | q3 | 114 | 0.118(-0.078,0.314) | | 0.2393 | |  | 518 | 0.168(-0.026,0.362) | 0.0889 |
| 1-3mo | q4 | 276 | 0.128(-0.055,0.311) | | 0.1695 | |  | 518 | 0.191(0.011,0.370) | 0.0372 |
| 3-6mo | q1 | 131 | ref | | ref | |  | 692 | ref | ref |
| 3-6mo | q2 | 160 | -0.055(-0.273,0.162) | | 0.6175 | |  | 692 | -0.050(-0.252,0.152) | 0.6255 |
| 3-6mo | q3 | 197 | -0.070(-0.171,0.030) | | 0.1704 | |  | 692 | -0.058(-0.151,0.035) | 0.2211 |
| 3-6mo | q4 | 205 | -0.082(-0.184,0.021) | | 0.1177 | |  | 692 | -0.102(-0.196,-0.008) | 0.0331 |
| 6-12mo | q1 | 200 | ref | | ref | |  | 680 | ref | ref |
| 6-12mo | q2 | 259 | 0.026(-0.010,0.062) | | 0.1605 | |  | 680 | 0.025(-0.010,0.059) | 0.1671 |
| 6-12mo | q3 | 202 | -0.003(-0.039,0.033) | | 0.8654 | |  | 680 | -0.017(-0.055,0.020) | 0.3669 |
| 6-12mo | q4 | 136 | 0.045(0.013,0.078) | | 0.006 | |  | 680 | 0.029(-0.005,0.062) | 0.0916 |
| 12-18mo | q1 | 269 | ref | | ref | |  | 762 | ref | ref |
| 12-18mo | q2 | 255 | 0.048(-0.006,0.102) | | 0.0801 | |  | 762 | 0.047(-0.015,0.110) | 0.1353 |
| 12-18mo | q3 | 233 | 0.025(-0.020,0.070) | | 0.2687 | |  | 762 | 0.019(-0.015,0.053) | 0.2705 |
| 12-18mo | q4 | 125 | 0.012(-0.014,0.038) | | 0.3703 | |  | 762 | 0.011(-0.027,0.049) | 0.5819 |

**Supplementary Table 10.** Mean change in LAZ (SD/month) for children with biomarker concentrations at the 1st quartile, 4th quartile and interquartile range of biomarker concentration at the start of each follow up interval.

|  |  | **Intestinal fatty acid binding protein** | | | | | | |
| --- | --- | --- | --- | --- | --- | --- | --- | --- |
| Interval | Quantile | Unadjusted | | |  | Adjusted | | |
|  |  | N | β (95%CI) | p-value |  | N | β (95%CI) | p-value |
| 1-3mo | q1 | 66 | ref | ref |  | 64 | ref | ref |
| 1-3mo | IQR | 288 | -0.075(-0.271,0.121) | 0.455 |  | 262 | -0.039(-0.231,0.153) | 0.6928 |
| 1-3mo | q4 | 203 | -0.018(-0.203,0.168) | 0.8529 |  | 195 | 0.048(-0.135,0.231) | 0.6055 |
| 3-6mo | q1 | 256 | ref | ref |  | 255 | ref | ref |
| 3-6mo | IQR | 372 | 0.049(-0.052,0.150) | 0.3392 |  | 372 | 0.051(-0.065,0.167) | 0.3854 |
| 3-6mo | q4 | 65 | -0.119(-0.319,0.082) | 0.2456 |  | 65 | -0.081(-0.287,0.126) | 0.4437 |
| 6-12mo | q1 | 315 | ref | ref |  | 259 | ref | ref |
| 6-12mo | IQR | 370 | -0.007(-0.035,0.021) | 0.6324 |  | 327 | -0.005(-0.034,0.024) | 0.7254 |
| 6-12mo | q4 | 112 | 0.012(-0.026,0.050) | 0.5381 |  | 94 | 0.024(-0.014,0.061) | 0.216 |
| 12-18mo | q1 | 176 | ref | ref |  | 146 | ref | ref |
| 12-18mo | IQR | 448 | 0.009(-0.054,0.071) | 0.7852 |  | 388 | 0.035(-0.000,0.069) | 0.0502 |
| 12-18mo | q4 | 258 | -0.002(-0.059,0.055) | 0.9532 |  | 228 | 0.035(0.003,0.066) | 0.0308 |
|  |  | **Regenerating protein 1-β** | | | | | | |
| Interval | Quantile | Unadjusted | | |  | Adjusted | | |
|  |  | N | β (95%CI) | p-value |  | N | β (95%CI) | p-value |
| 1-3mo | q1 | 186 | ref | ref |  | 185 | ref | ref |
| 1-3mo | IQR | 167 | -0.096(-0.252,0.060) | 0.2259 |  | 164 | -0.112(-0.262,0.039) | 0.147 |
| 1-3mo | q4 | 16 | -0.125(-0.377,0.128) | 0.3321 |  | 16 | -0.104(-0.363,0.156) | 0.4335 |
| 3-6mo | q1 | 126 | ref | ref |  | 125 | ref | ref |
| 3-6mo | IQR | 259 | -0.005(-0.100,0.090) | 0.9167 |  | 255 | 0.001(-0.087,0.089) | 0.9906 |
| 3-6mo | q4 | 53 | -0.029(-0.147,0.090) | 0.6376 |  | 53 | -0.022(-0.137,0.092) | 0.7009 |
| 6-12mo | q1 | 73 | ref | ref |  | 73 | ref | ref |
| 6-12mo | IQR | 426 | 0.005(-0.039,0.048) | 0.8319 |  | 426 | 0.008(-0.033,0.048) | 0.7173 |
| 6-12mo | q4 | 223 | -0.010(-0.054,0.034) | 0.6575 |  | 223 | -0.003(-0.044,0.039) | 0.8962 |
| 12-18mo | q1 | 133 | ref | ref |  | 118 | ref | ref |
| 12-18mo | IQR | 459 | 0.036(-0.007,0.078) | 0.0984 |  | 403 | 0.033(-0.026,0.092) | 0.2759 |
| 12-18mo | q4 | 277 | -0.002(-0.031,0.026) | 0.8689 |  | 234 | 0.014(-0.034,0.062) | 0.5669 |
|  |  | **Citrulline** | | | | | | |
| Interval | Quantile | Unadjusted | | |  | Adjusted | | |
|  |  | N | β (95%CI) | p-value |  | N | β (95%CI) | p-value |
| 1-3mo | q1 | 144 | ref | ref |  | 135 | ref | ref |
| 1-3mo | IQR | 280 | 0.130(-0.048,0.309) | 0.1523 |  | 261 | 0.087(-0.094,0.267) | 0.3465 |
| 1-3mo | q4 | 105 | 0.168(-0.042,0.378) | 0.1167 |  | 100 | 0.180(-0.030,0.390) | 0.0935 |
| 3-6mo | q1 | 200 | ref | ref |  | 199 | ref | ref |
| 3-6mo | IQR | 358 | 0.037(-0.077,0.151) | 0.5266 |  | 358 | 0.023(-0.089,0.135) | 0.6881 |
| 3-6mo | q4 | 100 | 0.068(-0.047,0.183) | 0.2475 |  | 100 | 0.040(-0.091,0.170) | 0.5535 |
| 6-12mo | q1 | 209 | ref | ref |  | 193 | ref | ref |
| 6-12mo | IQR | 372 | 0.015(-0.015,0.045) | 0.3234 |  | 338 | -0.002(-0.029,0.025) | 0.8929 |
| 6-12mo | q4 | 164 | 0.011(-0.028,0.050) | 0.5896 |  | 152 | -0.006(-0.042,0.030) | 0.7362 |
| 12-18mo | q1 | 133 | ref | ref |  | 119 | ref | ref |
| 12-18mo | IQR | 375 | -0.036(-0.128,0.057) | 0.447 |  | 336 | -0.053(-0.138,0.032) | 0.2227 |
| 12-18mo | q4 | 316 | -0.047(-0.137,0.043) | 0.3099 |  | 260 | -0.071(-0.181,0.040) | 0.2089 |
|  |  | **Alpha-1 Antitrypsin** | | | | | | |
| Interval | Quantile | Unadjusted | | |  | Adjusted | | |
|  |  | N | β (95%CI) | p-value |  | N | β (95%CI) | p-value |
| 1-3mo | q1 | 78 | ref | ref |  | 77 | ref | ref |
| 1-3mo | IQR | 167 | -0.066(-0.281,0.149) | 0.548 |  | 167 | -0.057(-0.264,0.150) | 0.5916 |
| 1-3mo | q4 | 152 | -0.065(-0.250,0.119) | 0.4888 |  | 148 | -0.074(-0.261,0.113) | 0.4353 |
| 3-6mo | q1 | 87 | ref | ref |  | 86 | ref | ref |
| 3-6mo | IQR | 229 | 0.071(-0.040,0.182) | 0.2102 |  | 226 | 0.070(-0.026,0.166) | 0.1525 |
| 3-6mo | q4 | 156 | 0.056(-0.071,0.184) | 0.3843 |  | 155 | 0.079(-0.035,0.193) | 0.1755 |
| 6-12mo | q1 | 142 | ref | ref |  | 127 | ref | ref |
| 6-12mo | IQR | 399 | -0.009(-0.042,0.024) | 0.5873 |  | 351 | 0.001(-0.034,0.036) | 0.9562 |
| 6-12mo | q4 | 203 | -0.033(-0.070,0.004) | 0.0845 |  | 183 | -0.020(-0.059,0.020) | 0.3285 |
| 12-18mo | q1 | 218 | ref | ref |  | 190 | ref | ref |
| 12-18mo | IQR | 457 | 0.012(-0.042,0.067) | 0.6542 |  | 401 | 0.034(-0.005,0.074) | 0.0894 |
| 12-18mo | q4 | 208 | 0.002(-0.047,0.050) | 0.9395 |  | 176 | 0.022(-0.011,0.054) | 0.1943 |
|  |  | **Lactulose:mannitol ratio** | | | | | | |
| Interval | Quantile | Unadjusted | | |  | Adjusted | | |
|  |  | N | β (95%CI) | p-value |  | N | β (95%CI) | p-value |
| 3-6mo | q1 | 78 | ref | ref |  | 75 | ref | ref |
| 3-6mo | IQR | 189 | -0.065(-0.167,0.036) | 0.2087 |  | 178 | -0.080(-0.176,0.017) | 0.105 |
| 3-6mo | q4 | 137 | -0.006(-0.117,0.106) | 0.9168 |  | 133 | -0.051(-0.152,0.050) | 0.3213 |
| 6-12mo | q1 | 112 | ref | ref |  | 101 | ref | ref |
| 6-12mo | IQR | 265 | -0.045(-0.087,-0.003) | 0.035 |  | 244 | -0.049(-0.094,-0.004) | 0.0315 |
| 6-12mo | q4 | 132 | -0.048(-0.097,0.001) | 0.0565 |  | 119 | -0.050(-0.103,0.003) | 0.0639 |
| 12-18mo | q1 | 105 | ref | ref |  | 102 | ref | ref |
| 12-18mo | IQR | 267 | 0.000(-0.053,0.053) | 0.9963 |  | 255 | 0.031(-0.033,0.094) | 0.3452 |
| 12-18mo | q4 | 166 | 0.016(-0.071,0.103) | 0.7151 |  | 158 | 0.044(-0.068,0.157) | 0.4412 |
|  |  | **Lactulose Excretion Fracion** | | | | | | |
| Interval | Quantile | Unadjusted | | |  | Adjusted | | |
|  |  | N | β (95%CI) | p-value |  | N | β (95%CI) | p-value |
| 3-6mo | q1 | 93 | ref | ref |  | 90 | ref | ref |
| 3-6mo | IQR | 258 | -0.018(-0.119,0.083) | 0.7255 |  | 247 | 0.016(-0.078,0.109) | 0.7427 |
| 3-6mo | q4 | 56 | 0.007(-0.180,0.194) | 0.9398 |  | 52 | -0.007(-0.180,0.165) | 0.9325 |
| 6-12mo | q1 | 177 | ref | ref |  | 163 | ref | ref |
| 6-12mo | IQR | 269 | 0.037(-0.001,0.074) | 0.0562 |  | 245 | 0.043(0.004,0.083) | 0.0318 |
| 6-12mo | q4 | 64 | 0.018(-0.033,0.070) | 0.4878 |  | 57 | 0.025(-0.023,0.074) | 0.2995 |
| 12-18mo | q1 | 201 | ref | ref |  | 188 | ref | ref |
| 12-18mo | IQR | 284 | 0.052(0.002,0.101) | 0.0413 |  | 274 | 0.025(-0.008,0.057) | 0.1376 |
| 12-18mo | q4 | 70 | 0.027(-0.007,0.061) | 0.1146 |  | 69 | 0.013(-0.033,0.060) | 0.5709 |
|  |  | **Mannitol excretion fraction** | | | | | | |
| Interval | Quantile | Unadjusted | | |  | Adjusted | | |
|  |  | N | β (95%CI) | p-value |  | N | β (95%CI) | p-value |
| 3-6mo | q1 | 129 | ref | ref |  | 123 | ref | ref |
| 3-6mo | IQR | 204 | -0.077(-0.174,0.020) | 0.121 |  | 196 | -0.019(-0.105,0.067) | 0.6692 |
| 3-6mo | q4 | 71 | -0.035(-0.179,0.110) | 0.6395 |  | 67 | -0.029(-0.160,0.101) | 0.6612 |
| 6-12mo | q1 | 163 | ref | ref |  | 147 | ref | ref |
| 6-12mo | IQR | 283 | 0.031(-0.006,0.068) | 0.0998 |  | 263 | 0.028(-0.013,0.068) | 0.177 |
| 6-12mo | q4 | 63 | 0.027(-0.024,0.078) | 0.3043 |  | 54 | 0.038(-0.012,0.087) | 0.1356 |
| 12-18mo | q1 | 177 | ref | ref |  | 166 | ref | ref |
| 12-18mo | IQR | 311 | 0.026(-0.018,0.070) | 0.246 |  | 300 | 0.007(-0.029,0.043) | 0.6933 |
| 12-18mo | q4 | 51 | 0.074(-0.024,0.172) | 0.1398 |  | 50 | 0.024(-0.081,0.129) | 0.6526 |
|  |  | **soluble CD14** | | | | | | |
| Interval | Quantile | Unadjusted | | |  | Adjusted | | |
|  |  | N | β (95%CI) | p-value |  | N | β (95%CI) | p-value |
| 1-3mo | q1 | 277 | ref | ref |  | 259 | ref | ref |
| 1-3mo | IQR | 246 | 0.061(-0.062,0.185) | 0.3301 |  | 231 | 0.062(-0.062,0.186) | 0.3254 |
| 1-3mo | q4 | 33 | -0.166(-0.497,0.166) | 0.3281 |  | 30 | -0.222(-0.592,0.148) | 0.2394 |
| 3-6mo | q1 | 216 | ref | ref |  | 215 | ref | ref |
| 3-6mo | IQR | 368 | -0.041(-0.193,0.111) | 0.5983 |  | 368 | -0.058(-0.226,0.109) | 0.4967 |
| 3-6mo | q4 | 110 | -0.113(-0.275,0.049) | 0.1724 |  | 110 | -0.104(-0.285,0.077) | 0.2598 |
| 6-12mo | q1 | 208 | ref | ref |  | 179 | ref | ref |
| 6-12mo | IQR | 393 | -0.006(-0.037,0.026) | 0.7311 |  | 333 | 0.002(-0.031,0.034) | 0.9199 |
| 6-12mo | q4 | 196 | 0.004(-0.032,0.039) | 0.8444 |  | 168 | 0.005(-0.030,0.041) | 0.7741 |
| 12-18mo | q1 | 173 | ref | ref |  | 150 | ref | ref |
| 12-18mo | IQR | 409 | -0.011(-0.084,0.062) | 0.7669 |  | 352 | -0.024(-0.090,0.043) | 0.4851 |
| 12-18mo | q4 | 300 | -0.008(-0.079,0.064) | 0.8287 |  | 260 | -0.017(-0.096,0.063) | 0.6826 |
|  |  | **Myeloperoxidase** | | | | | | |
|  |  | Unadjusted | | |  | Adjusted | | |
| Interval | Quantile | N | β (95%CI) | p-value |  | N | β (95%CI) | p-value |
| 1-3mo | q1 | 74 | ref | ref |  | 73 | ref | ref |
| 1-3mo | IQR | 198 | 0.036(-0.133,0.205) | 0.6757 |  | 196 | -0.005(-0.167,0.158) | 0.9564 |
| 1-3mo | q4 | 130 | 0.015(-0.144,0.174) | 0.8564 |  | 129 | -0.043(-0.198,0.111) | 0.5825 |
| 3-6mo | q1 | 53 | ref | ref |  | 53 | ref | ref |
| 3-6mo | IQR | 199 | -0.078(-0.260,0.105) | 0.4028 |  | 195 | -0.038(-0.214,0.138) | 0.6705 |
| 3-6mo | q4 | 228 | -0.068(-0.252,0.117) | 0.4713 |  | 227 | -0.031(-0.210,0.148) | 0.7359 |
| 6-12mo | q1 | 62 | ref | ref |  | 62 | ref | ref |
| 6-12mo | IQR | 395 | -0.055(-0.111,0.002) | 0.0583 |  | 395 | -0.049(-0.102,0.005) | 0.0757 |
| 6-12mo | q4 | 288 | -0.050(-0.107,0.007) | 0.0865 |  | 288 | -0.049(-0.104,0.005) | 0.0754 |
| 12-18mo | q1 | 218 | ref | ref |  | 188 | ref | ref |
| 12-18mo | IQR | 530 | 0.010(-0.021,0.042) | 0.5215 |  | 462 | 0.008(-0.030,0.046) | 0.684 |
| 12-18mo | q4 | 134 | 0.020(-0.054,0.093) | 0.6034 |  | 116 | -0.021(-0.058,0.015) | 0.2566 |
|  |  | **Neopterin** | | | | | | |
| Interval | Quantile | Unadjusted | | |  | Adjusted | | |
|  |  | N | β (95%CI) | p-value |  | N | β (95%CI) | p-value |
| 1-3mo | q1 | 63 | ref | ref |  | 63 | ref | ref |
| 1-3mo | IQR | 265 | 0.011(-0.187,0.210) | 0.9097 |  | 261 | -0.017(-0.208,0.173) | 0.859 |
| 1-3mo | q4 | 66 | 0.057(-0.159,0.272) | 0.6051 |  | 65 | -0.025(-0.250,0.201) | 0.8311 |
| 3-6mo | q1 | 30 | ref | ref |  | 30 | ref | ref |
| 3-6mo | IQR | 247 | -0.022(-0.146,0.103) | 0.7348 |  | 244 | -0.036(-0.144,0.071) | 0.5087 |
| 3-6mo | q4 | 189 | 0.003(-0.123,0.128) | 0.9672 |  | 187 | -0.005(-0.114,0.104) | 0.9255 |
| 6-12mo | q1 | 40 | ref | ref |  | 34 | ref | ref |
| 6-12mo | IQR | 412 | 0.019(-0.033,0.071) | 0.4782 |  | 369 | 0.020(-0.030,0.071) | 0.4295 |
| 6-12mo | q4 | 285 | 0.013(-0.041,0.067) | 0.639 |  | 253 | 0.013(-0.040,0.066) | 0.6231 |
| 12-18mo | q1 | 211 | ref | ref |  | 186 | ref | ref |
| 12-18mo | IQR | 441 | -0.004(-0.066,0.059) | 0.9025 |  | 382 | 0.015(-0.031,0.060) | 0.5271 |
| 12-18mo | q4 | 224 | -0.028(-0.086,0.030) | 0.3457 |  | 193 | -0.004(-0.053,0.044) | 0.8657 |
|  |  | **EE Score** | | | | | | |
| Interval | Quantile | Unadjusted | | |  | Adjusted | | |
|  |  | N | β (95%CI) | p-value |  | N | β (95%CI) | p-value |
| 1-3mo | q1 | 28 | ref | ref |  | 28 | ref | ref |
| 1-3mo | IQR | 241 | 0.111(-0.247,0.468) | 0.5435 |  | 239 | 0.007(-0.333,0.347) | 0.9674 |
| 1-3mo | q4 | 120 | 0.098(-0.256,0.453) | 0.5866 |  | 117 | -0.010(-0.357,0.337) | 0.9539 |
| 3-6mo | q1 | 17 | ref | ref |  | 17 | ref | ref |
| 3-6mo | IQR | 231 | 0.163(-0.150,0.475) | 0.3085 |  | 227 | 0.195(-0.070,0.460) | 0.1486 |
| 3-6mo | q4 | 212 | 0.127(-0.187,0.441) | 0.4284 |  | 211 | 0.188(-0.084,0.459) | 0.1748 |
| 6-12mo | q1 | 38 | ref | ref |  | 35 | ref | ref |
| 6-12mo | IQR | 411 | -0.070(-0.153,0.013) | 0.0995 |  | 383 | -0.072(-0.149,0.006) | 0.072 |
| 6-12mo | q4 | 287 | -0.065(-0.148,0.019) | 0.1292 |  | 263 | -0.074(-0.151,0.003) | 0.0593 |
| 12-18mo | q1 | 118 | ref | ref |  | 105 | ref | ref |
| 12-18mo | IQR | 561 | 0.023(-0.016,0.063) | 0.2447 |  | 486 | 0.022(-0.036,0.080) | 0.4667 |
| 12-18mo | q4 | 194 | 0.004(-0.028,0.036) | 0.8077 |  | 169 | 0.015(-0.038,0.069) | 0.5716 |
|  |  | **C-reactive Protein** | | | | | | |
| Interval | Quantile | Unadjusted | | |  | Adjusted | | |
|  |  | N | β (95%CI) | p-value |  | N | β (95%CI) | p-value |
| 1-3mo | q1 | 217 | ref | ref |  | 198 | ref | ref |
| 1-3mo | IQR | 246 | 0.002(-0.126,0.131) | 0.9714 |  | 232 | -0.004(-0.128,0.121) | 0.9538 |
| 1-3mo | q4 | 93 | -0.066(-0.224,0.091) | 0.4102 |  | 90 | -0.052(-0.216,0.111) | 0.5299 |
| 3-6mo | q1 | 144 | ref | ref |  | 138 | ref | ref |
| 3-6mo | IQR | 363 | 0.062(-0.046,0.170) | 0.2592 |  | 346 | 0.045(-0.051,0.142) | 0.3578 |
| 3-6mo | q4 | 187 | 0.039(-0.042,0.121) | 0.3466 |  | 175 | 0.033(-0.054,0.119) | 0.4603 |
| 6-12mo | q1 | 161 | ref | ref |  | 142 | ref | ref |
| 6-12mo | IQR | 435 | 0.002(-0.029,0.034) | 0.8772 |  | 366 | -0.000(-0.034,0.033) | 0.9849 |
| 6-12mo | q4 | 201 | 0.026(-0.009,0.062) | 0.1407 |  | 172 | 0.013(-0.024,0.051) | 0.4869 |
| 12-18mo | q1 | 163 | ref | ref |  | 137 | ref | ref |
| 12-18mo | IQR | 482 | 0.045(0.008,0.082) | 0.0159 |  | 424 | 0.035(0.000,0.069) | 0.05 |
| 12-18mo | q4 | 237 | 0.019(-0.012,0.050) | 0.229 |  | 201 | 0.014(-0.025,0.054) | 0.4735 |
|  |  | **Kynurenine:tryptophan ratio** | | | | | | |
| Interval | Quantile | Unadjusted | | |  | Adjusted | | |
|  |  | N | β (95%CI) | p-value |  | N | β (95%CI) | p-value |
| 1-3mo | q1 | 44 | ref | ref |  | 41 | ref | ref |
| 1-3mo | IQR | 243 | -0.062(-0.219,0.095) | 0.4378 |  | 228 | -0.010(-0.168,0.148) | 0.9046 |
| 1-3mo | q4 | 186 | -0.022(-0.200,0.157) | 0.8101 |  | 176 | 0.044(-0.146,0.234) | 0.6503 |
| 3-6mo | q1 | 143 | ref | ref |  | 143 | ref | ref |
| 3-6mo | IQR | 335 | -0.087(-0.183,0.010) | 0.0787 |  | 334 | -0.048(-0.164,0.068) | 0.4177 |
| 3-6mo | q4 | 134 | 0.042(-0.181,0.264) | 0.714 |  | 134 | 0.097(-0.202,0.397) | 0.5253 |
| 6-12mo | q1 | 235 | ref | ref |  | 218 | ref | ref |
| 6-12mo | IQR | 351 | 0.008(-0.021,0.037) | 0.5926 |  | 317 | 0.015(-0.014,0.044) | 0.3152 |
| 6-12mo | q4 | 139 | 0.010(-0.028,0.047) | 0.6192 |  | 130 | 0.002(-0.035,0.039) | 0.918 |
| 12-18mo | q1 | 238 | ref | ref |  | 229 | ref | ref |
| 12-18mo | IQR | 383 | 0.033(-0.012,0.078) | 0.1543 |  | 366 | 0.018(-0.015,0.052) | 0.2842 |
| 12-18mo | q4 | 180 | -0.008(-0.033,0.016) | 0.5139 |  | 177 | -0.034(-0.074,0.007) | 0.1001 |
|  |  | **Kynurenine** | | | | | | |
| Interval | Quantile | Unadjusted | | |  | Adjusted | | |
|  |  | N | β (95%CI) | p-value |  | N | β (95%CI) | p-value |
| 1-3mo | q1 | 40 | ref | ref |  | 36 | ref | ref |
| 1-3mo | IQR | 179 | -0.050(-0.259,0.160) | 0.6424 |  | 171 | 0.012(-0.202,0.225) | 0.9134 |
| 1-3mo | q4 | 310 | -0.085(-0.256,0.086) | 0.3295 |  | 289 | -0.036(-0.214,0.142) | 0.689 |
| 3-6mo | q1 | 91 | ref | ref |  | 91 | ref | ref |
| 3-6mo | IQR | 370 | -0.016(-0.156,0.123) | 0.8178 |  | 369 | -0.009(-0.147,0.130) | 0.9037 |
| 3-6mo | q4 | 197 | 0.069(-0.126,0.263) | 0.4896 |  | 197 | 0.108(-0.132,0.348) | 0.3778 |
| 6-12mo | q1 | 230 | ref | ref |  | 208 | ref | ref |
| 6-12mo | IQR | 434 | -0.018(-0.047,0.011) | 0.2137 |  | 396 | -0.027(-0.056,0.001) | 0.0579 |
| 6-12mo | q4 | 81 | -0.005(-0.052,0.042) | 0.8373 |  | 79 | -0.022(-0.069,0.024) | 0.3469 |
| 12-18mo | q1 | 349 | ref | ref |  | 336 | ref | ref |
| 12-18mo | IQR | 404 | -0.000(-0.044,0.044) | 0.9853 |  | 388 | 0.010(-0.032,0.053) | 0.6397 |
| 12-18mo | q4 | 71 | 0.005(-0.071,0.082) | 0.8931 |  | 70 | 0.012(-0.052,0.076) | 0.7134 |
|  |  | **Tryptophan** | | | | | | |
| Interval | Quantile | Unadjusted | | |  | Adjusted | | |
|  |  | N | β (95%CI) | p-value |  | N | β (95%CI) | p-value |
| 1-3mo | q1 | 56 | ref | ref |  | 52 | ref | ref |
| 1-3mo | IQR | 257 | 0.035(-0.212,0.282) | 0.7815 |  | 242 | 0.103(-0.161,0.368) | 0.4447 |
| 1-3mo | q4 | 216 | 0.031(-0.211,0.274) | 0.7992 |  | 201 | 0.062(-0.194,0.319) | 0.634 |
| 3-6mo | q1 | 85 | ref | ref |  | 85 | ref | ref |
| 3-6mo | IQR | 364 | 0.073(-0.067,0.212) | 0.3068 |  | 363 | 0.068(-0.075,0.211) | 0.3517 |
| 3-6mo | q4 | 209 | 0.043(-0.091,0.177) | 0.5282 |  | 209 | 0.032(-0.101,0.165) | 0.6353 |
| 6-12mo | q1 | 212 | ref | ref |  | 174 | ref | ref |
| 6-12mo | IQR | 388 | -0.006(-0.038,0.025) | 0.6998 |  | 339 | -0.012(-0.044,0.020) | 0.4634 |
| 6-12mo | q4 | 145 | -0.021(-0.055,0.013) | 0.2317 |  | 126 | -0.026(-0.060,0.009) | 0.1469 |
| 12-18mo | q1 | 351 | ref | ref |  | 341 | ref | ref |
| 12-18mo | IQR | 368 | -0.017(-0.062,0.029) | 0.4709 |  | 354 | -0.013(-0.058,0.032) | 0.5756 |
| 12-18mo | q4 | 105 | -0.000(-0.060,0.059) | 0.9918 |  | 99 | 0.010(-0.044,0.065) | 0.717 |
|  |  | **Insulin-like growth factor 1** | | | | | | |
| Interval | Quantile | Unadjusted | | |  | Adjusted | | |
|  |  | N | β (95%CI) | p-value |  | N | β (95%CI) | p-value |
| 1-3mo | q1 | 94 | ref | ref |  | 90 | ref | ref |
| 1-3mo | IQR | 184 | 0.083(-0.106,0.271) | 0.3893 |  | 172 | 0.121(-0.062,0.303) | 0.1957 |
| 1-3mo | q4 | 276 | 0.128(-0.055,0.311) | 0.1695 |  | 256 | 0.190(0.011,0.370) | 0.0378 |
| 3-6mo | q1 | 131 | ref | ref |  | 130 | ref | ref |
| 3-6mo | IQR | 357 | -0.064(-0.192,0.065) | 0.3319 |  | 357 | -0.055(-0.174,0.065) | 0.3703 |
| 3-6mo | q4 | 205 | -0.082(-0.184,0.021) | 0.1177 |  | 205 | -0.102(-0.196,-0.008) | 0.0333 |
| 6-12mo | q1 | 200 | ref | ref |  | 170 | ref | ref |
| 6-12mo | IQR | 461 | 0.013(-0.018,0.044) | 0.4072 |  | 396 | 0.006(-0.025,0.038) | 0.6881 |
| 6-12mo | q4 | 136 | 0.045(0.013,0.078) | 0.006 |  | 114 | 0.029(-0.005,0.062) | 0.0932 |
| 12-18mo | q1 | 269 | ref | ref |  | 235 | ref | ref |
| 12-18mo | IQR | 488 | 0.037(0.000,0.074) | 0.0495 |  | 425 | 0.034(-0.012,0.080) | 0.1434 |
| 12-18mo | q4 | 125 | 0.012(-0.014,0.038) | 0.3703 |  | 102 | 0.011(-0.027,0.049) | 0.5711 |

**Supplementary Table 11.** Mean change in LAZ (SD/month) for children with by biomarker quantile.

|  |  | **Intestinal fatty acid binding protein** | | | | | | |
| --- | --- | --- | --- | --- | --- | --- | --- | --- |
| Interval | Quantile | Unadjusted | | |  | Adjusted | | |
|  |  | N | β (95%CI) | p-value |  | N | β (95%CI) | p-value |
| 1-3mo | < Median | 180 | ref | ref |  | 170 | ref | ref |
| 1-3mo | ≥ Median | 377 | -0.007(-0.126,0.112) | 0.9103 |  | 351 | 0.019(-0.125,0.163) | 0.7919 |
| 3-6mo | < Median | 473 | ref | ref |  | 472 | ref | ref |
| 3-6mo | ≥ Median | 220 | -0.011(-0.109,0.088) | 0.8322 |  | 220 | -0.024(-0.112,0.064) | 0.5955 |
| 6-12mo | < Median | 526 | ref | ref |  | 439 | ref | ref |
| 6-12mo | ≥ Median | 271 | -0.005(-0.032,0.021) | 0.6913 |  | 241 | 0.001(-0.026,0.027) | 0.9494 |
| 12-18mo | < Median | 392 | ref | ref |  | 329 | ref | ref |
| 12-18mo | ≥ Median | 490 | 0.002(-0.040,0.044) | 0.9275 |  | 433 | 0.014(-0.026,0.055) | 0.489 |
|  |  | **Regenerating protein 1-β** | | | | | | |
| Interval | Quantile | Unadjusted | | |  | Adjusted | | |
|  |  | N | β (95%CI) | p-value |  | N | β (95%CI) | p-value |
| 1-3mo | < Median | 315 | ref | ref |  | 313 | ref | ref |
| 1-3mo | ≥ Median | 54 | -0.027(-0.185,0.132) | 0.7396 |  | 52 | -0.018(-0.184,0.147) | 0.8277 |
| 3-6mo | < Median | 296 | ref | ref |  | 293 | ref | ref |
| 3-6mo | ≥ Median | 142 | -0.014(-0.097,0.069) | 0.7421 |  | 140 | -0.039(-0.123,0.045) | 0.3641 |
| 6-12mo | < Median | 251 | ref | ref |  | 226 | ref | ref |
| 6-12mo | ≥ Median | 471 | -0.017(-0.045,0.010) | 0.2117 |  | 416 | -0.014(-0.042,0.014) | 0.3324 |
| 12-18mo | < Median | 303 | ref | ref |  | 269 | ref | ref |
| 12-18mo | ≥ Median | 566 | -0.010(-0.050,0.031) | 0.6445 |  | 486 | 0.010(-0.025,0.044) | 0.5719 |
|  |  | **Citrulline** | | | | | | |
| Interval | Quantile | Unadjusted | | |  | Adjusted | | |
|  |  | N | β (95%CI) | p-value |  | N | β (95%CI) | p-value |
| 1-3mo | < Median | 286 | ref | ref |  | 269 | ref | ref |
| 1-3mo | ≥ Median | 243 | 0.034(-0.088,0.156) | 0.584 |  | 227 | 0.053(-0.061,0.168) | 0.362 |
| 3-6mo | < Median | 400 | ref | ref |  | 399 | ref | ref |
| 3-6mo | ≥ Median | 258 | 0.032(-0.066,0.130) | 0.5263 |  | 258 | 0.021(-0.096,0.138) | 0.7246 |
| 6-12mo | < Median | 397 | ref | ref |  | 346 | ref | ref |
| 6-12mo | ≥ Median | 348 | 0.004(-0.023,0.031) | 0.7629 |  | 293 | -0.011(-0.038,0.016) | 0.4259 |
| 12-18mo | < Median | 296 | ref | ref |  | 287 | ref | ref |
| 12-18mo | ≥ Median | 528 | -0.016(-0.063,0.030) | 0.4899 |  | 507 | -0.034(-0.087,0.018) | 0.2003 |
|  |  | **Alpha-1 Antitrypsin** | | | | | | |
| Interval | Quantile | Unadjusted | | |  | Adjusted | | |
|  |  | N | β (95%CI) | p-value |  | N | β (95%CI) | p-value |
| 1-3mo | < Median | 175 | ref | ref |  | 174 | ref | ref |
| 1-3mo | ≥ Median | 222 | 0.027(-0.118,0.173) | 0.7141 |  | 218 | 0.017(-0.121,0.155) | 0.808 |
| 3-6mo | < Median | 207 | ref | ref |  | 189 | ref | ref |
| 3-6mo | ≥ Median | 265 | -0.003(-0.080,0.075) | 0.9492 |  | 255 | 0.006(-0.070,0.083) | 0.8688 |
| 6-12mo | < Median | 323 | ref | ref |  | 300 | ref | ref |
| 6-12mo | ≥ Median | 421 | -0.025(-0.050,0.001) | 0.0576 |  | 389 | -0.027(-0.051,-0.002) | 0.0344 |
| 12-18mo | < Median | 442 | ref | ref |  | 383 | ref | ref |
| 12-18mo | ≥ Median | 441 | 0.033(-0.006,0.071) | 0.0973 |  | 384 | 0.043(0.005,0.081) | 0.0258 |
|  |  | **Lactulose:mannitol ratio** | | | | | | |
| Interval | Quantile | Unadjusted | | |  | Adjusted | | |
|  |  | N | β (95%CI) | p-value |  | N | β (95%CI) | p-value |
| 1-3mo | < Median | NA | NA | NA |  | NA | NA | NA |
| 1-3mo | ≥ Median | NA | NA | NA |  | NA | NA | NA |
| 3-6mo | < Median | 177 | ref | ref |  | 168 | ref | ref |
| 3-6mo | ≥ Median | 227 | -0.030(-0.117,0.057) | 0.4995 |  | 218 | -0.059(-0.142,0.025) | 0.1676 |
| 6-12mo | < Median | 246 | ref | ref |  | 222 | ref | ref |
| 6-12mo | ≥ Median | 263 | -0.022(-0.056,0.012) | 0.1985 |  | 242 | -0.026(-0.060,0.009) | 0.1497 |
| 12-18mo | < Median | 232 | ref | ref |  | 225 | ref | ref |
| 12-18mo | ≥ Median | 306 | 0.022(-0.025,0.070) | 0.3613 |  | 290 | 0.030(-0.024,0.083) | 0.2777 |
|  |  | **Lactulose Excretion Fracion** | | | | | | |
| Interval | Quantile | Unadjusted | | |  | Adjusted | | |
|  |  | N | β (95%CI) | p-value |  | N | β (95%CI) | p-value |
| 1-3mo | < Median | NA | NA | NA |  | NA | NA | NA |
| 1-3mo | ≥ Median | NA | NA | NA |  | NA | NA | NA |
| 3-6mo | < Median | 220 | ref | ref |  | 209 | ref | ref |
| 3-6mo | ≥ Median | 187 | 0.013(-0.076,0.102) | 0.7737 |  | 180 | 0.017(-0.064,0.098) | 0.6814 |
| 6-12mo | < Median | 328 | ref | ref |  | 298 | ref | ref |
| 6-12mo | ≥ Median | 182 | 0.023(-0.013,0.059) | 0.2138 |  | 167 | 0.032(-0.004,0.069) | 0.0798 |
| 12-18mo | < Median | 354 | ref | ref |  | 334 | ref | ref |
| 12-18mo | ≥ Median | 201 | 0.023(-0.039,0.085) | 0.4707 |  | 197 | 0.004(-0.042,0.050) | 0.8725 |
|  |  | **Mannitol excretion fraction** | | | | | | |
| Interval | Quantile | Unadjusted | | |  | Adjusted | | |
|  |  | N | β (95%CI) | p-value |  | N | β (95%CI) | p-value |
| 1-3mo | < Median | NA | NA | NA |  | NA | NA | NA |
| 1-3mo | ≥ Median | NA | NA | NA |  | NA | NA | NA |
| 3-6mo | < Median | 229 | ref | ref |  | 219 | ref | ref |
| 3-6mo | ≥ Median | 175 | -0.048(-0.137,0.041) | 0.2901 |  | 167 | -0.025(-0.106,0.057) | 0.5542 |
| 6-12mo | < Median | 321 | ref | ref |  | 291 | ref | ref |
| 6-12mo | ≥ Median | 188 | 0.018(-0.017,0.054) | 0.3127 |  | 173 | 0.026(-0.009,0.062) | 0.1442 |
| 12-18mo | < Median | 350 | ref | ref |  | 331 | ref | ref |
| 12-18mo | ≥ Median | 189 | 0.004(-0.043,0.051) | 0.8646 |  | 185 | -0.029(-0.109,0.051) | 0.4822 |
|  |  | **soluble CD14** | | | | | | |
| Interval | Quantile | Unadjusted | | |  | Adjusted | | |
|  |  | N | β (95%CI) | p-value |  | N | β (95%CI) | p-value |
| 1-3mo | < Median | 447 | ref | ref |  | 416 | ref | ref |
| 1-3mo | ≥ Median | 109 | -0.028(-0.191,0.135) | 0.7349 |  | 104 | -0.032(-0.208,0.143) | 0.7177 |
| 3-6mo | < Median | 408 | ref | ref |  | 407 | ref | ref |
| 3-6mo | ≥ Median | 286 | -0.080(-0.176,0.017) | 0.1046 |  | 286 | -0.093(-0.203,0.017) | 0.099 |
| 6-12mo | < Median | 391 | ref | ref |  | 333 | ref | ref |
| 6-12mo | ≥ Median | 406 | -0.005(-0.030,0.021) | 0.7217 |  | 347 | -0.005(-0.030,0.021) | 0.7148 |
| 12-18mo | < Median | 359 | ref | ref |  | 306 | ref | ref |
| 12-18mo | ≥ Median | 523 | 0.002(-0.039,0.043) | 0.9225 |  | 456 | -0.013(-0.059,0.033) | 0.5838 |
|  |  | **Myeloperoxidase** | | | | | | |
|  |  | Unadjusted | | |  | Adjusted | | |
| Interval | Quantile | N | β (95%CI) | p-value |  | N | β (95%CI) | p-value |
| 1-3mo | < Median | 165 | ref | ref |  | 163 | ref | ref |
| 1-3mo | ≥ Median | 237 | -0.022(-0.176,0.131) | 0.7772 |  | 234 | -0.019(-0.176,0.138) | 0.8102 |
| 3-6mo | < Median | 135 | ref | ref |  | 133 | ref | ref |
| 3-6mo | ≥ Median | 345 | -0.063(-0.159,0.034) | 0.2012 |  | 342 | -0.044(-0.140,0.052) | 0.3719 |
| 6-12mo | < Median | 220 | ref | ref |  | 205 | ref | ref |
| 6-12mo | ≥ Median | 525 | -0.026(-0.054,0.003) | 0.0746 |  | 485 | -0.027(-0.054,-0.001) | 0.0443 |
| 12-18mo | < Median | 502 | ref | ref |  | 478 | ref | ref |
| 12-18mo | ≥ Median | 380 | 0.024(-0.020,0.067) | 0.2842 |  | 366 | 0.032(-0.016,0.080) | 0.1953 |
|  |  | **Neopterin** | | | | | | |
| Interval | Quantile | Unadjusted | | |  | Adjusted | | |
|  |  | N | β (95%CI) | p-value |  | N | β (95%CI) | p-value |
| 1-3mo | < Median | 208 | ref | ref |  | 204 | ref | ref |
| 1-3mo | ≥ Median | 186 | -0.082(-0.225,0.062) | 0.2652 |  | 185 | -0.096(-0.248,0.055) | 0.2133 |
| 3-6mo | < Median | 137 | ref | ref |  | 135 | ref | ref |
| 3-6mo | ≥ Median | 329 | -0.022(-0.106,0.061) | 0.6013 |  | 326 | -0.016(-0.096,0.063) | 0.686 |
| 6-12mo | < Median | 187 | ref | ref |  | 166 | ref | ref |
| 6-12mo | ≥ Median | 550 | -0.001(-0.030,0.028) | 0.9411 |  | 490 | -0.008(-0.038,0.021) | 0.5723 |
| 12-18mo | < Median | 435 | ref | ref |  | 384 | ref | ref |
| 12-18mo | ≥ Median | 441 | -0.020(-0.059,0.019) | 0.3157 |  | 377 | 0.006(-0.025,0.037) | 0.7234 |
|  |  | **EE Score** | | | | | | |
| Interval | Quantile | Unadjusted | | |  | Adjusted | | |
|  |  | N | β (95%CI) | p-value |  | N | β (95%CI) | p-value |
| 1-3mo | < Median | 134 | ref | ref |  | 133 | ref | ref |
| 1-3mo | ≥ Median | 255 | -0.047(-0.200,0.107) | 0.549 |  | 251 | -0.018(-0.178,0.141) | 0.8235 |
| 3-6mo | < Median | 103 | ref | ref |  | 102 | ref | ref |
| 3-6mo | ≥ Median | 357 | -0.031(-0.121,0.059) | 0.4985 |  | 353 | -0.008(-0.095,0.079) | 0.8547 |
| 6-12mo | < Median | 176 | ref | ref |  | 165 | ref | ref |
| 6-12mo | ≥ Median | 560 | -0.027(-0.057,0.004) | 0.0879 |  | 516 | -0.028(-0.057,0.001) | 0.0575 |
| 12-18mo | < Median | 381 | ref | ref |  | 335 | ref | ref |
| 12-18mo | ≥ Median | 492 | 0.031(-0.005,0.067) | 0.0956 |  | 425 | 0.019(-0.019,0.057) | 0.3285 |
|  |  | **C-reactive Protein** | | | | | | |
| Interval | Quantile | Unadjusted | | |  | Adjusted | | |
|  |  | N | β (95%CI) | p-value |  | N | β (95%CI) | p-value |
| 1-3mo | < Median | 349 | ref | ref |  | 323 | ref | ref |
| 1-3mo | ≥ Median | 207 | -0.010(-0.133,0.113) | 0.8687 |  | 197 | 0.015(-0.126,0.156) | 0.8319 |
| 3-6mo | < Median | 354 | ref | ref |  | 354 | ref | ref |
| 3-6mo | ≥ Median | 340 | -0.002(-0.105,0.102) | 0.9749 |  | 339 | -0.007(-0.114,0.101) | 0.9058 |
| 6-12mo | < Median | 371 | ref | ref |  | 318 | ref | ref |
| 6-12mo | ≥ Median | 426 | 0.020(-0.006,0.045) | 0.1293 |  | 362 | 0.015(-0.011,0.041) | 0.2544 |
| 12-18mo | < Median | 402 | ref | ref |  | 346 | ref | ref |
| 12-18mo | ≥ Median | 480 | -0.023(-0.065,0.018) | 0.2646 |  | 416 | -0.023(-0.072,0.025) | 0.3443 |
|  |  | **Kynurenine:tryptophan ratio** | | | | | | |
| Interval | Quantile | Unadjusted | | |  | Adjusted | | |
|  |  | N | β (95%CI) | p-value |  | N | β (95%CI) | p-value |
| 1-3mo | < Median | 137 | ref | ref |  | 132 | ref | ref |
| 1-3mo | ≥ Median | 336 | 0.030(-0.111,0.170) | 0.6801 |  | 313 | 0.027(-0.115,0.168) | 0.7143 |
| 3-6mo | < Median | 322 | ref | ref |  | 321 | ref | ref |
| 3-6mo | ≥ Median | 290 | 0.102(-0.017,0.222) | 0.0926 |  | 290 | 0.132(-0.026,0.291) | 0.1022 |
| 6-12mo | < Median | 427 | ref | ref |  | 394 | ref | ref |
| 6-12mo | ≥ Median | 298 | -0.009(-0.038,0.020) | 0.5503 |  | 271 | -0.015(-0.043,0.014) | 0.3047 |
| 12-18mo | < Median | 439 | ref | ref |  | 420 | ref | ref |
| 12-18mo | ≥ Median | 362 | 0.012(-0.032,0.056) | 0.5965 |  | 352 | -0.006(-0.047,0.035) | 0.7824 |
|  |  | **Kynurenine** | | | | | | |
| Interval | Quantile | Unadjusted | | |  | Adjusted | | |
|  |  | N | β (95%CI) | p-value |  | N | β (95%CI) | p-value |
| 1-3mo | < Median | 96 | ref | ref |  | 90 | ref | ref |
| 1-3mo | ≥ Median | 433 | 0.110(-0.052,0.272) | 0.1821 |  | 406 | 0.108(-0.060,0.275) | 0.2079 |
| 3-6mo | < Median | 262 | ref | ref |  | 261 | ref | ref |
| 3-6mo | ≥ Median | 396 | 0.053(-0.047,0.153) | 0.3013 |  | 396 | 0.074(-0.072,0.219) | 0.3217 |
| 6-12mo | < Median | 479 | ref | ref |  | 436 | ref | ref |
| 6-12mo | ≥ Median | 266 | -0.007(-0.036,0.021) | 0.6098 |  | 247 | -0.014(-0.043,0.014) | 0.3252 |
| 12-18mo | < Median | 573 | ref | ref |  | 553 | ref | ref |
| 12-18mo | ≥ Median | 251 | -0.014(-0.050,0.022) | 0.4493 |  | 241 | -0.003(-0.033,0.027) | 0.8386 |
|  |  | **Tryptophan** | | | | | | |
| Interval | Quantile | Unadjusted | | |  | Adjusted | | |
|  |  | N | β (95%CI) | p-value |  | N | β (95%CI) | p-value |
| 1-3mo | < Median | 162 | ref | ref |  | 155 | ref | ref |
| 1-3mo | ≥ Median | 367 | -0.080(-0.233,0.073) | 0.3035 |  | 341 | -0.073(-0.229,0.082) | 0.3535 |
| 3-6mo | < Median | 255 | ref | ref |  | 255 | ref | ref |
| 3-6mo | ≥ Median | 403 | -0.052(-0.180,0.076) | 0.4288 |  | 402 | -0.068(-0.198,0.063) | 0.3114 |
| 6-12mo | < Median | 424 | ref | ref |  | 382 | ref | ref |
| 6-12mo | ≥ Median | 321 | -0.006(-0.032,0.021) | 0.6674 |  | 301 | -0.012(-0.036,0.012) | 0.3424 |
| 12-18mo | < Median | 552 | ref | ref |  | 537 | ref | ref |
| 12-18mo | ≥ Median | 272 | 0.011(-0.035,0.057) | 0.6422 |  | 257 | 0.017(-0.028,0.063) | 0.4594 |
|  |  | **Insulin-like growth factor 1** | | | | | | |
| Interval | Quantile | Unadjusted | | |  | Adjusted | | |
|  |  | N | β (95%CI) | p-value |  | N | β (95%CI) | p-value |
| 1-3mo | < Median | 164 | ref | ref |  | 157 | ref | ref |
| 1-3mo | ≥ Median | 390 | 0.114(-0.025,0.253) | 0.1067 |  | 361 | 0.164(0.022,0.305) | 0.0232 |
| 3-6mo | < Median | 291 | ref | ref |  | 290 | ref | ref |
| 3-6mo | ≥ Median | 402 | -0.046(-0.166,0.075) | 0.4586 |  | 402 | -0.053(-0.160,0.055) | 0.3374 |
| 6-12mo | < Median | 459 | ref | ref |  | 394 | ref | ref |
| 6-12mo | ≥ Median | 338 | 0.002(-0.023,0.027) | 0.8819 |  | 286 | -0.013(-0.038,0.012) | 0.3147 |
| 12-18mo | < Median | 524 | ref | ref |  | 462 | ref | ref |
| 12-18mo | ≥ Median | 358 | -0.003(-0.041,0.036) | 0.8862 |  | 300 | -0.007(-0.031,0.017) | 0.5737 |

**Supplementary Table 12**. Cumulative odds ratio of attaining the higher or highest compared to the lowest LAZ group (lowest: LAZ<−2, higher: −2 ≤ LAZ < −1, highest ≥ −1) at 18 mo for the highest vs the lowest biomarker quartile Cumulative Odds Ratios of LAZ< -2, -2≤LAZ<-1, LAZ≥-1

| Marker | Quartile | N | β (95%CI) | p-value |
| --- | --- | --- | --- | --- |
| Intestinal fatty acid binding protein | q1 | 171 | ref | ref |
|  | q4 | 344 | 0.912(0.651,1.277) | 0.5915 |
| Citrulline | q1 | 134 | ref | ref |
|  | q4 | 319 | 0.777(0.535,1.127) | 0.184 |
| Regenerating protein-1 β | q1 | 293 | ref | ref |
|  | q4 | 281 | 1.307(0.966,1.769) | 0.0825 |
| Alpah-1 Antitrypsin | q1 | 334 | ref | ref |
|  | q4 | 134 | 1.565(1.080,2.276) | **0.0184** |
| Lactulose:mannitol ratio | q1 | 229 | ref | ref |
|  | q4 | 79 | 0.986(0.616,1.579) | 0.9541 |
| Lactulose Excretion Fraction | q1 | 52 | ref | ref |
|  | q4 | 345 | 0.854(0.496,1.467) | 0.5672 |
| Mannitol Excretion Fraction | q1 | 41 | ref | ref |
|  | q4 | 346 | 0.905(0.497,1.644) | 0.7426 |
| soluble CD14 | q1 | 77 | ref | ref |
|  | q4 | 369 | 1.110(0.702,1.754) | 0.6556 |
| Myeloperoxidase | q1 | 474 | ref | ref |
|  | q4 | 52 | 1.307(0.776,2.207) | 0.3145 |
| Neopterin | q1 | 540 | ref | ref |
|  | q4 | 83 | 1.237(0.817,1.879) | 0.3152 |
| EE Score | q1 | 357 | ref | ref |
|  | q4 | 67 | 1.028(0.640,1.653) | 0.9084 |
| C-reactive protein | q1 | 287 | ref | ref |
|  | q4 | 267 | 1.140(0.838,1.551) | 0.4042 |
| Kynurenine:tryptophan ratio | q1 | 240 | ref | ref |
|  | q4 | 181 | 0.628(0.439,0.898) | **0.011** |
| Kynurenine | q1 | 351 | ref | ref |
|  | q4 | 72 | 0.909(0.571,1.447) | 0.6877 |
| Tryptophan | q1 | 352 | ref | ref |
|  | q4 | 106 | 1.373(0.922,2.050) | 0.1196 |
| Insulin-like growth factor 1 | q1 | 299 | ref | ref |
|  | q4 | 218 | 1.975(1.424,2.746) | **<0.001** |

**Supplementary Table 13.** Mean change in LAZ at age 18 months per 1 unit increase in mean age and breastfeeding detrended biomarker concentration

| Marker | Complete | B (95%CI) | p-value |
| --- | --- | --- | --- |
| Intestinal fatty acid binding protein | 885 | 0.753(0.557,1.019) | 0.0659 |
| Citrulline | 820 | 0.752(0.527,1.070) | 0.1133 |
| Regenerating protein 1-β | 830 | 1.187(1.053,1.338) | 0.0051 |
| Alpha-1 antitrypsin | 858 | 1.000(0.861,1.164) | 0.9988 |
| Lactulose:mannitol ratio | 569 | 1.052(0.868,1.276) | 0.6042 |
| Lactulose excretion fraction | 571 | 0.971(0.839,1.122) | 0.6883 |
| Mannitol excretion fraction | 570 | 0.972(0.867,1.089) | 0.6207 |
| soluble cd14 | 885 | 1.101(0.761,1.594) | 0.6108 |
| Myeloperoxidase | 858 | 0.999(0.975,1.024) | 0.9374 |
| Neopterin | 857 | 1.073(0.949,1.214) | 0.2627 |
| EE Score | 857 | 1.099(0.995,1.213) | 0.0623 |
| C-reactive protein | 885 | 1.054(0.969,1.146) | 0.2209 |
| Kynurenine:tryptophan ratio | 797 | 0.754(0.487,1.168) | 0.2069 |
| Kynurenine | 820 | 1.158(0.786,1.709) | 0.4586 |
| Tryptophan | 820 | 1.234(0.852,1.797) | 0.2671 |
| Insulin-like growth factor 1 | 885 | 2.311(1.764,3.037) | <0.001 |

**Supplementary Table 14**. Mean change in WHZ (sd/month) per 1 sd increase in biomarker concentration at the start of each follow-up interval

| **Intestinal fatty acid binding protein** | | | | | | | |
| --- | --- | --- | --- | --- | --- | --- | --- |
|  | Unadjusted | | |  | Adjusted | | |
| Interval | N | β (95%CI) | p-value |  | N | β (95%CI) | p-value |
| 1-3mo | 550 | 0.027(-0.038,0.093) | 0.414 |  | 493 | 0.024(-0.046,0.095) | 0.496 |
| 3-6mo | 692 | 0.006(-0.042,0.055) | 0.796 |  | 660 | 0.007(-0.039,0.054) | 0.758 |
| 6-12mo | 797 | -0.010(-0.054,0.035) | 0.671 |  | 705 | -0.007(-0.059,0.045) | 0.787 |
| 12-18mo | 876 | -0.002(-0.016,0.012) | 0.766 |  | 828 | 0.000(-0.017,0.018) | 0.962 |
| **Citrulline** | | | | | | | |
| Interval | Unadjusted | | |  | Adjusted | | |
|  | N | β (95%CI) | p-value |  | N | β (95%CI) | p-value |
| 1-3mo | 523 | -0.037(-0.127,0.053) | 0.421 |  | 467 | -0.049(-0.146,0.047) | 0.316 |
| 3-6mo | 657 | 0.006(-0.039,0.052) | 0.786 |  | 626 | 0.003(-0.047,0.053) | 0.912 |
| 6-12mo | 745 | 0.001(-0.014,0.015) | 0.939 |  | 659 | -0.000(-0.015,0.014) | 0.970 |
| 12-18mo | 818 | -0.014(-0.028,0.000) | 0.054 |  | 773 | -0.018(-0.034,-0.001) | **0.034** |
| **Regenerating Protein 1-β** | | | | | | | |
|  | Unadjusted | | |  | Adjusted | | |
| Interval | N | β (95%CI) | p-value |  | N | β (95%CI) | p-value |
| 1-3mo | 364 | -0.022(-0.124,0.080) | 0.668 |  | 324 | -0.038(-0.153,0.077) | 0.516 |
| 3-6mo | 438 | 0.039(-0.008,0.085) | 0.103 |  | 413 | 0.047(-0.005,0.099) | 0.077 |
| 6-12mo | 722 | 0.008(-0.009,0.024) | 0.348 |  | 638 | 0.002(-0.013,0.017) | 0.753 |
| 12-18mo | 863 | 0.002(-0.009,0.014) | 0.699 |  | 863 | 0.005(-0.006,0.016) | 0.399 |
| **Alpha-1 Antitrypsin** | | | | | | | |
|  | Unadjusted | | |  | Adjusted | | |
| Interval | N | β (95%CI) | p-value |  | N | β (95%CI) | p-value |
| 1-3mo | 392 | -0.050(-0.134,0.035) | 0.248 |  | 347 | -0.058(-0.146,0.031) | 0.205 |
| 3-6mo | 472 | -0.016(-0.056,0.025) | 0.451 |  | 443 | -0.025(-0.068,0.018) | 0.254 |
| 6-12mo | 744 | -0.001(-0.016,0.014) | 0.866 |  | 650 | -0.000(-0.024,0.024) | 0.997 |
| 12-18mo | 877 | -0.007(-0.019,0.004) | 0.196 |  | 877 | -0.007(-0.018,0.005) | 0.242 |
| **Lactulose:mannitol Ratio** | | | | | | | |
|  | Unadjusted | | |  | Adjusted | | |
| Interval | N | β (95%CI) | p-value |  | N | β (95%CI) | p-value |
| 3-6mo | 404 | -0.067(-0.147,0.013) | 0.099 |  | 357 | -0.078(-0.149,-0.008) | **0.030** |
| 6-12mo | 509 | 0.003(-0.033,0.038) | 0.888 |  | 499 | 0.004(-0.029,0.037) | 0.815 |
| 12-18mo | 535 | -0.006(-0.033,0.021) | 0.673 |  | 503 | -0.010(-0.047,0.027) | 0.602 |
| **Lactulose Excretion Fraction** | | | | | | | |
|  | Unadjusted | | |  | Adjusted | | |
| Interval | N | β (95%CI) | p-value |  | N | β (95%CI) | p-value |
| 3-6mo | 407 | 0.149(-0.523,0.820) | 0.664 |  | 360 | 0.157(-0.547,0.862) | 0.662 |
| 6-12mo | 510 | 0.003(-0.015,0.020) | 0.753 |  | 501 | 0.002(-0.016,0.020) | 0.801 |
| 12-18mo | 552 | 0.007(-0.000,0.014) | 0.052 |  | 517 | 0.004(-0.005,0.012) | 0.416 |
| **Mannitol Excretion Fraction** | | | | | | | |
|  | Unadjusted | | |  | Adjusted | | |
| Interval | N | β (95%CI) | p-value |  | N | β (95%CI) | p-value |
| 3-6mo | 404 | 0.019(-0.088,0.126) | 0.727 |  | 357 | 0.020(-0.088,0.128) | 0.717 |
| 6-12mo | 509 | -0.001(-0.018,0.015) | 0.862 |  | 500 | -0.001(-0.017,0.016) | 0.943 |
| 12-18mo | 536 | 0.001(-0.016,0.018) | 0.912 |  | 504 | -0.001(-0.018,0.016) | 0.948 |
| **soluble CD14** | | | | | | | |
|  | Unadjusted | | |  | Adjusted | | |
| Interval | N | β (95%CI) | p-value |  | N | β (95%CI) | p-value |
| 1-3mo | 549 | 0.045(-0.072,0.162) | 0.453 |  | 492 | 0.068(-0.064,0.201) | 0.313 |
| 3-6mo | 693 | 0.018(-0.037,0.072) | 0.524 |  | 661 | 0.013(-0.046,0.073) | 0.665 |
| 6-12mo | 797 | -0.002(-0.016,0.012) | 0.790 |  | 705 | -0.001(-0.016,0.015) | 0.924 |
| 12-18mo | 876 | -0.005(-0.018,0.009) | 0.491 |  | 828 | -0.002(-0.016,0.011) | 0.730 |
| **Myeloperoxidase** | | | | | | | |
|  | Unadjusted | | |  | Adjusted | | |
| Interval | N | β (95%CI) | p-value |  | N | β (95%CI) | p-value |
| 1-3mo | 397 | -0.027(-0.102,0.048) | 0.480 |  | 351 | -0.025(-0.143,0.093) | 0.677 |
| 3-6mo | 480 | -0.004(-0.050,0.041) | 0.847 |  | 450 | -0.005(-0.053,0.042) | 0.825 |
| 6-12mo | 745 | -0.010(-0.025,0.005) | 0.172 |  | 651 | -0.011(-0.027,0.006) | 0.209 |
| 12-18mo | 876 | 0.012(-0.006,0.030) | 0.191 |  | 876 | 0.012(-0.005,0.029) | 0.176 |
| **Neopterin** | | | | | | | |
|  | Unadjusted | | |  | Adjusted | | |
| Interval | N | β (95%CI) | p-value |  | N | β (95%CI) | p-value |
| 1-3mo | 389 | -0.005(-0.179,0.169) | 0.957 |  | 343 | 0.006(-0.152,0.163) | 0.942 |
| 3-6mo | 466 | 0.013(-0.043,0.068) | 0.656 |  | 437 | 0.019(-0.039,0.076) | 0.524 |
| 6-12mo | 737 | -0.002(-0.013,0.010) | 0.770 |  | 645 | -0.003(-0.014,0.009) | 0.641 |
| 12-18mo | 870 | -0.005(-0.016,0.006) | 0.407 |  | 870 | -0.002(-0.012,0.008) | 0.681 |
| **EE Score** | | | | | | | |
|  | Unadjusted | | |  | Adjusted | | |
| Interval | N | β (95%CI) | p-value |  | N | β (95%CI) | p-value |
| 1-3mo | 384 | -0.044(-0.147,0.060) | 0.406 |  | 339 | -0.042(-0.163,0.078) | 0.490 |
| 3-6mo | 460 | 0.004(-0.043,0.052) | 0.865 |  | 432 | 0.002(-0.048,0.052) | 0.939 |
| 6-12mo | 736 | 0.013(-0.005,0.031) | 0.157 |  | 644 | 0.012(-0.006,0.030) | 0.178 |
| 12-18mo | 867 | 0.001(-0.011,0.013) | 0.860 |  | 867 | 0.002(-0.010,0.015) | 0.730 |
| **C-reactive Protein** | | | | | | | |
|  | Unadjusted | | |  | Adjusted | | |
| Interval | N | β (95%CI) | p-value |  | N | β (95%CI) | p-value |
| 1-3mo | 549 | -0.035(-0.098,0.028) | 0.273 |  | 492 | -0.034(-0.098,0.030) | 0.294 |
| 3-6mo | 693 | -0.027(-0.073,0.020) | 0.259 |  | 661 | -0.025(-0.071,0.021) | 0.280 |
| 6-12mo | 797 | -0.006(-0.028,0.017) | 0.621 |  | 705 | -0.006(-0.031,0.020) | 0.652 |
| 12-18mo | 876 | 0.015(-0.001,0.030) | 0.059 |  | 810 | 0.016(0.000,0.032) | **0.046** |
| **Kynurenine:Tryptophan Ratio** | | | | | | | |
|  | Unadjusted | | |  | Adjusted | | |
| Interval | N | β (95%CI) | p-value |  | N | β (95%CI) | p-value |
| 1-3mo | 467 | -0.011(-0.087,0.064) | 0.768 |  | 420 | -0.015(-0.095,0.064) | 0.705 |
| 3-6mo | 611 | 0.006(-0.035,0.048) | 0.764 |  | 583 | 0.006(-0.035,0.048) | 0.757 |
| 6-12mo | 725 | 0.007(-0.012,0.025) | 0.476 |  | 643 | 0.001(-0.019,0.022) | 0.905 |
| 12-18mo | 795 | 0.017(0.004,0.030) | 0.008 |  | 751 | 0.017(0.003,0.030) | **0.014** |
| **Kynurenine** | | | | | | | |
|  | Unadjusted | | |  | Adjusted | | |
| Interval | N | β (95%CI) | p-value |  | N | β (95%CI) | p-value |
| 1-3mo | 523 | -0.023(-0.099,0.053) | 0.553 |  | 467 | -0.027(-0.110,0.057) | 0.533 |
| 3-6mo | 657 | 0.012(-0.048,0.073) | 0.688 |  | 626 | 0.014(-0.044,0.073) | 0.635 |
| 6-12mo | 745 | 0.011(-0.005,0.027) | 0.176 |  | 659 | 0.004(-0.012,0.020) | 0.612 |
| 12-18mo | 818 | -0.007(-0.020,0.006) | 0.303 |  | 773 | -0.005(-0.018,0.008) | 0.450 |
| **Tryptophan** | | | | | | | |
|  | Unadjusted | | |  | Adjusted | | |
| Interval | N | β (95%CI) | p-value |  | N | β (95%CI) | p-value |
| 1-3mo | 523 | -0.031(-0.116,0.054) | 0.476 |  | 467 | -0.039(-0.133,0.054) | 0.407 |
| 3-6mo | 657 | -0.019(-0.087,0.050) | 0.591 |  | 626 | -0.020(-0.091,0.051) | 0.587 |
| 6-12mo | 745 | -0.002(-0.017,0.012) | 0.755 |  | 659 | -0.005(-0.020,0.010) | 0.552 |
| 12-18mo | 818 | -0.017(-0.031,-0.004) | 0.013 |  | 773 | -0.017(-0.029,-0.004) | **0.010** |
| **Insulin-like growth factor-1** | | | | | | | |
|  | Unadjusted | | |  | Adjusted | | |
| Interval | N | β (95%CI) | p-value |  | N | β (95%CI) | p-value |
| 1-3mo | 547 | -0.072(-0.169,0.026) | 0.149 |  | 490 | -0.078(-0.188,0.031) | 0.162 |
| 3-6mo | 692 | -0.027(-0.070,0.017) | 0.230 |  | 660 | -0.028(-0.073,0.017) | 0.217 |
| 6-12mo | 797 | -0.007(-0.028,0.015) | 0.553 |  | 705 | 0.002(-0.014,0.019) | 0.769 |
| 12-18mo | 876 | -0.020(-0.031,-0.008) | 0.001 |  | 828 | -0.018(-0.028,-0.007) | **0.001** |

**Supplementary Table 15.** Mean change in weight (kg/month) per 1 sd increase in biomarker concentration at the start of each follow-up interval

| **Intestinal fatty acid binding protein** | | | | | | | |
| --- | --- | --- | --- | --- | --- | --- | --- |
|  | Unadjusted | | |  | Adjusted | | |
| Interval | N | β (95%CI) | p-value |  | N | β (95%CI) | p-value |
| 1-3mo | 553 | 0.050(0.020,0.080) | 0.001 |  | 496 | 0.039(0.008,0.071) | **0.014** |
| 3-6mo | 695 | 0.000(-0.030,0.031) | 0.982 |  | 663 | -0.002(-0.035,0.031) | 0.893 |
| 6-12mo | 797 | -0.000(-0.010,0.009) | 0.977 |  | 797 | 0.001(-0.010,0.011) | 0.902 |
| 12-18mo | 877 | -0.006(-0.020,0.009) | 0.435 |  | 877 | -0.003(-0.018,0.013) | 0.743 |
| **Citrulline** | | | | | | | |
| Interval | Unadjusted | | |  | Adjusted | | |
|  | N | β (95%CI) | p-value |  | N | β (95%CI) | p-value |
| 1-3mo | 526 | 0.012(-0.022,0.046) | 0.503 |  | 470 | 0.008(-0.028,0.043) | 0.667 |
| 3-6mo | 660 | 0.010(-0.017,0.037) | 0.480 |  | 629 | 0.010(-0.021,0.041) | 0.542 |
| 6-12mo | 745 | 0.004(-0.006,0.013) | 0.428 |  | 745 | 0.003(-0.005,0.012) | 0.442 |
| 12-18mo | 819 | -0.015(-0.036,0.005) | 0.145 |  | 774 | -0.024(-0.051,0.003) | 0.085 |
| **Regenerating Protein 1-β** | | | | | | | |
|  | Unadjusted | | |  | Adjusted | | |
| Interval | N | β (95%CI) | p-value |  | N | β (95%CI) | p-value |
| 1-3mo | 367 | -0.009(-0.040,0.021) | 0.539 |  | 235 | -0.015(-0.060,0.031) | 0.524 |
| 3-6mo | 441 | -0.005(-0.031,0.021) | 0.706 |  | 433 | 0.007(-0.019,0.033) | 0.588 |
| 6-12mo | 723 | -0.001(-0.011,0.009) | 0.857 |  | 643 | -0.005(-0.014,0.005) | 0.315 |
| 12-18mo | 864 | -0.002(-0.013,0.009) | 0.737 |  | 864 | 0.003(-0.007,0.013) | 0.552 |
| **Alpha-1 Antitrypsin** | | | | | | | |
|  | Unadjusted | | |  | Adjusted | | |
| Interval | N | β (95%CI) | p-value |  | N | β (95%CI) | p-value |
| 1-3mo | 395 | -0.024(-0.053,0.006) | 0.118 |  | 351 | -0.024(-0.052,0.004) | 0.092 |
| 3-6mo | 475 | -0.004(-0.023,0.016) | 0.717 |  | 465 | -0.009(-0.029,0.010) | 0.355 |
| 6-12mo | 745 | -0.001(-0.011,0.008) | 0.783 |  | 662 | -0.001(-0.016,0.013) | 0.850 |
| 12-18mo | 878 | -0.003(-0.013,0.008) | 0.645 |  | 878 | -0.000(-0.012,0.012) | 0.983 |
| **Lactulose:mannitol Ratio** | | | | | | | |
|  | Unadjusted | | |  | Adjusted | | |
| Interval | N | β (95%CI) | p-value |  | N | β (95%CI) | p-value |
| 3-6mo | 404 | 0.008(-0.017,0.034) | 0.522 |  | 362 | 0.005(-0.020,0.030) | 0.709 |
| 6-12mo | 509 | 0.001(-0.025,0.026) | 0.958 |  | 509 | -0.001(-0.027,0.026) | 0.950 |
| 12-18mo | 536 | -0.003(-0.007,0.001) | 0.143 |  | 513 | -0.012(-0.033,0.009) | 0.270 |
| **Lactulose Excretion Fraction** | | | | | | | |
|  | Unadjusted | | |  | Adjusted | | |
| Interval | N | β (95%CI) | p-value |  | N | β (95%CI) | p-value |
| 3-6mo | 407 | 0.057(-0.183,0.298) | 0.639 |  | 365 | 0.065(-0.205,0.334) | 0.638 |
| 6-12mo | 510 | 0.003(-0.014,0.021) | 0.725 |  | 510 | 0.003(-0.013,0.019) | 0.734 |
| 12-18mo | 553 | 0.003(-0.003,0.009) | 0.377 |  | 529 | -0.003(-0.015,0.010) | 0.662 |
| **Mannitol Excretion Fraction** | | | | | | | |
|  | Unadjusted | | |  | Adjusted | | |
| Interval | N | β (95%CI) | p-value |  | N | β (95%CI) | p-value |
| 3-6mo | 404 | 0.001(-0.040,0.042) | 0.974 |  | 362 | 0.004(-0.039,0.047) | 0.855 |
| 6-12mo | 509 | 0.006(-0.005,0.016) | 0.268 |  | 509 | 0.006(-0.003,0.016) | 0.203 |
| 12-18mo | 537 | 0.003(-0.013,0.018) | 0.738 |  | 514 | -0.001(-0.020,0.019) | 0.957 |
| **soluble CD14** | | | | | | | |
|  | Unadjusted | | |  | Adjusted | | |
| Interval | N | β (95%CI) | p-value |  | N | β (95%CI) | p-value |
| 1-3mo | 552 | 0.005(-0.030,0.040) | 0.781 |  | 495 | 0.031(-0.006,0.069) | 0.097 |
| 3-6mo | 696 | -0.007(-0.040,0.026) | 0.679 |  | 664 | -0.007(-0.045,0.032) | 0.734 |
| 6-12mo | 797 | -0.001(-0.010,0.007) | 0.758 |  | 797 | 0.001(-0.008,0.010) | 0.866 |
| 12-18mo | 877 | -0.003(-0.018,0.012) | 0.681 |  | 877 | -0.003(-0.019,0.013) | 0.714 |
| **Myeloperoxidase** | | | | | | | |
|  | Unadjusted | | |  | Adjusted | | |
| Interval | N | β (95%CI) | p-value |  | N | β (95%CI) | p-value |
| 1-3mo | 400 | -0.028(-0.054,-0.003) | 0.030 |  | 355 | -0.031(-0.063,0.000) | 0.052 |
| 3-6mo | 483 | -0.005(-0.032,0.023) | 0.745 |  | 473 | 0.002(-0.025,0.029) | 0.903 |
| 6-12mo | 746 | -0.008(-0.018,0.001) | 0.078 |  | 663 | -0.012(-0.022,-0.002) | **0.017** |
| 12-18mo | 877 | 0.012(-0.004,0.027) | 0.157 |  | 877 | 0.013(-0.002,0.029) | 0.084 |
| **Neopterin** | | | | | | | |
|  | Unadjusted | | |  | Adjusted | | |
| Interval | N | β (95%CI) | p-value |  | N | β (95%CI) | p-value |
| 1-3mo | 392 | -0.028(-0.088,0.032) | 0.356 |  | 380 | -0.018(-0.066,0.031) | 0.473 |
| 3-6mo | 469 | -0.009(-0.032,0.014) | 0.443 |  | 459 | -0.002(-0.025,0.021) | 0.885 |
| 6-12mo | 738 | 0.002(-0.005,0.010) | 0.526 |  | 657 | 0.002(-0.007,0.011) | 0.618 |
| 12-18mo | 871 | -0.010(-0.019,-0.000) | 0.044 |  | 871 | -0.003(-0.010,0.003) | 0.312 |
| **EE Score** | | | | | | | |
|  | Unadjusted | | |  | Adjusted | | |
| Interval | N | β (95%CI) | p-value |  | N | β (95%CI) | p-value |
| 1-3mo | 387 | -0.037(-0.073,-0.001) | 0.046 |  | 375 | -0.024(-0.061,0.012) | 0.197 |
| 3-6mo | 463 | -0.016(-0.039,0.007) | 0.163 |  | 453 | -0.011(-0.036,0.014) | 0.391 |
| 6-12mo | 737 | 0.002(-0.009,0.012) | 0.736 |  | 656 | 0.000(-0.010,0.011) | 0.978 |
| 12-18mo | 868 | 0.008(-0.002,0.019) | 0.128 |  | 868 | 0.013(-0.002,0.027) | 0.094 |
| **C-reactive Protein** | | | | | | | |
|  | Unadjusted | | |  | Adjusted | | |
| Interval | N | β (95%CI) | p-value |  | N | β (95%CI) | p-value |
| 1-3mo | 552 | -0.010(-0.054,0.034) | 0.668 |  | 495 | -0.004(-0.039,0.031) | 0.829 |
| 3-6mo | 696 | -0.003(-0.021,0.015) | 0.750 |  | 664 | -0.004(-0.022,0.014) | 0.631 |
| 6-12mo | 797 | 0.002(-0.015,0.018) | 0.855 |  | 797 | 0.002(-0.014,0.019) | 0.787 |
| 12-18mo | 877 | 0.011(-0.000,0.022) | 0.055 |  | 877 | 0.011(0.001,0.021) | **0.037** |
| **Kynurenine:Tryptophan Ratio** | | | | | | | |
|  | Unadjusted | | |  | Adjusted | | |
| Interval | N | β (95%CI) | p-value |  | N | β (95%CI) | p-value |
| 1-3mo | 470 | 0.004(-0.022,0.030) | 0.757 |  | 423 | 0.006(-0.020,0.032) | 0.645 |
| 3-6mo | 614 | 0.038(-0.013,0.089) | 0.149 |  | 586 | 0.040(-0.024,0.105) | 0.220 |
| 6-12mo | 725 | 0.003(-0.008,0.014) | 0.567 |  | 725 | 0.004(-0.007,0.015) | 0.503 |
| 12-18mo | 796 | 0.007(-0.004,0.017) | 0.198 |  | 752 | 0.003(-0.010,0.016) | 0.644 |
| **Kynurenine** | | | | | | | |
|  | Unadjusted | | |  | Adjusted | | |
| Interval | N | β (95%CI) | p-value |  | N | β (95%CI) | p-value |
| 1-3mo | 526 | 0.008(-0.021,0.038) | 0.570 |  | 470 | 0.006(-0.024,0.035) | 0.704 |
| 3-6mo | 660 | 0.040(0.000,0.079) | 0.048 |  | 629 | 0.044(-0.007,0.096) | 0.093 |
| 6-12mo | 745 | 0.011(-0.000,0.022) | 0.054 |  | 745 | 0.009(-0.002,0.021) | 0.102 |
| 12-18mo | 819 | -0.008(-0.027,0.012) | 0.443 |  | 774 | -0.008(-0.026,0.011) | 0.423 |
| **Tryptophan** | | | | | | | |
|  | Unadjusted | | |  | Adjusted | | |
| Interval | N | β (95%CI) | p-value |  | N | β (95%CI) | p-value |
| 1-3mo | 526 | -0.003(-0.037,0.031) | 0.856 |  | 470 | -0.005(-0.038,0.027) | 0.762 |
| 3-6mo | 660 | -0.002(-0.034,0.029) | 0.886 |  | 629 | -0.002(-0.034,0.031) | 0.917 |
| 6-12mo | 745 | -0.001(-0.011,0.010) | 0.903 |  | 745 | -0.003(-0.013,0.008) | 0.622 |
| 12-18mo | 819 | -0.013(-0.034,0.007) | 0.197 |  | 774 | -0.010(-0.028,0.007) | 0.243 |
| **Insulin-like growth factor-1** | | | | | | | |
|  | Unadjusted | | |  | Adjusted | | |
| Interval | N | β (95%CI) | p-value |  | N | β (95%CI) | p-value |
| 1-3mo | 550 | 0.027(-0.003,0.056) | 0.076 |  | 493 | 0.030(-0.000,0.061) | 0.054 |
| 3-6mo | 695 | -0.011(-0.035,0.013) | 0.377 |  | 663 | -0.013(-0.037,0.011) | 0.297 |
| 6-12mo | 797 | 0.003(-0.018,0.025) | 0.762 |  | 797 | 0.002(-0.020,0.024) | 0.849 |
| 12-18mo | 877 | -0.009(-0.019,0.002) | 0.102 |  | 877 | -0.002(-0.013,0.009) | 0.687 |
|  |  |  |  |  |  |  |  |

**Supplementary Table 16.** Candidate variables selected by best subset selection for our main model with LAZ velocity (sd/month) as the outcome (Table 3 in paper).

| **Biomarker** | **1-3mo** | **3mo-6mo** | **6-12mo** | **12-18mo** |
| --- | --- | --- | --- | --- |
| IFABP | - Infant Sex - Infant age - Starting WHZ - Maternal social support | - Infant Sex - Infant age - Starting WHZ | - Infant Sex - Infant age - Starting WHZ - Maternal ever booked for antenatal care - Maternal age - Infant birth weight | - Infant Sex - Infant age - Infant consumes minimally diverse diet |
| CIT | - Infant Age - Infant Sex - Starting WHZ - Maternal social support | - Infant Age - Infant Sex - Starting WHZ | - Infant Age - Infant Sex - Starting WHZ - Maternal ever booked for antenatal care | - Infant Age - Infant Sex - Household wealth score |
| REG1B | - Infant Sex - Infant age - Starting WHZ - Maternal height | - Infant Sex - Infant age - Starting WHZ - Delivery mode | - Infant Sex - Infant age - Starting WHZ | - Infant Sex - Infant age - Infant Consumes Minimally Diverse Diet |
| A1AT | - Infant Age - Infant Sex - Starting WHZ - Household Size | - Infant Age - Infant Sex - Starting WHZ - Delivery occurred during hungry season - Delivery mode | - Infant Age - Infant Sex - Starting WHZ - Maternal Age | - Infant Age - Infant Sex - Infant consumes minimally diverse diet |
| LM Ratio |  | - Infant Sex - Infant age - Starting WHZ - Household food insecurity | - Infant Sex - Infant age - Starting WHZ - Maternal ever booked antenatal care | - Infant Sex - Infant age - Maternal gender norm attitudes |
| Lactulose excretion fraction |  | - Infant Sex - Infant age - Starting WHZ - Household food insecurity | - Infant Sex - Infant age - Starting WHZ - Maternal ever booked antenatal care | - Infant Sex - Infant age - Maternal gender norm attitudes |
| Mannitol excretion fraction |  | - Infant Sex - Infant age - Starting WHZ - Household food insecurity | - Infant Sex - Infant age - Starting WHZ | - Infant Sex - Infant age - Maternal gender norm attitudes |
| sCD14 | - Infant Sex - Infant age - Starting WHZ - Maternal social support | - Infant Sex - Infant age - Starting WHZ | - Infant Sex - Infant age - Starting WHZ - Maternal ever booked antenatal care - Maternal age - Infant birth weight | - Infant Sex - Infant Age - Starting WHZ - Infant Consumes Minimally Diverse Diet |
| MPO | - Infant Sex - Infant age - Starting WHZ | - Infant Sex - Infant age - Starting WHZ - Delivery mode - Household food insecurity | - Infant Sex - Infant age - Starting WHZ - Maternal age - Infant birth weight | - Infant Sex - Infant age - Starting WHZ - Infant consumes minimally diverse diet |
| NEO | - Infant Sex - Infant age - Starting WHZ - Household size | - Infant Sex - Infant age - Starting WHZ - Delivery mode | - Infant Sex - Infant age - Starting WHZ - Maternal decision-making autonomy | - Infant Sex - Infant age - Infant consumes minimally diverse diet |
| Kosek EE Score | - Infant Sex - Infant age - Starting WHZ - Household size | - Infant Sex - Infant age - Starting WHZ - Delivery mode | - Infant Sex - Infant age - Starting WHZ - Maternal decision-making autonomy | - Infant Sex - Infant age - Starting WHZ - Infant consumes minimally diverse diet |
| CRP | - Infant Sex - Infant Sex - Starting WHZ - Maternal social support | - Infant Age - Infant Sex - Starting WHZ | - Infant Age - Infant Sex - Starting WHZ - Maternal ever booked for antenatal care - Maternal age - Infant birth weight | - Infant Age - Infant Sex - Infant consumes minimally diverse diet |
| KT Ratio | - Infant Sex - Infant age - Starting WHZ - Maternal social support | - Infant Sex - Infant age - Starting WHZ | - Infant Sex - Infant age - Starting WHZ - Maternal Ever booked for antenatal care | - Infant Sex - Infant age - Starting WHZ - Household wealth score |
| Kynurenine | - Infant Sex - Infant age - Starting WHZ - Maternal social support | - Infant Sex - Infant age - Starting WHZ | - Infant Sex - Infant age - Starting WHZ - Maternal ever booked antenatal care - Infant birth weight | - Infant Sex - Infant age - Household wealth score |
| Tryptophan | - Infant Sex - Infant age - Starting WHZ - Maternal social support | - Infant Sex - Infant age - Starting WHZ | - Infant Sex - Infant age - Starting WHZ - Maternal ever booked antenatal care - Maternal age - Infant birth weight | - Infant Sex - Infant age - Household wealth score |
| IGF-1 | - Infant Sex - Infant age - Starting WHZ - Maternal social support | - Infant Sex - Infant age - Starting WHZ | - Infant Sex - Infant age - Starting WHZ - Maternal ever booked for antenatal care - Maternal age - Infant birth weight | - Infant Sex - Infant age - Infant consumes minimally diverse diet |


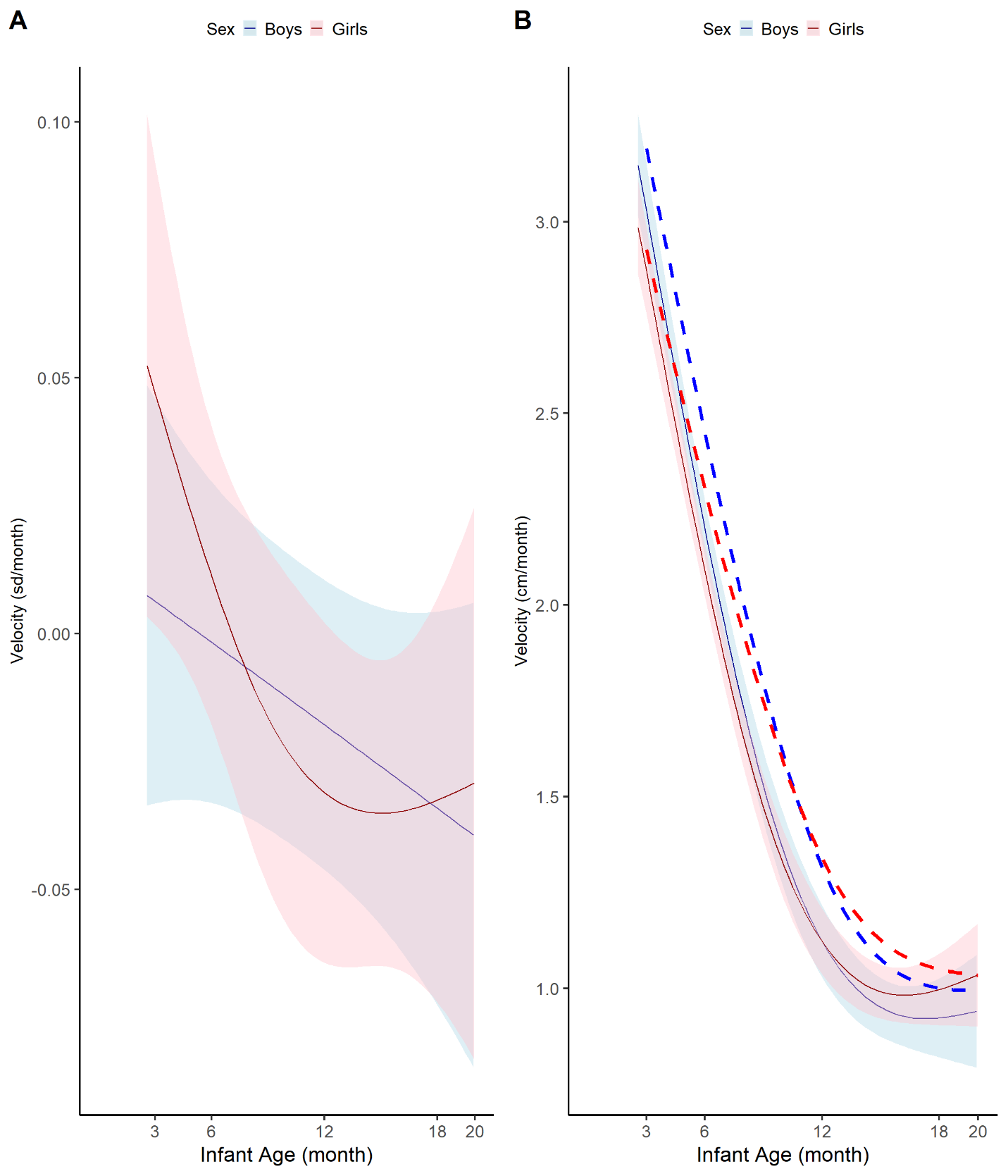


**Supplementary Figure 1.** LAZ velocity (A) and length velocity (B) over by infant age among girls (pink) and boys (blue) in SHINE. Dotted lines in panel B are median values from World Health Organization reference growth standards. Modelled using generalized additive models of growth velocity against infant age at the end of the follow-up interval. Graphs are smoothed using cubic splines with 3-5 knots.
